# Early detection of sepsis using machine learning algorithms: a systematic review and network meta-analysis

**DOI:** 10.3389/fmed.2024.1491358

**Published:** 2024-10-16

**Authors:** Mikhail Ya Yadgarov, Giovanni Landoni, Levan B. Berikashvili, Petr A. Polyakov, Kristina K. Kadantseva, Anastasia V. Smirnova, Ivan V. Kuznetsov, Maria M. Shemetova, Alexey A. Yakovlev, Valery V. Likhvantsev

**Affiliations:** ^1^Federal Research and Clinical Centre of Intensive Care Medicine and Rehabilitology, Moscow, Russia; ^2^Department of Anaesthesia and Intensive Care, IRCCS San Raffaele Scientific Institute, Milan, Italy; ^3^Department of Anesthesiology, Vita-Salute San Raffaele University, Milan, Italy; ^4^Department of Anesthesiology, I.M. Sechenov First Moscow State Medical University, Moscow, Russia

**Keywords:** sepsis, machine learning, network meta-analysis, decision trees, neural networks

## Abstract

**Background:**

With machine learning (ML) carving a niche in diverse medical disciplines, its role in sepsis prediction, a condition where the ‘golden hour’ is critical, is of paramount interest. This study assesses the factors influencing the efficacy of ML models in sepsis prediction, aiming to optimize their use in clinical practice.

**Methods:**

We searched Medline, PubMed, Google Scholar, and CENTRAL for studies published from inception to October 2023. We focused on studies predicting sepsis in real-time settings in adult patients in any hospital settings without language limits. The primary outcome was area under the curve (AUC) of the receiver operating characteristic. This meta-analysis was conducted according to PRISMA-NMA guidelines and Cochrane Handbook recommendations. A Network Meta-Analysis using the CINeMA approach compared ML models against traditional scoring systems, with meta-regression identifying factors affecting model quality.

**Results:**

From 3,953 studies, 73 articles encompassing 457,932 septic patients and 256 models were analyzed. The pooled AUC for ML models was 0.825 and it significantly outperformed traditional scoring systems. Neural Network and Decision Tree models demonstrated the highest AUC metrics. Significant factors influencing AUC included ML model type, dataset type, and prediction window.

**Conclusion:**

This study establishes the superiority of ML models, especially Neural Network and Decision Tree types, in sepsis prediction. It highlights the importance of model type and dataset characteristics for prediction accuracy, emphasizing the necessity for standardized reporting and validation in ML healthcare applications. These findings call for broader clinical implementation to evaluate the effectiveness of these models in diverse patient groups.

**Systematic review registration:**

https://inplasy.com/inplasy-2023-12-0062/, identifier, INPLASY2023120062.

## 1 Introduction

Sepsis is a critical medical condition characterized by a substantial risk of mortality (1). Prompt identification of sepsis is crucial for the successful treatment of this life-threatening condition. Adhering to the ‘golden hour’ principle, which suggests that patient outcomes are significantly improved when treatment is initiated within the first hour following diagnosis, is pivotal for enhancing patient survival rates. Concurrently, there is a robust endorsement for employing systematic screening procedures for early sepsis identification (2).

The accuracy of current clinical scales and diagnostic methodologies in detecting and predicting sepsis seems to be significantly suboptimal, leading to delays in therapeutic interventions (3–5). Despite the widespread use of traditional sepsis scoring systems, such as SOFA, NEWS, MEWS, SIRS, and SAPS II, these tools exhibit several limitations, including their reliance on static thresholds and suboptimal predictive performance. As a result, traditional sepsis scoring systems often lack the sensitivity and specificity required for timely, accurate sepsis detection.

This gap underscores the urgent need for more precise and reliable diagnostic and prognostic tools. In this regard, there is a shift in focus towards innovative approaches such as machine learning (6–13). Particularly, right-aligned models are drawing significant attention for their capacity to predict the development of sepsis hours before its clinical confirmation (14). Evidence increasingly suggests that machine learning methodologies offer a distinct advantage over traditional sepsis scoring systems (6).

To date, three meta-analyses have been conducted in this area of study (6, 15, 16), with one demonstrating the superiority of machine learning over traditional clinical scales in sepsis prognosis (6). In second research, the evidence presented lacks robustness (16), whereas in a third investigation, the focus was solely on the comparative assessment of different machine learning methodologies (15). However, the significant clinical heterogeneity, not entirely unambiguous diagnostic criteria, diverse prognostic time frames, and differing approaches to data preprocessing and model development across patient populations preclude definitive conclusions about the prognostic efficacy of these machine learning models.

In response to these challenges, our objective was to conduct a pioneering network meta-analysis to address this heterogeneity and to surpass the confines of previous research. Through meta-regression, we aimed to identify key factors that influence the effectiveness of predictive models, thereby guiding the development of an optimal model for sepsis prognosis, tailored to the complexities of clinical scenarios.

## 2 Materials and methods

This study was conducted in accordance with the Preferred Reporting Items for Systematic Reviews and Meta-Analyses (PRISMA) Extension Statement for Reporting of Systematic Reviews Incorporating Network Meta-analyses of Health Care Interventions (PRISMA-NMA) guidelines (17) and the Cochrane Handbook recommendations (18). The study protocol was registered with the International Platform of Registered Systematic Review and Meta-analysis Protocols (INPLASY) under the registration number INPLASY 2023120062 (doi: 10.37766/inplasy2023.12.0062). The completed PRISMA-NMA checklist is presented in Supplementary Table S1.

### 2.1 Search strategy

We performed a systematic search of the literature across Medline, PubMed, Google Scholar, and the Cochrane Central Register of Controlled Trials (CENTRAL) from inception to October 2023. The search was conducted by two independent investigators. Backward and forward citation tracking was also employed to identify additional studies, leveraging the Litmaps service (19). No language restrictions were applied. Details of the search strategy, including full queries, are provided in Supplementary Appendix A.

### 2.2 Eligibility criteria and study selection

Following the automatic removal of duplicate records, two independent researchers screened the remaining studies for eligibility. We applied the PICOS (Population, Intervention, Comparator, Outcome, and Study design) framework to guide study selection (Supplementary Appendix B).

Studies were considered eligible if they focused on real-time prediction of sepsis onset (right alignment (14)) in adult patients across any hospital setting. Both prospective and retrospective diagnostic test accuracy studies were included. The target condition was the onset of sepsis, defined by Sepsis-3 criteria (20) or other operational definitions provided by the authors (Supplementary Table S5).

Studies were excluded if they met one of the following criteria: (1) were review articles, case reports or case series without control groups; (2) had no sepsis definition criteria; (3) reported no AUC for sepsis development; (4) reported AUC for other outcome (e.g., reported data on mortality only); (5) focused on pediatric patients; (6) reported no data on patient cohort (sample size, age, sex, etc.); (7) were published as conference papers or preprints only.

Any disagreement was solved by consultation until consensus was reached. Divergences were resolved by consensus with the involvement of the supervisor.

### 2.3 Outcome measures and data extraction

A standardized data collection form was developed specifically for this review. Three independent authors used this form to systematically evaluate the full text, supplemental materials, and additional files of all included studies. Data extraction was performed independently by three authors, with any discrepancies resolved through discussion to achieve consensus.

Extracted information encompassed: (1) Basic study details such as the first author, publication year, country, journal, study design, data collection period, mean age, sex, hospital mortality, prediction method, and sample size; (2) ML model characteristics: data source, prediction model, sepsis definition criteria, department, prediction window, external validation, imputation, features; (3) Outcome data: area under the curve of the receiver operating characteristic (AUC) as performance metric.

ML prediction models were grouped as detailed in Supplementary Appendix C.

In an attempt to reduce the number of comparisons, when multiple models from the same group were used in a single article (employing different factor selection and optimization methods), the analysis focused on the highest AUC value. The standard deviation (SD) for AUC was either extracted directly from the article, requested from the authors, converted from the 95% confidence interval (CI) according to the Cochrane Handbook recommendations, or imputed using the Iterative Imputer algorithm based on a Bayesian regression model (Python’s sklearn library).

Data on the AUC metrics for traditional scoring systems were also extracted if available. These systems included SOFA (Sequential Organ Failure Assessment), qSOFA (quick SOFA), NEWS/NEWS2 (National Early Warning Score), MEWS (Modified Early Warning Score), SAPS II (Simplified Acute Physiology Score), and SIRS (Systemic Inflammatory Response Syndrome). In cases where multiple traditional scoring systems were used, the best metric was considered.

### 2.4 Data analysis and synthesis

Traditional meta-analysis was conducted to calculate pooled AUCs. Inter-study heterogeneity was evaluated using the I-squared (I2) statistic and the Cochrane Q test; random-effects model (restricted maximum–likelihood, REML) was used. Statistical significance was set at 0.05 for hypothesis testing. We conducted a meta-regression analysis, leveraging the REML random-effects model, to ascertain if the AUC metrics might be affected by covariates such as study design and ML model characteristics (21). All covariates were first tested in a univariate model, significant covariates were then considered for a multivariable model. The results of the meta-regression were graphically represented using bubble-plots.

We also conducted a frequentist, random-effects Network Meta-Analysis (NMA) using CINeMA (Confidence in Network Meta-Analysis) approach (22), CINeMA software (23), ROB-MEN web application (24) and STATA 17.0 (StataCorp, College Station, TX) software. Articles were included in the NMA if they compared any two ML models with different ML models or any ML model with a traditional scoring system. The Mean Difference (MD) with corresponding 95% CI was calculated for AUCs. Results of NMA were presented using network plots, league tables, contribution tables and NMA forest plots. To assess between-study heterogeneity, we utilized Bayesian NMA with τ2 calculation. A τ2 value exceeding the clinically important effect size (MD ≥ 0.15) indicated significant heterogeneity.

### 2.5 Internal validity and risk of bias assessment

The internal validity and risk of bias were assessed by three independent reviewers (MY, AS, IK) using the ‘Quality Assessment of Diagnostic Accuracy Studies’ (QUADAS-2) tool (25) combined with an adapted version of the ‘Joanna Briggs Institute Critical Appraisal checklist for analytical cross-sectional studies’ (26) (Supplementary Table S2). Publication bias and small-study effects were assessed using Bayesian NMA meta-regression and funnel plot analysis (for comparisons with 10 or more studies). The certainty of evidence was assessed with GRADE methodology integrated in CINeMA approach. We conducted a sensitivity analysis using studies with low to moderate risk of bias.

## 3 Results

### 3.1 Study characteristics

**Figure 1:**
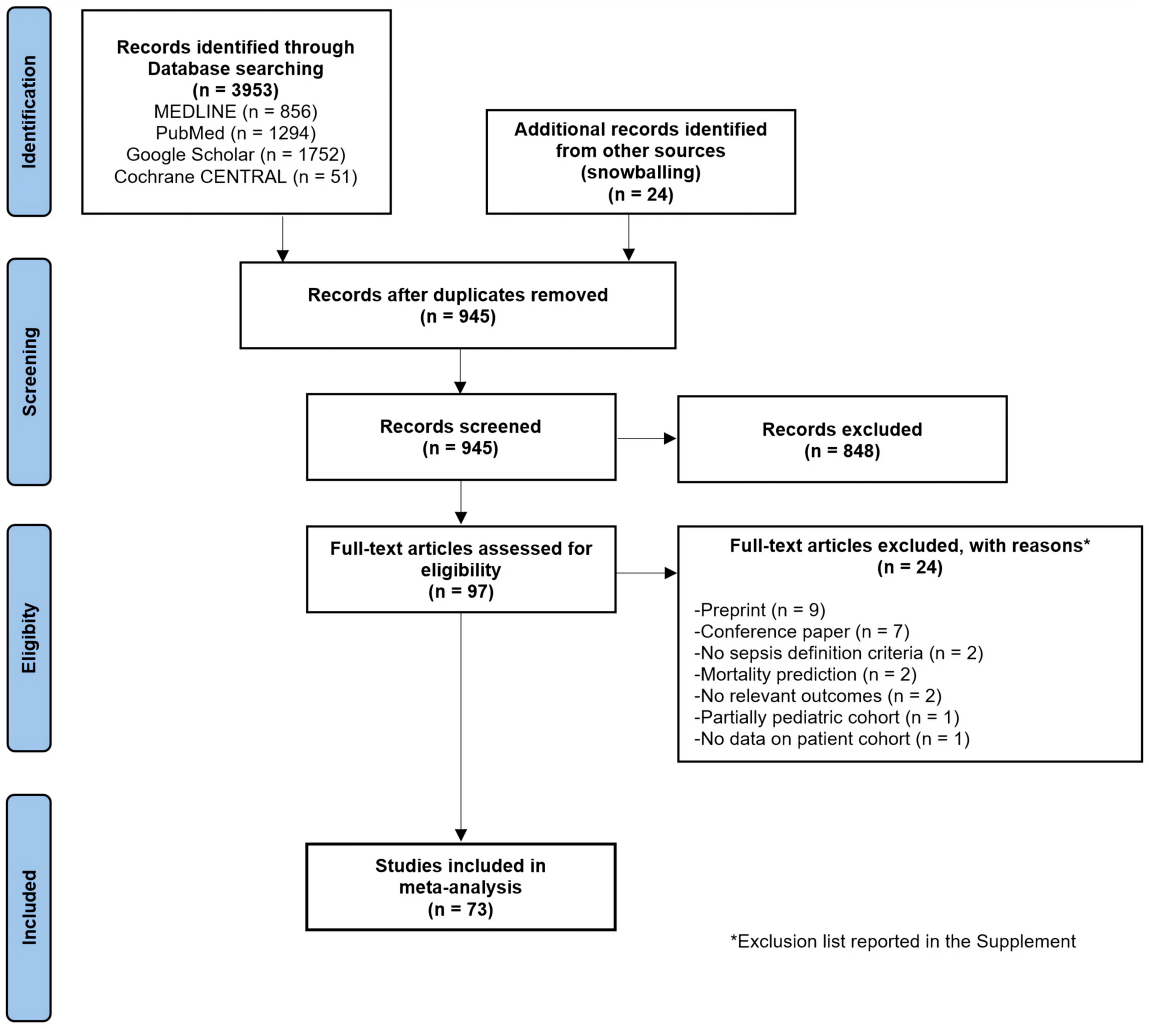
PRISMA flow diagram for study selection.

The initial literature search identified 3,953 studies from various databases, with an additional 24 studies from other sources (Figure 1). After removing duplicates and abstract screening, 97 papers underwent eligibility screening. A total of 256 models from 73 studies (457,932 septic patients) were included (14, 27–98) with major exclusions list presented in Supplementary Table S1. The specialty journal with the largest number of articles was Critical Care Medicine (39, 41, 53, 62, 87).

Most of the studies included in the analysis were conducted in the ICU (*n* = 49; 67.1%), followed by hospital wards (*n* = 12; 16.4%) and emergency departments (ED, *n* = 9; 12.3%) (Supplementary Tables S4–S8). The median sepsis prevalence across the studies was 14.3% (IQR 7.3–32.4%), with the mean patient age ranging from 35 to 70 years. The median (IQR) mortality rate was 2.3% (6.9–14.8%). Sepsis was most frequently defined by the Sepsis-3 criteria (57.5%), with other definitions including Sepsis-2, ICD-9, ICD-10, and SIRS. Prediction windows varied widely, ranging from immediate (0 h) to 7 days. External validation was performed in 16 studies (21.9%), and imputation techniques were employed in 44 studies (60.3%). Notably, 53% of studies utilized public datasets such as MIMIC, eICU, and the Computing in Cardiology Challenge 2019, while the remaining studies relied on proprietary hospital datasets.

### 3.2 Pooled AUCs

The pooled AUC for machine learning models was 0.825 (95% CI 0.809–0.840, *p* < 0.001) across 73 studies (Supplementary Table S9). In comparison, the AUC for the SOFA score was 0.667 (95% CI 0.586–0.748) across 17 studies, for qSOFA 0.612 (95% CI 0.574–0.650) across 16 studies, for NEWS/NEWS2 0.719 (95% CI 0.674–0.764) across 9 studies, for MEWS 0.651 (95% CI 0.612–0.690) across 12 studies, for SIRS 0.666 (95% CI 0.643–0.688) across 19 studies, and for SAPS II 0.662 (95% CI 0.589–0.736) across 2 studies (all *p* < 0.001, Supplementary Table S9). Heterogeneity across studies was high (I^2^ > 95%, *p* < 0.001).

### 3.3 Network meta-analysis

#### 3.3.1 ML vs. scoring systems

**Figure 2:**
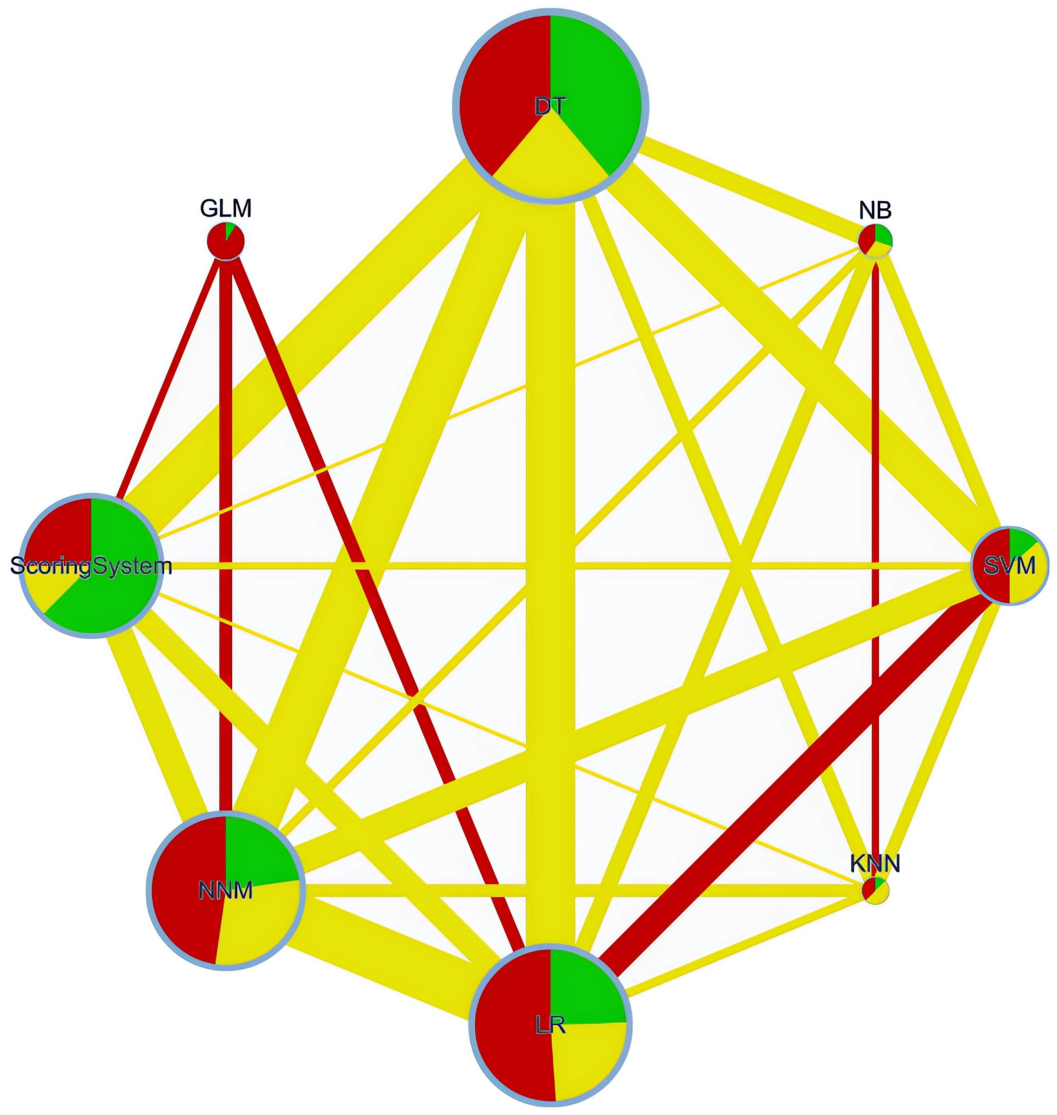
Network plot. DT, Decision Tree; NNM, Neural Network Model; SVM, Support Vector Machine; LR, Logistic Regression; NB, Naïve Bayes; GLM, Generalized Linear Model; KNN, K-Nearest Neighbors.

**Figure 3:**
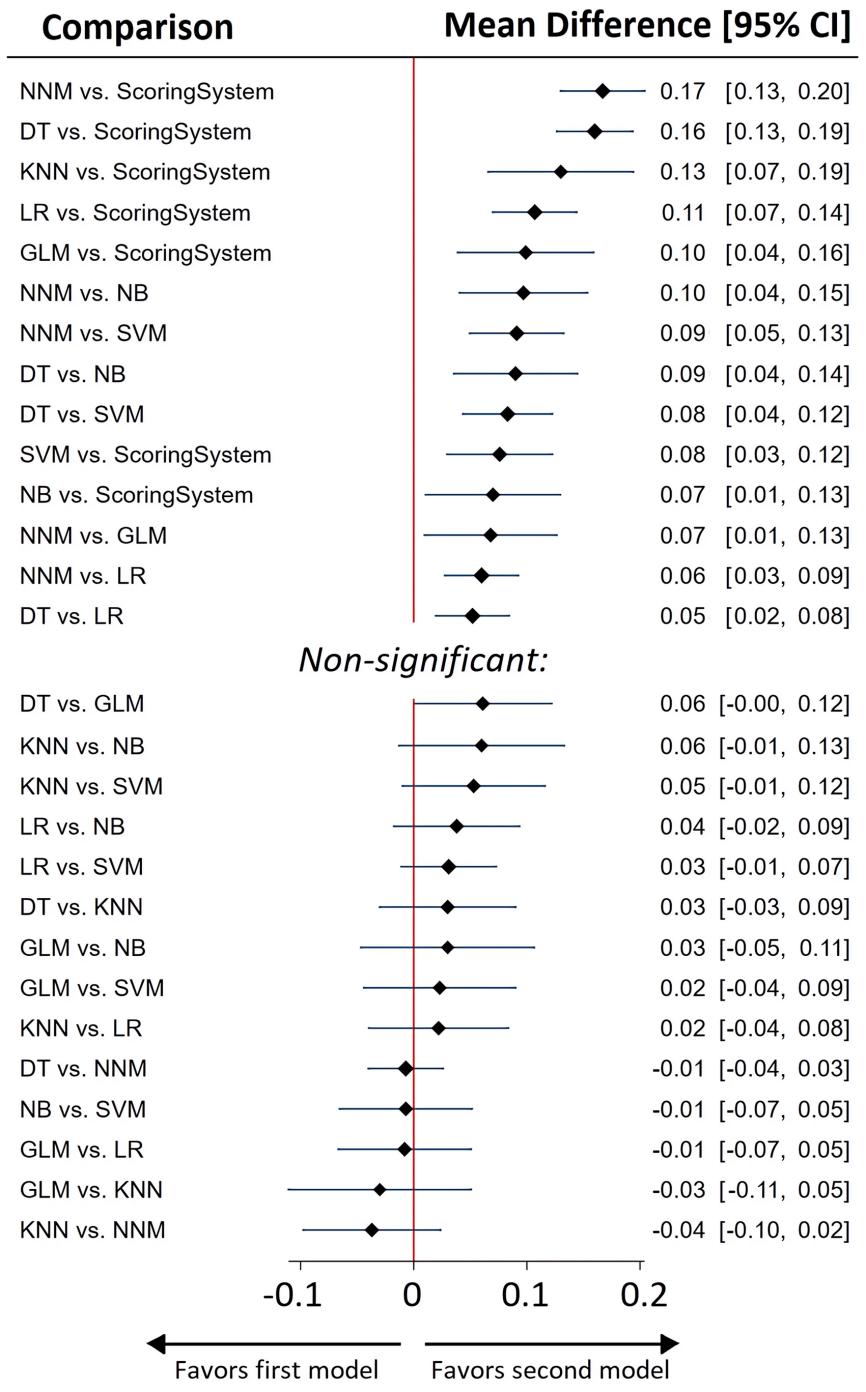
Network meta-analysis summary forest plot for predictive efficacy of various ML models and best of scoring system used for sepsis prediction.

All ML models exhibited a significant performance advantage over traditional scoring systems when performing a NMA (Figures 2, 3, Supplementary Tables S10–S12).

*A network plot is a visual tool in network meta-analyses, showing interventions (groups) as nodes and their direct comparisons as connecting lines. This visual tool helps in understanding the complex relationships and the extent of evidence available for each comparison in the network meta-analysis.*

*Network of retrospective diagnostic test accuracy studies comparing the AUCs of* var*ious machine learning models and best of scoring system used. The size of nodes and width of the edges are proportional to the number of studies. The colors of edges and nodes refer to the average risk of bias: low (green), moderate (yellow), and high (red).*

#### 3.3.2 ML models

As indicated by the NMA results, Neural Network Models (NNM) and Decision Tree (DT) models exhibited the highest AUC metrics (Figure 3, Supplementary Tables S11, S12).

### 3.4 Meta-regression

In the multivariable model, only ML model type, dataset type (with non-freely available hospital datasets showing higher AUCs), and prediction window (showing a negative association) had significant contributions to the AUC. (Supplementary Table S13, Supplementary Figures S1, S2).

### 3.5 Risk of bias and GRADE assessment

The overall risk of bias of the 73 enrolled studies was judged as ‘low’ in 29 studies, with ‘some concerns’ in 14 studies and ‘high’ in 30 studies (Supplementary Figure S3). The main sources of bias identified were insufficient description of the study population and data sources, along with the use of different sepsis definitions.

Risk of bias bar chart is presented in Supplementary Figure S4. Publication bias and small-study effects assessment results are summarized in Supplementary Table S14 and Supplementary Figure S5. Between-study variance was not significant (τ^2^ = 0.095, with the clinically important effect size stated as 0.1). Contribution matrices are presented in Supplementary Tables S15, S16.

The CINeMA ratings can be found in Supplementary Figure S6. The level of evidence supporting the superiority of ML models over traditional scoring systems was categorized as ‘low’.

## 4 Discussion

### 4.1 Key findings

This network meta-analysis is the first to comprehensively evaluate the performance of (ML) models in sepsis prediction, demonstrating that ML algorithms, particularly neural network models and decision trees, significantly outperform traditional scoring systems. These findings underscore the enhanced ability of ML models to analyse and interpret complex clinical data, pointing to a potential paradigm shift in sepsis prediction strategies.

A critical aspect of our findings relates to the impact of the type of ML model and the nature of the dataset on model performance. The choice of the ML model itself emerged as a significant determinant of model performance in our study. This finding indicates that the inherent characteristics and algorithms of different ML models substantially influence their ability to predict sepsis effectively. Models utilizing freely available datasets exhibited lower AUCs, which could be a result of overfitting in models based on non-freely available hospital datasets.

Another key finding from our study is the temporal dynamics in prediction accuracy. We observed a negative association between prediction window and AUC, indicating that ML models are more accurate in short-term than in long-term sepsis prediction.

Interestingly, factors such as the size of the training dataset, sepsis prevalence, the department in which the study was conducted, the presence of imputation and external validation, the use of laboratory indicators, and the number of predictors did not significantly influence the quality of the predictive models.

### 4.2 Relationship with previous studies

The results of our systematic review and meta-analysis can be compared with those of 3 previous meta-analyses. Islam et al. in 2019 were the first demonstrated that ML approach outperforms existing scoring systems in predicting sepsis (6). Fleuren et al. (15) suggested that ML models can accurately predict the onset of sepsis with good discrimination in retrospective cohorts, and this study was the first to indicate that the choice of ML model could impact AUC. The authors also suggested that NNM had advantages over DT, and that the inclusion of body temperature and laboratory indicators enhanced prediction quality. The only other meta-analysis performed so far demonstrated the superiority of XGBoost and random forest models but with high heterogeneity (I2) (16). In other systematic reviews, quantitative meta-analysis was not conducted due to significant heterogeneity among studies (10–13). We were the first to apply a NMA technique, which allowed us to overcome high heterogeneity of previous meta-analyses. This approach enables comparisons between two ML models or an ML model and a traditional scoring system within the same study on a single patient cohort, employing a unified approach and standardized definition of sepsis. In our research, NNM did not demonstrate superiority over DT, and the use of body temperature and laboratory indicators as predictors did not enhance the predictive quality.

### 4.3 Significance of the study findings

Our network meta-analysis, which evaluated 73 articles encompassing 457,932 septic patients, revealed that ML algorithms significantly outperform traditional sepsis scoring systems. The integration of ML into sepsis prediction marks a significant step forward in improving the early diagnosis and management of this life-threatening condition in emergency and intensive care settings. Unlike traditional scoring systems, ML models can process vast amounts of real-time clinical data, offering early warning systems that may identify sepsis before the appearance of clinical symptoms, thereby facilitating timely and targeted interventions. This has profound implications for clinical practice, as prompt treatments such as early antibiotic administration are known to significantly improve patient outcomes, particularly when initiated within the ‘golden hour’ (3–5).

Our study makes a key contribution by identifying the factors that impact the effectiveness of sepsis prediction models. Specifically, we found that the number of predictors and sepsis prevalence do not substantially influence model performance, challenging the traditional assumption that larger datasets and a perfectly balanced cohort (with a 50/50% split) are essential for robust predictions. Instead, our findings underscore the importance of data quality and the careful selection of relevant predictors, which has direct implications for how ML models should be developed and deployed in real-world clinical environments.

The heterogeneity in external validation and imputation methods across studies underscores a significant gap in standardizing ML model development and validation for sepsis prediction. While we did not find a notable impact of external validation on AUC, its role in enhancing the robustness and generalizability of prediction models should not be underestimated. Furthermore, it’s noteworthy that even in the context of established and stringent diagnostic guidelines for sepsis, there exists a number of studies where researchers have opted to utilize alternative definitions of sepsis in their studies.

Another notable aspect of our research pertains to the ‘black box’ nature of some ML models, which limits the clinician’s ability to understand the logic behind decision-making. Our study demonstrates that the use of DT is not inferior to the more complex NNM. This is a pivotal finding as DT models offer greater transparency in decision-making processes, which is crucial for clinical applications where understanding the rationale behind predictions is as important as the predictions themselves.

While ML models show substantial promise in predicting sepsis onset, their clinical utility remains limited by the challenge of initiating treatment before the appearance of clinical symptoms.

### 4.4 Strengths and limitations

This research is the first to quantitatively demonstrate the superiority of ML models over traditional scoring systems using NMA. Furthermore, this study is the first to employ NMA to reveal the advantages of NNM and DT over other ML models in the prediction of sepsis. Through meta-regression, we identified several critical factors that influence model performance, providing valuable insights for future model development. The application of the CINeMA approach provided a structured methodology to rate the certainty of our evidence, enhancing the reliability of our findings.

However, limitations of our study must be acknowledged: we found high clinical heterogeneity among the included studies and therefore used random-effects modelling and sensitivity analyses; while the AUC was pragmatically chosen as the summary measure, it may not be as effective in imbalanced datasets, yet it remains the most frequently reported measure in this field.

### 4.5 Future studies and prospects

The growing body of evidence supporting the advantages of ML models over traditional scoring systems in sepsis prediction underscores the need to integrate these technologies into routine clinical practice. Future research should focus on conducting well-structured prospective trials to evaluate how ML-predicted sepsis outcomes influence the timing and initiation of antibiotic therapy. A critical component of these trials will be assessing the time interval between ML model predictions and clinical recognition by healthcare providers, as delays in treatment initiation can significantly affect patient outcomes.

We propose a randomized, double-blind controlled trial comparing the efficacy of early antibiotic therapy initiated based on ML predictions versus placebo during this pre-recognition window. Such a study could provide definitive evidence regarding the clinical utility of ML-based early warning systems and their potential to reduce mortality and morbidity in sepsis by enabling earlier interventions.

## 5 Conclusion

Our systematic review and network meta-analysis revealed that machine learning models, specifically neural network models and decision trees, exhibit superior performance in predicting sepsis compared to traditional scoring systems. This study highlights the significant impact of machine learning model type and dataset characteristics on prediction accuracy. Despite the promise of machine learning models in clinical settings, their potential is yet to be fully realized due to study heterogeneity and the variability in sepsis definitions. To bridge this gap, there is an urgent need for standardized reporting and validation frameworks, ensuring that machine learning tools are both reliable and generalizable in diverse clinical settings.

## Data Availability

The original contributions presented in the study are included in the article/Supplementary material, further inquiries can be directed to the corresponding author.
