# Supplementary material for: Early detection of sepsis using machine learning algorithms: a systematic review and network meta-analysis
Source: Front Med (Lausanne). 2024 Oct 16;11:1491358. doi: 10.3389/fmed.2024.1491358 (PMC11523135; doi:10.3389/fmed.2024.1491358)
Supplement: Supplementary file 1 [file Data_Sheet_1.pdf]

Supplementary Material

1 Supplementary Appendix A

Search strategy for each database.

PubMed, MEDLINE:

#1 (sepsis OR "sepsis prediction" OR "sepsis detection") AND ("machine learning" OR "artificial intelligence" OR "deep learning" OR "decision tree")

#2 "sepsis"[Mesh] AND "machine learning"[tiab] AND "prediction"[tiab]

#3 "sepsis"[Mesh] AND ("deep learning"[tiab] OR "neural networks"[tiab]) AND "detection"[tiab]

#4 "sepsis"[Mesh] AND "artificial intelligence"[tiab] AND ("diagnosis"[Mesh] OR "detection"[tiab])

#5 "sepsis"[Mesh] AND "decision trees"[tiab] AND "prediction"[tiab]

#6 "sepsis"[Mesh] AND ("ensemble methods"[tiab] OR "random forests"[tiab]) AND "prediction"[tiab]

#7 "sepsis"[Mesh] AND ("machine learning algorithms"[tiab] OR "comparative study"[tiab]) AND "prediction"[tiab]

#8 "sepsis"[Mesh] AND ("neural network models"[tiab] OR "deep learning models"[tiab]) AND "prediction"[tiab]

#9 "sepsis"[Mesh] AND "machine learning"[tiab] AND ("outcome prediction"[tiab] OR "prognosis"[tiab])

#1 OR #2 OR #3 OR #4 OR #5 OR #6 OR #7 OR #8 OR #9

Google Scholar: ("sepsis" OR "septic shock") AND ("machine learning" OR "artificial intelligence" OR "deep learning" OR "neural networks") AND ("prediction" OR "forecasting" OR "detection" OR "diagnosis") -review -"meta-analysis"

Cochrane CENTRAL: ("sepsis":ti,ab,kw OR "septic shock":ti,ab,kw OR "systemic inflammatory response syndrome":ti,ab,kw) AND ("machine learning":ti,ab,kw OR "artificial intelligence":ti,ab,kw OR "deep learning":ti,ab,kw OR "neural networks":ti,ab,kw OR "decision tree":ti,ab,kw) AND ("prediction":ti,ab,kw OR "forecasting":ti,ab,kw OR "detection":ti,ab,kw OR "diagnosis":ti,ab,kw)

2 Supplementary Appendix B

PICOS criteria.

Population	Adult patients (without restrictions on age, sex, race, or ethnicity).<br>Patients suspected of having sepsis or presenting with signs and symptoms indicative of sepsis.
Intervention (Index test)	Machine Learning (ML) models developed for the prediction, detection, or diagnosis of sepsis (right alignment). Traditional Scoring Systems used for sepsis diagnosis or prediction, such as SOFA (Sequential Organ Failure Assessment), qSOFA (quick SOFA), NEWS/NEWS2 (National Early Warning Score), MEWS (Modified Early Warning Score), SAPS II (Simplified Acute Physiology Score), SIRS (Systemic Inflammatory Response Syndrome).
Comparator<br>(Reference test)	Sepsis-3 definition or other operational definitions of sepsis used in clinical settings.
Outcomes	The primary outcome measure is the Area Under the Receiver Operating Characteristic Curve (AUC-ROC), assessing the diagnostic accuracy of the ML models and traditional scoring systems in identifying or predicting sepsis.
Study design	Prospective and retrospective diagnostic test accuracy studies

Time range: Jan 01, 2013-Oct 01, 2023

3 Supplementary Appendix C

ML prediction model groups.

(1) Neural Network Models (NNM) = Long Short-Term Memory (LSTM), Convolutional Neural Networks (CNN), Deep Neural Networks (DNN), Gated Recurrent Unit (GRU), Temporal Convolutional Networks (TCN), Multilayer Perceptron (MLP);

(2) Decision Trees (DT) = Random Forest (RF), AdaBoost, XGBoost, LightGBM, Extremely Randomized Trees (ET);

(3) Regression Methods (LR) = Logistic Regression, Cox Regression, Non-linear Regression;

(4) Support Vector Machine (SVM);

(5) K-Nearest Neighbors (KNN);

(6) Generalized Linear Model (GLM);

(7) Naive Bayes (NB).

4 Table S1. PRISMA NMA Checklist.

Section/Topic	Item #	Checklist Item	Reported on Page #
**TITLE**			
Title	1	Identify the report as a systematic review *incorporating a network meta-analysis (or related form of meta-analysis).*	Title page
**ABSTRACT**			
Structured summary	2	Provide a structured summary including, as applicable:<br>**Background:** main objectives<br>**Methods:** data sources; study eligibility criteria, participants, and interventions; study appraisal; and *synthesis methods, such as network meta-analysis.*<br>**Results:** number of studies and participants identified; summary estimates with corresponding confidence/credible intervals; *treatment rankings may also be discussed. Authors may choose to summarize pairwise comparisons against a chosen treatment included in their analyses for brevity.*<br>**Discussion/Conclusions:** limitations; conclusions and implications of findings.<br>**Other:** primary source of funding; systematic review registration number with registry name.	Abstract
**INTRODUCTION**			
Rationale	3	Describe the rationale for the review in the context of what is already known, *including mention of why a network meta-analysis has been conducted.*	Introduction
Objectives	4	Provide an explicit statement of questions being addressed, with reference to participants, interventions, comparisons, outcomes, and study design (PICOS).	Introduction
**METHODS**			
Protocol and registration	5	Indicate whether a review protocol exists and if and where it can be accessed (e.g., Web address); and, if available, provide registration information, including registration number.	Abstract, methods
Eligibility criteria	6	Specify study characteristics (e.g., PICOS, length of follow-up) and report characteristics (e.g., years considered, language, publication status) used as criteria for eligibility, giving rationale. *Clearly describe eligible treatments included in the treatment network, and note whether any have been clustered or merged into the same node (with justification).*	Methods, Supplement (PICOS)
Information sources	7	Describe all information sources (e.g., databases with dates of coverage, contact with study authors to identify additional studies) in the search and date last searched.	Methods
Search	8	Present full electronic search strategy for at least one database, including any limits used, such that it could be repeated.	Methods, Supplement
Study selection	9	State the process for selecting studies (i.e., screening, eligibility, included in systematic review, and, if applicable, included in the meta-analysis).	Methods
Data collection process	10	Describe method of data extraction from reports (e.g., piloted forms, independently, in duplicate) and any processes for obtaining and confirming data from investigators.	Methods
Data items	11	List and define all variables for which data were sought (e.g., PICOS, funding sources) and any assumptions and simplifications made.	Methods
Geometry of the network	S1	Describe methods used to explore the geometry of the treatment network under study and potential biases related to it. This should include how the evidence base has been graphically summarized for presentation, and what characteristics were compiled and used to describe the evidence base to readers.	Methods, Fig. 2
Risk of bias within individual studies	12	Describe methods used for assessing risk of bias of individual studies (including specification of whether this was done at the study or outcome level), and how this information is to be used in any data synthesis.	Methods
Summary measures	13	State the principal summary measures (e.g., risk ratio, difference in means). *Also describe the use of additional summary measures assessed, such as treatment rankings and surface under the cumulative ranking curve (SUCRA) values, as well as modified approaches used to present summary findings from meta-analyses.*	Methods
Planned methods of analysis	14	Describe the methods of handling data and combining results of studies for each network meta-analysis. This should include, but not be limited to:<br>● *Handling of multi-arm trials;*<br>● *Selection of variance structure;*<br>● *Selection of prior distributions in Bayesian analyses; and*<br>● *Assessment of model fit.*	Methods
Assessment of Inconsistency	S2	Describe the statistical methods used to evaluate the agreement of direct and indirect evidence in the treatment network(s) studied. Describe efforts taken to address its presence when found.	Methods
Risk of bias across studies	15	Specify any assessment of risk of bias that may affect the cumulative evidence (e.g., publication bias, selective reporting within studies).	Methods

Additional analyses	16	Describe methods of additional analyses if done, indicating which were pre-specified. This may include, but not be limited to, the following:<br>● Sensitivity or subgroup analyses;<br>● Meta-regression analyses;<br>● *Alternative formulations of the treatment network; and*<br>● *Use of alternative prior distributions for Bayesian analyses (if applicable).*	Methods
**RESULTS†**			
Study selection	17	Give numbers of studies screened, assessed for eligibility, and included in the review, with reasons for exclusions at each stage, ideally with a flow diagram.	Results, Fig. 1
Presentation of network structure	S3	Provide a network graph of the included studies to enable visualization of the geometry of the treatment network.	Supplement, Fig. 2
Summary of network geometry	S4	Provide a brief overview of characteristics of the treatment network. This may include commentary on the abundance of trials and randomized patients for the different interventions and pairwise comparisons in the network, gaps of evidence in the treatment network, and potential biases reflected by the network structure.	Supplement, Fig, 2
Study characteristics	18	For each study, present characteristics for which data were extracted (e.g., study size, PICOS, follow-up period) and provide the citations.	Supplement
Risk of bias within studies	19	Present data on risk of bias of each study and, if available, any outcome level assessment.	Supplement, Results
Results of individual studies	20	For all outcomes considered (benefits or harms), present, for each study: 1) simple summary data for each intervention group, and 2) effect estimates and confidence intervals. *Modified approaches may be needed to deal with information from larger networks.*	Supplement,
Synthesis of results	21	Present results of each meta-analysis done, including confidence/credible intervals. *In larger networks, authors may focus on comparisons versus a particular comparator (e.g. placebo or standard care), with full findings presented in an appendix. League tables and forest plots may be considered to summarize pairwise comparisons.* If additional summary measures were explored (such as treatment rankings), these should also be presented.	Results, Supplement, Fig. 3
Exploration for inconsistency	S5	Describe results from investigations of inconsistency. This may include such information as measures of model fit to compare consistency and inconsistency models, *P* values from statistical tests, or summary of inconsistency estimates from different parts of the treatment network.	Supplement, Results
Risk of bias across studies	22	Present results of any assessment of risk of bias across studies for the evidence base being studied.	Supplement, Results
Results of additional analyses	23	Give results of additional analyses, if done (e.g., sensitivity or subgroup analyses, meta-regression analyses, *alternative network geometries studied, alternative choice of prior distributions for Bayesian analyses,* and so forth).	Results
DISCUSSION			
Summary of evidence	24	Summarize the main findings, including the strength of evidence for each main outcome; consider their relevance to key groups (e.g., healthcare providers, users, and policy-makers).	Discussion
Limitations	25	Discuss limitations at study and outcome level (e.g., risk of bias), and at review level (e.g., incomplete retrieval of identified research, reporting bias). *Comment on the validity of the assumptions, such as transitivity and consistency. Comment on any concerns regarding network geometry (e.g., avoidance of certain comparisons).*	Discussion
Conclusions	26	Provide a general interpretation of the results in the context of other evidence, and implications for future research.	Conclusions
FUNDING			
Funding	27	Describe sources of funding for the systematic review and other support (e.g., supply of data); role of funders for the systematic review. This should also include information regarding whether funding has been received from manufacturers of treatments in the network and/or whether some of the authors are content experts with professional conflicts of interest that could affect use of treatments in the network.	p.12

5 Table S2. Risk of bias explanation.

Adapted QUADAS-2 questions

Domain 1: Patient selection
Risk of bias: could the selection of patients have introduced bias?
Was a consecutive or random sample of patients enrolled?
Was a case–control design avoided?
Did the study avoid inappropriate exclusions?
A clear objective and description of inclusion criteria of the study population?
A detailed description of the study population?

Domain 2: Index test
Risk of Bias: Could the conduct or interpretation of the index test have introduced bias?
Was appropriate cross-validation or external validation and evaluation method used?

Domain 3: Reference standard
Risk of Bias: Could the reference standard, its conduct, or its interpretation have introduced bias?
Is the reference standard likely to correctly classify the target condition?

Domain 4: Flow and timing
Risk of Bias: Could the Patient Flow Have Introduced Bias?
Did all patients receive the same reference standard?
Were all patients included in the analysis?

Domain 5: Data management
Could data management have introduced bias?
A clear description of the data source and how data was collected
A valid and reproducible data collection and measurement method

We employed an average bias method to calculate the overall risk of bias. For each study, a sum score was computed, considering the following criteria: ☺ was assigned a score of 0 and ☹ a score of 1. We implemented a rule that the overall risk of bias considered ‘Low’ of if sum score = 0, ‘Moderate’ if sum score = 1 and ‘High’ of sum score 2 and more.

6 Table S3. Major exclusions with reason for exclusion.

Cause of exclusion	Study
Preprint	arXiv:1711.11536
	arXiv:2107.11094v1
	https://doi.org/10.1101/2022.03.15.22271655
	https://doi.org/10.1101/2022.04.29.22274361
	https://doi.org/10.21203/rs.3.rs-697902/v1
	arXiv:2107.05230v1
	arXiv:1812.06686v2
	arXiv:2205.15492v1
	arXiv:2209.00439v1
Conference paper	https://doi.org/10.1145/2975167.2985841
	https://doi.org/10.1109/ichi.2018.00068
	https://doi.org/10.1109/sieds.2015.7116970
	Futoma, Joseph D., Sanjay Hariharan, Katherine A. Heller, Mark P. Sendak, Nathan Brajer, Meredith Edwards Clement, Armando D Bedoya and Cara O'Brien. "An Improved Multi-Output Gaussian Process RNN with Real-Time Validation for Early Sepsis Detection." Machine Learning in Health Care (2017).
	https://doi.org/10.1109/confluence51648.2021.9377090
	https://doi.org/10.1109/confluence51648.2021.
	https://doi.org/10.22489/CinC.2019.423
No sepsis definition criteria	Brown, S.M., Jones, J., Kuttler, K.G. et al. Prospective evaluation of an automated method to identify patients with severe sepsis or septic shock in the emergency department. BMC Emerg Med 16, 31 (2016). https://doi.org/10.1186/s12873-016-0095-0
	Kaya, U.; Yılmaz, A.; Aşar, S. Sepsis Prediction by Using a Hybrid Metaheuristic Algorithm: A Novel Approach for Optimizing Deep Neural Networks. *Diagnostics* 2023, *13*, 2023. https://doi.org/10.3390/diagnostics13122023
Mortality prediction (not sepsis development)	Cheng CY, Kung CT, Chen FC, Chiu IM, Lin CR, Chu CC, Kung CF, Su CM. Machine learning models for predicting in-hospital mortality in patient with sepsis: Analysis of vital sign dynamics. Front Med (Lausanne). 2022 Oct 20;9:964667. doi: 10.3389/fmed.2022.964667. PMID: 36341257; PMCID: PMC9631306.
	Li S, Dou R, Song X, Lui KY, Xu J, Guo Z, Hu X, Guan X, Cai C. Developing an Interpretable Machine Learning Model to Predict in-Hospital Mortality in Sepsis Patients: A Retrospective Temporal Validation Study. J Clin Med. 2023 Jan 24;12(3):915. doi: 10.3390/jcm12030915. PMID: 36769564; PMCID: PMC9917524.
No relevant outcomes	Moss, T. J., Lake, D. E., Calland, J. F., Enfield, K. B., Delos, J. B., Fairchild, K. D., & Moorman, J. R. (2016). Signatures of Subacute Potentially Catastrophic Illness in the ICU. Critical Care Medicine, 44(9), 1639–1648. doi:10.1097/ccm.0000000000001738
	Strickler, E.A.T., Thomas, J., Thomas, J.P. et al. Exploring a global interpretation mechanism for deep learning networks when predicting sepsis. Sci Rep 13, 3067 (2023). https://doi.org/10.1038/s41598-023-30091-3
Partially pediatric cohort	Alanazi A, Aldakhil L, Aldhoayan M, Aldosari B. Machine Learning for Early Prediction of Sepsis in Intensive Care Unit (ICU) Patients. Medicina (Kaunas). 2023 Jul 9;59(7):1276. doi: 10.3390/medicina59071276. PMID: 37512087; PMCID: PMC10385427.
No data on patient cohort	https://www.irjet.net/archives/V10/i5/IRJET-V10I5314.pdf

Table S4. Baseline characteristics of retrospective diagnostic test accuracy studies.

№	Study	Year	Data collection period	Sepsis patients/all patients	Sepsis prevalence, %	Age, mean (y)	Sex, male (%)	Hospital mortality (%)
1	Abromavicius, 2020 [27]	2020	2009 to 2019	2932/40336	7.27	62.8	55.9	ND
2	Amrollahi, 2020 [28]	2020	2001 to 2012	2805/40175	6.98	63.3	57.5	ND
3	Aşurŏglu, 2020 [39]	2020	2001 to 2012	1404/5154	27.24	ND	ND	ND
4	Bao, 2022 [50]	2022	2012 to 2019	21680/ND	ND	67.0	55.0	17.4
5	Barton, 2019 [60]	2019	2001 to 2016	3673/112952	3.25	56.9	50.1	6.9
6	Bedoya, 2020 [71]	2020	2014 to 2018	8160/42979	18.99	56.0	48.0	5.0
7	Bloch, 2019 [82]	2019	2007 to 2014	300/600	50.00	54.0	62.5	ND
8	Burdick, 2020 [93]	2020	2001 to 2017	20876/245257	8.51	54.2	43.7	ND
9	Calvert, 2016 [97]	2016	2001 to 2008	159/1394	11.41	63.3	56.3	11.7
10	Camacho-Cogollo, 2022 [98]	2022	2001 to 2012	537/2377	22.59	62.4	ND	6.9
11	Chen, 2021 [29]	2021	2009 to 2019	2932/40336	7.27	62.8	55.9	ND
12	Chen, 2022 [30]	2022	2001 to 2021	1229/7411	16.58	61.4	58.0	ND
13	Chen, 2023 [31]	2023	2015 to 2021	216/677	31.90	49.0	86.1	ND
14	Choi, 2020 [32]	2020	2014 to 2017	1136/7743	14.67	65.0	ND	ND
15	Delahanty, 2019 [33]	2019	2016 to 2017	54661/2759529	1.98	47.8	42.3	0.6
16	Desautels, 2016 [34]	2016	2001 to 2012	2577/22583	11.41	65.0	56.15	6.9
17	Duan, 2023 [35]	2023	2016 to 2021	114/282	40.40	ND	52.0	ND
18	El-Rashidy, 2022 [36]	2022	2001 to 2012	2680/4000	67.00	35.0	ND	ND
19	Fagerström, 2019 [37]	2019	2001 to 2012	11224/50373	22.28	65.0	56.2	3.7
20	Faisal, 2018 [38]	2018	2014 to 2015	12634/30996	40.76	67.0	49.9	5.7
21	Gholamzadeh, 2023 [40]	2023	2001 to 2012	24270/685110	3.54	ND	ND	ND
22	Giannini, 2019 [41]	2019	2011 to 2014	950/162212	0.59	57.9	49.6	1.9
23	Goh, 2021 [42]	2021	2015 to 2017	637/41792	1.52	ND	ND	ND
24	Horng, 2017 [43]	2017	2008 to 2013	32103/230936	13.90	50.4	45.3	1.4
25	Ibrahim, 2019 [44]	2019	2001 to 2012	4256/13728	31.00	ND	ND	ND
26	Kaji, 2019 [45]	2019	2001 to 2012	36176/56841	63.64	ND	ND	ND
27	Kam, 2017 [46]	2017	2001 to 2012	360/5789	6.22	62.5	54.2	10.3
28	Khojandi, 2018 [47]	2018	2008 to 2015	29343/261258	11.23	63.56	47.3	ND
29	Kijpaisalratana, 2022 [48]	2022	2018 to 2020	1207/133707	0.90	49.0	42.8	1.2
30	Kuo, 2021 [49]	2021	2009 to 2019	2932/40336	7.27	62.8	55.9	ND
31	Kwon, 2021 [51]	2021	2016 to 2020	1548/46017	3.36	58.1	47.0	ND
32	Lauritsen 1, 2020 [52]	2020	2010 to 2017	ND/3126	ND	55.2	47.6	0.8
33	Lauritsen 2, 2021 [14]	2021	2012 to 2021	1250/19976	6.26	70.0	48.0	ND
34	Li, 2020 [53]	2020	2009 to 2019	2932/40336	7.27	62.8	55.9	ND
35	Lin, 2021 [54]	2021	2016, 2018	2248/10040	22.39	ND	ND	ND
36	Liu, 2022 [55]	2022	ND	3559/7283	48.87	60.0	59.0	14.6
37	Liu, 2023 [56]	2023	2001 to 2019	261/1338	19.51	58.0	57.0	ND
38	Maharjan, 2022 [57]	2022	2018 to 2021	7600/95748	7.94	ND	ND	ND

39	Mao, 2017 [58]	2017	2011 to 2016	1592/111957	1.42	55.6	45.0	2.3
40	McCoy, 2017 [59]	2017	2016 to 2017	1328/1665	79.80	ND	48.0	2.93
41	Moor, 2023 [61]	2023	2001 to 2016	25694/136478	18.80	65.0	57.0	6.5
42	Nemati, 2018 [62]	2018	2013 to 2015	2375/27527	8.63	61.0	52.7	9.3
43	Nesaragi, 2021 [63]	2021	2009 to 2019	2932/40336	7.27	62.8	55.9	ND
44	Oei, 2021 [64]	2021	2001 to 2012	13935/48632	28.65	64.3	56.0	ND
45	Persson, 2021 [65]	2021	2001 to 2012	405/2893	14.00	64.2	58.2	15.4
46	Rafiei, 2021 [66]	2021	2009 to 2019	2932/40336	7.27	62.8	55.9	ND
47	Rangan, 2022 [67]	2022	2009 to 2019	1825/10500	17.38	61.0	53.0	ND
48	Rosnati, 2021 [68]	2021	2001 to 2012	7936/22007	36.06	ND	ND	ND
49	Sadasivuni, 2022 [69]	2022	2014 to 2018	514/965	53.26	ND	51.3	ND
50	Scherpf, 2019 [72]	2019	2001 to 2012	1172/31238	3.75	ND	ND	ND
51	Shamoni, 2019 [73]	2019	2016 to 2017	200/620	32.26	61.1	56.6	ND
52	Sharma, 2021 [70]	2021	2009 to 2019	2932/40336	7.27	62.8	55.9	ND
53	Shashikumar 1, 2017 [74]	2017	ND	242/1100	22.00	59.0	53.0	15.2
54	Shashikumar 2, 2017 [75]	2017	ND	150/250	60.00	61.0	51.0	15.2
55	Shashikumar 3, 2021 [76]	2021	ND	1445/25820	5.60	61.0	53.3	ND
56	Shimabukuro, 2017 [78]	2017	2016 to 2017	67/142	47.18	59.0	46.5	15.5
57	Singh, 2022 [77]	2022	2011 to 2012	1572/X	ND	67.3	55.6	ND
58	Taneja, 2017 [79]	2017	2014 to 2016	76/444	17.12	61.0	54.5	ND
59	Tang, 2021 [80]	2021	2019 to 2020	1376/2453	56.09	55.7	51.2	ND
60	Valik, 2023 [81]	2023	2012 to 2013	8038/55655	9.70	63.0	47.3	2.3
61	Van Wyk, 2018 [83]	2018	2017	377/904	41.70	ND	ND	ND
62	Wang 1, 2021 [84]	2021	2014 to 2016	3539/4449	79.55	58.0	60.2	ND
63	Wang 2, 2021 [85]	2021	2009 to 2019	2932/40336	7.27	62.8	55.9	ND
64	Wong, 2021 [86]	2021	2018 to 2019	2552/27697	9.21	56.0	43.0	ND
65	Yang 1, 2020 [87]	2020	2009 to 2019	2932/40336	7.27	62.8	55.9	ND
66	Yang 2, 2022 [88]	2022	2014 to 2019	455/1383	32.90	58.9	58.9	ND
67	Yu, 2022 [89]	2022	2012 to 2019	2206/70034	3.15	61.0	53.2	8.3
68	Yuan, 2020 [90]	2020	2018	444/1588	27.96	70.1	59.8	ND
69	Zargoush, 2021 [91]	2021	2009 to 2019	2932/40336	7.27	62.8	55.9	ND
70	Zhang, 2021 [92]	2021	2000 to 2016	52802/178843	29.52	ND	48.9	ND
71	Zhang 2, 2023 [95]	2023	2008 to 2019	550/1118	49.19	ND	ND	ND
72	Zhang 3, 2023 [94]	2023	2001 to 2021	2198/4853	45.30	ND	59.0	16.8
73	Zhao, 2021 [96]	2021	2009 to 2019	1714/22336	7.67	62.8	55.9	ND

Abbreviations. ND, no data

Table S5. Characteristics of machine learning models for sepsis prediction.

№	Study	Prediction models	Sepsis definition criteria	Department	Prediction window, h.	External validation	Imputation	Features				Total, N
								BT	Lab.	HR	RR	
1	Abromavicius, 2020 [27]	DT, NB, SVM	Sepsis-3	ICU	0 to 6	No	No	Yes	Yes	Yes	Yes	39
2	Amrollahi, 2020 [28]	LSTM	Sepsis-3	ICU	4	No	No	Yes	No	Yes	Yes	10
3	Aşurŏglu, 2020 [39]	CNN, RF, KNN, NB, SVM, Deep DSPA, CNN-RF hybrid	SOFA score	ICU	6	No	Yes	Yes	No	Yes	Yes	76
4	Bao, 2022 [50]	SVM, DT, RF, GBM, NNM, XGB, LGBM	Sepsis-3	ICU	0	No	Yes	Yes	Yes	No	Yes	15
5	Barton, 2019 [60]	XGB	Sepsis-3	ED	0 to 48	No	Yes	Yes	No	Yes	Yes	6
6	Bedoya, 2020 [71]	NNM, Cox-LR, RF, log, LR.	SIRS, a blood culture order, and at least one element of end-organ failure	ED	0 to 12	No	Yes	Yes	Yes	Yes	Yes	86
7	Bloch, 2019 [82]	NNM, SVM, LR	Sepsis-2 related	ICU	4	No	No	Yes	No	Yes	Yes	4
8	Burdick, 2020 [93]	XGB tree	Sepsis-2, ICD-9	In-Hospital, ED	0 to 48	Yes	Yes	Yes	Yes	Yes	Yes	15
9	Calvert, 2016 [97]	InSight Algorithm (GLM)	SIRS criteria, ICD-9	ICU	3	No	No	Yes	Yes	Yes	Yes	9
10	Camacho-Cogollo, 2022 [98]	SVM, KNN, NNM, RF, AdaBoost, XGB	Sepsis-3	ICU	1 to 3	No	Yes	Yes	Yes	Yes	Yes	31
11	Chen, 2021 [29]	RF	Sepsis-3	ICU	0 to 6	No	Yes	Yes	Yes	Yes	Yes	20
12	Chen, 2022 [30]	LightGBM, MLP	Sepsis-3	ICU	0 to 5	Yes	Yes	Yes	Yes	Yes	Yes	78
13	Chen, 2023 [31]	LR, SVM, RF, GBM, ADABoost, NB, NNM	Sepsis-3	ICU	168	Yes	Yes	No	Yes	No	No	59
14	Choi, 2020 [32]	LR	ICD-10	In-Hospital	0	No	Yes	No	Yes	No	No	36
15	Delahanty, 2019 [33]	GBT	Sepsis-3	ED	1 to 24	No	No	Yes	Yes	No	Yes	12
16	Desautels, 2016 [34]	InSight Algorithm (GLM)	Sepsis-3	ICU	0, 4	No	Yes	Yes	No	Yes	Yes	9
17	Duan, 2023 [35]	NNM, XGB	Sepsis-2	ICU	6, 12, 24	No	Yes	Yes	Yes	Yes	Yes	30
18	El-Rashidy, 2022 [36]	RF, XGB, NNM	SIRS, ICD-9	ICU	2+	No	Yes	Yes	Yes	Yes	Yes	660
19	Fagerström, 2019 [37]	LSTM	SIRS, ICD-9	ICU	0 to 44	No	Yes	No	Yes	Yes	Yes	24
20	Faisal, 2018 [38]	LR	ICD-10	ICU	4	Yes	Yes	Yes	Yes	Yes	Yes	22
21	Gholamzadeh, 2023 [40]	LR, NB, DT, RF, XGB, KNN	Sepsis-3	ICU	0	No	Yes	Yes	Yes	Yes	Yes	31
22	Giannini, 2019 [41]	RF	ICD-9 code	In-Hospital	48	No	No	Yes	Yes	Yes	Yes	64
23	Goh, 2021 [42]	LR, RF	ICD-10	ICU	4 to 48	No	No	Yes	Yes	Yes	Yes	15

24	Horng, 2017 [43]	SVM, LR, NB, RFs	ICD-9 code	ED	4	No	Yes	Yes	No	Yes	Yes	10
25	Ibrahim, 2019 [44]	RF, GB, SVM	Sepsis-3, ICD-9	ICU	0	No	Yes	Yes	Yes	Yes	Yes	63
26	Kaji, 2019 [45]	LSTM	Sepsis-2 related	ICU	24	No	Yes	Yes	Yes	Yes	Yes	119
27	Kam, 2017 [46]	NNM	SIRS criteria, ICD-9	ICU	0 to 3	No	No	Yes	Yes	Yes	Yes	9
28	Khojandi, 2018 [47]	RF	SIRS	In-Hospital	0	No	No	Yes	Yes	Yes	Yes	9
29	Kijpaisalratana, 2022 [48]	LR, GBT, RF, NNM	ICD-10	ED	0	No	Yes	Yes	No	Yes	No	13
30	Kuo, 2021 [49]	NNM	Sepsis-3	ICU	0 to 36	No	Yes	Yes	Yes	Yes	Yes	43
31	Kwon, 2021 [51]	NNM	Sepsis-3	In-Hospital	0	Yes	No	No	No	Yes	No	10
32	Lauritsen 1, 2020 [52]	CNN-NNM	Sepsis-2 related	ED	3	No	No	Yes	Yes	Yes	Yes	ND
33	Lauritsen 2, 2021 [14]	Extra trees, RF, LGB, XGB, LR	Sepsis-3	In-hospital	12	No	Yes	Yes	Yes	Yes	Yes	25
34	Li, 2020 [53]	LightGBM	Sepsis-3	ICU	0 to 6	No	No	Yes	Yes	Yes	No	40
35	Lin, 2021 [54]	XGB tree	Sepsis-3	ED	0	Yes	No	Yes	Yes	Yes	Yes	32
36	Liu, 2022 [55]	RF, NNM	Sepsis-3	ICU	1 to 7, 1 to 11	No	No	ND	ND	ND	ND	87
37	Liu, 2023 [56]	SVM, NNM, GBT, AdaBoost, KNN, LR	Sepsis-3	ICU	0	No	Yes	Yes	Yes	Yes	Yes	13
38	Maharjan, 2022 [57]	XGB	ICD-10 + text or SOFA score	In-Hospital, ED	6	Yes	Yes	Yes	Yes	Yes	Yes	30
39	Mao, 2017 [58]	InSight Algorithm (GLM)	SIRS criteria, ICD-9	ED	0	Yes	Yes	Yes	No	Yes	Yes	30
40	McCoy, 2017 [59]	GLM	Sepsis-3	In-Hospital	0	No	Yes	Yes	Yes	Yes	Yes	6
41	Moor, 2023 [61]	NNM, LightGBM, LR	Sepsis-3	ICU	6	Yes	Yes	Yes	Yes	Yes	Yes	63
42	Nemati, 2018 [62]	Proportional hazards model	Sepsis-3	ICU	4 to 12	Yes	No	Yes	Yes	Yes	Yes	65
43	Nesaragi, 2021 [63]	LightGBM	Sepsis-3	ICU	0 to 6	No	Yes	Yes	Yes	Yes	Yes	85
44	Oei, 2021 [64]	DNN	ICD-9, SOFA	In-hospital	3 to 12	No	No	Yes	Yes	Yes	Yes	530
45	Persson, 2021 [65]	NNM	Sepsis-3	ICU	0 to 3	Yes	No	Yes	Yes	Yes	Yes	20
46	Rafiei, 2021 [66]	LSTM, NNM	Sepsis-3	ICU	4 to 8	No	Yes	Yes	Yes	Yes	Yes	14
47	Rangan, 2022 [67]	XGB	Sepsis-3	ICU	0 to 6	Yes	Yes	Yes	No	Yes	Yes	4
48	Rosnati, 2021 [68]	NNM, LR, InSight	Sepsis-3	ICU	0 to 6	No	Yes	Yes	Yes	Yes	Yes	24
49	Sadasivuni, 2022 [69]	RF, LR, NNM, SVM	Sepsis-3	ICU	1 to 6	No	No	ND	Yes	ND	ND	ND
50	Scherpf, 2019 [72]	NNM	SIRS	ICU	3 to 12	No	Yes	Yes	Yes	Yes	Yes	10
51	Shamoni, 2019 [73]	Non-linear Regression	Sepsis tag by ICU clinicians	ICU	4 to 24	No	Yes	Yes	Yes	Yes	Yes	55
52	Sharma, 2021 [70]	LSTM	Sepsis-3	ICU	0 to 6	No	No	Yes	No	Yes	Yes	10
53	Shashikumar 1, 2017 [74]	InSight Algorithm (GLM)	Sepsis-3	ICU	4	No	Yes	Yes	No	Yes	Yes	21
54	Shashikumar 2, 2017 [75]	SVM	Sepsis-3	ICU	4	No	No	Yes	Yes	Yes	Yes	22
55	Shashikumar 3, 2021 [76]	NNM, LR	Sepsis-3	ICU	2 to 12	Yes	Yes	Yes	Yes	Yes	Yes	65

56	Shimabukuro, 2017 [78]	GLM	SIRS	ICU	0	No	No	Yes	Yes	Yes	Yes	17
57	Singh, 2022 [77]	SVM, NB, RF, LR, XGB	Sepsis-2, 3	ICU	0	No	Yes	Yes	Yes	Yes	Yes	21
58	Taneja, 2017 [79]	LR, NB, SVM, RF, AdaBoost (другое)	Sepsis-3	In-Hospital	0 to 24	No	No	Yes	Yes	Yes	Yes	21
59	Tang, 2021 [80]	XGB, AdaBoost, LR	Viral Sepsis Caused by SARS-CoV-2 (VSCS-2): Sepsis-1	In-hospital	0	No	No	No	Yes	No	No	45
60	Valik, 2023 [81]	LightGBM	Sepsis-3	In-hospital	48	No	Yes	Yes	Yes	Yes	Yes	13
61	Van Wyk, 2018 [83]	RF	Sepsis-2 related	ICU	3	No	No	Yes	Yes	Yes	Yes	7
62	Wang 1, 2021 [84]	RF	Sepsis-3	ICU	0	Yes	No	No	Yes	No	No	55
63	Wang 2, 2021 [85]	LSTM, NNM	Sepsis-3	ICU	0 to 6	No	Yes	Yes	Yes	Yes	Yes	40
64	Wong, 2021 [86]	Epic Sepsis Model (ESM) - LR	CDC, ICD-10	In-hospital	4, 8, 12, 24	Yes	No	Yes	Yes	Yes	Yes	80
65	Yang 1, 2020 [87]	XGB	Sepsis-3	ICU	0 to 6	No	No	Yes	Yes	Yes	Yes	168
66	Yang 2, 2022 [88]	LR, RF, NNM, CNN, RNN	Sepsis-3	In-Hospital, ED, ICU	48	No	Yes	Yes	Yes	Yes	Yes	1738
67	Yu, 2022 [89]	XGB, LR	Sepsis-3	In-hospital	6	No	Yes	Yes	Yes	Yes	Yes	79
68	Yuan, 2020 [90]	XGB	Sepsis-3	ICU	8	No	No	Yes	Yes	Yes	Yes	106
69	Zargoush, 2021 [91]	LSTM, XGB, LR, ADA, NB, CART	Sepsis-3	ICU	0 to 26	No	Yes	Yes	Yes	Yes	Yes	25
70	Zhang, 2021 [92]	LSTM, LR, RF, GBT, GRU, RETAIN - NNM, Dipole	Sepsis-2	ED	4	No	No	Yes	Yes	Yes	Yes	113
71	Zhang 2, 2023 [95]	RF, XGB, SVM, ET	Sepsis-3	ICU	0	No	Yes	Yes	Yes	Yes	Yes	24
72	Zhang 3, 2023 [94]	NB, LR, NNM, DT, AdaBoost, SVM, KNN	ICD-9	ICU	3 to 6	Yes	Yes	Yes	Yes	Yes	Yes	17
73	Zhao, 2021 [96]	XGB, LightGBM	Sepsis-3	ICU	0 to 6	No	Yes	Yes	Yes	Yes	Yes	40

Abbreviations. ND, no data; BT, Body Temperature; HR, Heart rate; RR, Respiratory Rate; ICU, Intensive Care Unit; ED, Emergence Department; ICD, International Classification of Diseases; CDC, Centers for Disease Control and Prevention; biLSTM, Bidirectional Long Short-Term Memory; CART, Classification And Regression Trees; CNN, Convolutional Neural Network; DT, Decision Tree; DNN, Deep Neural Network; DSPA, Deep SOFA-Sepsis Prediction Algorithm; ESM, Epic Sepsis Model; ET, Extremely Randomized Trees; GB, Gradient Boosting; GBM, Gradient Boosting Machine; GLM, Generalized Linear Model; GRU, Gated Recurrent Unit; GBT, Gradient Boosted Trees; KNN, K-Nearest Neighbors; LGB, Light Gradient Boosting; LR, Logistic Regression; LSTM, Long Short-Term Memory; MLP, Multi-Layer Perceptron; NNM, Neural Network Model; NB, Naïve Bayes; RF, Random Forest; RNN, Recurrent Neural Network; SVM, Support Vector Machine; TCN, Temporal Convolutional Network; XGB, XGBoost.

9 **Table S6. Additional characteristics of included studies.**

№	Study	Journal	Data source
1	Abromavicius, 2020 [1]	Electronics	PhysioNet/Computing in Cardiology Challenge 2019
2	Amrollahi, 2020 [2]	AMIA Annu Symp Proc	MIMIC-III
3	Aşurŏglu, 2020 [3]	Comput Methods Programs Biomed	MIMIC-III
4	Bao, 2022 [4]	Med Intensiva	MIMIC-IV, eICU
5	Barton, 2019 [5]	Comput Biol Med	University of California, San Francisco and MIMIC-III
6	Bedoya, 2020 [6]	JAMIA Open	Quaternary academic hospital
7	Bloch, 2019 [7]	J Healthc Eng	Rabin Medical Center
8	Burdick, 2020 [8]	BMC Med Inform Decis Mak	Dascena Analysis Dataset, Cabell Huntington Hospital Dataset
9	Calvert, 2016 [9]	Comput Biol Med	MIMIC-II
10	Camacho-Cogollo, 2022 [10]	Electronics	MIMIC-III
11	Chen, 2021 [11]	IRBM	PhysioNet/Computing in Cardiology Challenge 2019
12	Chen, 2022 [12]	BMC Med Inform Decis Mak	MIMIC-III, HDRJH, Ruijin real-world data
13	Chen, 2023 [13]	Ann Med	Third Affiliated Hospital of Sun Yatsen University
14	Choi, 2020 [14]	Sci Rep	Yonsei University Severance hospital
15	Delahanty, 2019 [15]	Ann Emerg Med	49 urban community hospitals operated by Tenet Healthcare
16	Desautels, 2016 [16]	JMIR Med Inform	MIMIC-III
17	Duan, 2023 [17]	Appl Intell	Hospital in Shanghai
18	El-Rashidy, 2022 [18]	Neural Comput Appl	MIMIC-III
19	Fagerström, 2019 [19]	Sci Rep	MIMIC-III
20	Faisal, 2018 [20]	Crit Care Med	York hospital, and Northern Lincolnshire and Google Hospital
21	Gholamzadeh, 2023 [21]	Inform Med Unlocked	MIMIC-III
22	Giannini, 2019 [22]	Crit Care Med	Penn Data Store
23	Goh, 2021 [23]	Nat Commun	Singapore government-based hospital
24	Horng, 2017 [24]	PLoS One	MIMIC-III
25	Ibrahim, 2019 [25]	J Am Med Inform Assoc	MIMIC-III
26	Kaji, 2019 [26]	PLoS One	MIMIC-III
27	Kam, 2017 [27]	Comput Biol Med	MIMIC-II
28	Khojandi, 2018 [28]	Methods Inf Med	Health Facts® (HF) datase
29	Kijpaisalratana, 2022 [29]	Int J Med Inform	Chulalongkorn University-affiliated hospital
30	Kuo, 2021 [30]	BMC Med Inform Decis Mak	PhysioNet/Computing in Cardiology Challenge 2019
31	Kwon, 2021 [31]	Scand J Trauma Resusc Emerg Med	Sejong General Hospital and Mediplex Sejong Hospital
32	Lauritsen 1, 2020 [32]	Artif Intell Med	Danish HER
33	Lauritsen 2, 2021 [33]	NPJ Digit Med	ROSS-TRACKS cohort
34	Li, 2020 [34]	Crit Care Med	PhysioNet/Computing in Cardiology Challenge 2019
35	Lin, 2021 [35]	J Pers Med	Chi-Mei Medical Center, Taoyuan General Hospital
36	Liu, 2022 [36]	INFORMS J Comput	Cerner CareAware iBus platform, eICU Collaborative Research Database
37	Liu, 2023 [37]	BMC Surg	MIMIC-III, MIMIC-IV

38	Maharjan, 2022 [38]	Soc Sci Res Netw	Six medical sites in USA
39	Mao, 2017 [39]	BMJ Open	University of California, San Francisco and MIMIC-III
40	McCoy, 2017 [40]	BMJ Open Qual	CRMC’s EHR systems
41	Moor, 2023 [41]	EClinicalMedicine	MIMIC-III, eICU, HiRID, AUMC
42	Nemati, 2018 [42]	Crit Care Med	Emory University Hospitals And MIMIC-III
43	Nesaragi, 2021 [43]	Infect Dis Sepsis	PhysioNet/Computing in Cardiology Challenge 2019
44	Oei, 2021 [44]	Intell Based Med	MIMIC-III
45	Persson, 2021 [45]	JMIR Form Res	MIMIC-III
46	Rafiei, 2021 [46]	Comput Biol Med	PhysioNet/Computing in Cardiology Challenge 2019
47	Rangan, 2022 [47]	JAMIA Open	PhysioNet/Computing in Cardiology Challenge 2019
48	Rosnati, 2021 [48]	PLoS One	MIMIC-III
49	Sadasivuni, 2022 [49]	Sci Rep	Emory University Hospital
50	Scherpf, 2019 [50]	Comput Biol Med	MIMIC-III
51	Shamoni, 2019 [51]	Artif Intell Med	Univercity Medical Centre Mannheim
52	Sharma, 2021 [52]	Expert Syst	PhysioNet/Computing in Cardiology Challenge 2019
53	Shashikumar 1, 2017 [53]	J Electrocardiol	BedMaster system
54	Shashikumar 2, 2017 [54]	Physiol Meas	BedMaster system
55	Shashikumar 3, 2021 [55]	Artif Intell Med	Emory, UCSD cohort (UC San Diego Health system), MIMIC-III (Results for Emory)
56	Shimabukuro, 2017 [56]	BMJ Open Respir Res	UCSF Medical Center at Parnassus Heights
57	Singh, 2022 [57]	J Healthc Eng	Skaraborg Hospital
58	Taneja, 2017 [58]	Sci Rep	Carle Foundation Hospital
59	Tang, 2021 [59]	Front Cell Infect Microbiol	Tongji Hospital
60	Valik, 2023 [60]	Sci Rep	Karolinska University Hospital, Sweden
61	Van Wyk, 2018 [61]	Int J Med Inform	MLH System
62	Wang 1, 2021 [62]	Front Public Health	First Affiliated Hospital of Zhengzhou University
63	Wang 2, 2021 [63]	IEEE J Biomed Health Inform	PhysioNet/Computing in Cardiology Challenge 2019
64	Wong, 2021 [64]	JAMA Intern Med	Michigan Medicine (the health system of the University of Michigan, Ann Arbor)
65	Yang 1, 2020 [65]	Crit Care Med	PhysioNet/Computing in Cardiology Challenge 2019
66	Yang 2, 2022 [66]	JMIR Med Inform	Samsung Medical Center
67	Yu, 2022 [67]	Front Digit Health	Barnes-Jewish Hospital / Washington University School of Medicine
68	Yuan, 2020 [68]	Int J Med Inform	Taipei Medical University Hospital
69	Zargoush, 2021 [69]	Sci Rep	PhysioNet/Computing in Cardiology Challenge 2019
70	Zhang, 2021 [70]	Patterns	Cerner Health Facts database
71	Zhang 2, 2023 [71]	Eur Rev Med Pharmacol Sci	MIMIC-IV
72	Zhang 3, 2023 [72]	Appl Intell	MIMIC-III, hospital dataset
73	Zhao, 2021 [73]	Comput Intell Neurosci	PhysioNet/Computing in Cardiology Challenge 2019

Table S7. Summary of outcome data in included studies (pairwise meta-analysis).

№	Study	Model type*	Prediction window, h.	ML AUC±SD	SOFA AUC±SD	qSOFA AUC±SD	NEWS/NEWS2 AUC±SD	MEWS AUC±SD	SIRS AUC±SD	SAPS II AUC±SD
1_1	Abromavicius, 2020	1	3	0.497±0.020						
1_2	Abromavicius, 2020	5	3	0.241±0.020						
1_3	Abromavicius, 2020	3	3	0.392±0.020						
2	Amrollahi, 2020	2	4	0.840±0.020						
3_1	Aşurŏglu, 2020	7	6	0.846±0.020						
3_2	Aşurŏglu, 2020	1	6	0.964±0.020						
3_3	Aşurŏglu, 2020	4	6	0.866±0.020						
3_4	Aşurŏglu, 2020	5	6	0.788±0.020						
3_5	Aşurŏglu, 2020	3	6	0.502±0.020						
3_6	Aşurŏglu, 2020	2	6	0.964±0.020						
4_1	Bao, 2022	3	0	0.750±0.000						
4_2	Bao, 2022	1	0	0.850±0.100						
4_3	Bao, 2022	2	0	0.820±0.110						
5_1	Barton, 2019	1	0	0.880±0.010	0.720±0.010	0.60±0.010		0.610±0.010	0.660±0.010	
5_2	Barton, 2019	1	24	0.840±0.040						
5_3	Barton, 2019	1	48	0.830±0.040						
6_1	Bedoya, 2020	2	6	0.943±0.000		0.578±0.006	0.690±0.006		0.748±0.005	
6_2	Bedoya, 2020	1	6	0.905±0.000						
6_3	Bedoya, 2020	4	6	0.925±0.000						
7_1	Bloch, 2019	2	4	0.857±0.020						
7_2	Bloch, 2019	3	4	0.884±0.020						
7_3	Bloch, 2019	4	4	0.846±0.020						
8	Burdick, 2020	1	0	0.948±0.010	0.716±0.030			0.725±0.030	0.655±0.030	
8	Burdick, 2020	1	4	0.880±0.030						
8	Burdick, 2020	1	6	0.861±0.010						
8	Burdick, 2020	1	12	0.732±0.040						
8	Burdick, 2020	1	24	0.811±0.060						
8	Burdick, 2020	1	48	0.752±0.030						
9	Calvert, 2016	6	3	0.830±0.020						
10_1	Camacho-Cogollo, 2022	3	1	0.506±0.020	0.599±0.020	0.390±0.020				
10_2	Camacho-Cogollo, 2022	2	1	0.817±0.020						
10_3	Camacho-Cogollo, 2022	7	1	0.768±0.020						
10_4	Camacho-Cogollo, 2022	1	1	0.919±0.020						
10_5	Camacho-Cogollo, 2022	3	2	0.506±0.020						
10_6	Camacho-Cogollo, 2022	2	2	0.817±0.020						

№	Study	Model type*	Prediction window, h.	ML AUC±SD	SOFA AUC±SD	qSOFA AUC±SD	NEWS/NEWS2 AUC±SD	MEWS AUC±SD	SIRS AUC±SD	SAPS II AUC±SD
10_7	Camacho-Cogollo, 2022	7	2	0.768±0.020						
10_8	Camacho-Cogollo, 2022	1	2	0.916±0.020						
10_9	Camacho-Cogollo, 2022	3	3	0.506±0.020						
10_10	Camacho-Cogollo, 2022	2	3	0.817±0.020						
10_11	Camacho-Cogollo, 2022	7	3	0.768±0.020						
10_12	Camacho-Cogollo, 2022	1	3	0.911±0.020						
11	Chen, 2021	1	3	0.847±0.020						
12_1	Chen, 2022	1	2.5	0.930±0.020						
12_2	Chen, 2022	2	2.5	0.930±0.020						
13_1	Chen, 2023	4	168	0.726±0.040	0.637±0.036					
13_2	Chen, 2023	3	168	0.710±0.040						
13_3	Chen, 2023	1	168	0.755±0.050						
13_4	Chen, 2023	5	168	0.724±0.040						
13_5	Chen, 2023	2	168	0.718±0.040						
14	Choi, 2020	4	0	0.835±0.020	0.642±0.020				0.518±0.020	
15_1	Delahanty, 2019	1	1	0.930±0.080	0.780±0.080	0.620±0.080	0.690±0.080	0.620±0.080	0.750±0.080	
15_2	Delahanty, 2019	1	3	0.950±0.080	0.810±0.080	0.640±0.080	0.740±0.080	0.670±0.080	0.780±0.080	
15_3	Delahanty, 2019	1	6	0.960±0.080	0.830±0.080	0.660±0.080	0.770±0.080	0.710±0.080	0.790±0.080	
15_4	Delahanty, 2019	1	12	0.970±0.080	0.870±0.080	0.730±0.080	0.790±0.080	0.740±0.080	0.780±0.080	
15_5	Delahanty, 2019	1	24	0.970±0.080	0.900±0.080	0.800±0.080	0.840±0.080	0.790±0.080	0.770±0.080	
16_1	Desautels, 2016	6	0	0.880±0.010	0.730±0.010	0.770±0.010		0.800±0.010	0.610±0.010	0.700±0.010
16_2	Desautels, 2016	6	4	0.740±0.010						
17_1	Duan. 2023	2	6	0.920±0.020					0.670±0.020	
17_2	Duan. 2023	1	6	0.870±0.020					0.670±0.020	
17_3	Duan. 2023	2	12	0.890±0.020					0.650±0.020	
17_4	Duan. 2023	1	12	0.850±0.020					0.650±0.020	
17_5	Duan. 2023	2	24	0.890±0.020					0.630±0.020	
17_6	Duan. 2023	1	24	0.850±0.020					0.630±0.020	
18_1	El-Rashidy, 2022	2	2	0.906±0.020	0.780±0.020			0.670±0.020		
18_2	El-Rashidy, 2022	1	2	0.864±0.020						
19_1	Fagerström, 2019	2	0	0.940±0.100						
19_2	Fagerström, 2019	2	4	0.930±0.100						
19_3	Fagerström, 2019	2	8	0.920±0.100						
19_4	Fagerström, 2019	2	12	0.920±0.100						
20	Faisal, 2018	4	4	0.780±0.010						
21	Gholamzadeh, 2023	4	0	0.717±0.030						
21	Gholamzadeh, 2023	5	0	0.709±0.030						
21	Gholamzadeh, 2023	7	0	0.770±0.030						

№	Study	Model type*	Prediction window, h.	ML AUC±SD	SOFA AUC±SD	qSOFA AUC±SD	NEWS/NEWS2 AUC±SD	MEWS AUC±SD	SIRS AUC±SD	SAPS II AUC±SD
21	Gholamzadeh, 2023	1	0	0.918±0.030						
22	Giannini, 2019	1	48	0.890±0.030						
23_1	Goh, 2021	4	4	0.920±0.020						
23_2	Goh, 2021	4	6	0.850±0.020						
23_3	Goh, 2021	4	12	0.890±0.020						
23_4	Goh, 2021	1	4	0.940±0.020						
23_5	Goh, 2021	1	6	0.920±0.020						
23_6	Goh, 2021	1	12	0.790±0.020						
23_7	Goh, 2021	1	24	0.780±0.020						
23_8	Goh, 2021	1	48	0.770±0.020						
24_1	Horng, 2017	3	4	0.860±0.010						
24_2	Horng, 2017	4	4	0.860±0.010						
24_3	Horng, 2017	5	4	0.830±0.010						
24_4	Horng, 2017	1	4	0.870±0.010						
25_1	Ibrahim, 2019	1	0	0.960±0.020						
25_2	Ibrahim, 2019	3	0	0.940±0.020						
26	Kaji, 2019	2	24	0.876±0.020						
27_1	Kam, 2017	2	0	0.990±0.020						
27_2	Kam, 2017	2	1	0.960±0.020						
27_3	Kam, 2017	2	2	0.940±0.020						
27_4	Kam, 2017	2	3	0.930±0.020						
28	Khojandi, 2018	1	0	0.630±0.010						
29_1	Kijpaisalratana, 2022	1	0	0.931±0.010		0.635±0.012		0.688±0.014	0.814±0.010	
29_2	Kijpaisalratana, 2022	4	0	0.930±0.010						
29_3	Kijpaisalratana, 2022	2	0	0.926±0.010						
30_1	Kuo, 2021	2	0	0.821±0.000						
30_2	Kuo, 2021	2	6	0.779±0.000						
30_3	Kuo, 2021	2	12	0.759±0.010						
30_4	Kuo, 2021	2	24	0.791±0.000						
30_5	Kuo, 2021	2	36	0.807±0.010						
31	Kwon, 2021	2	0	0.863±0.010						
32	Lauritsen 1, 2020	2	3	0.786±0.020						
33_1	Lauritsen 2, 2021	1	12	0.906±0.010						
33_2	Lauritsen 2, 2021	4	12	0.752±0.010						
34	Li, 2020	1	3	0.845±0.020						
35	Lin, 2021	1	0	0.750±0.020		0.660±0.020			0.570±0.020	
36_1	Liu, 2022	1	6	0.850±0.010						
36_2	Liu, 2022	2	6	0.850±0.010						

№	Study	Model type*	Prediction window, h.	ML AUC±SD	SOFA AUC±SD	qSOFA AUC±SD	NEWS/NEWS2 AUC±SD	MEWS AUC±SD	SIRS AUC±SD	SAPS II AUC±SD
37_1	Liu, 2023	4	0	0.896±0.030	0.745±0.040	0.780±0.050			0.552±0.050	0.625±0.000
37_2	Liu, 2023	1	0	0.985±0.010						
37_3	Liu, 2023	3	0	0.924±0.020						
37_4	Liu, 2023	2	0	0.916±0.020						
37_5	Liu, 2023	7	0	0.924±0.020						
38	Maharjan, 2022	1	6	0.880±0.010					0.690±0.010	
39	Mao, 2017	6	0	0.920±0.010	0.630±0.010			0.760±0.010	0.750±0.010	
40	McCoy, 2017	6	0	0.910±0.010	0.770±0.010	0.550±0.010		0.550±0.010	0.760±0.010	
41_1	Moor, 2023	2	6	0.751±0.000	0.761±0.001	0.542±0.001	0.650±0.001	0.568±0.001	0.609±0.001	
41_2	Moor, 2023	1	6	0.690±0.010						
41_3	Moor, 2023	4	6	0.656±0.000						
42_1	Nemati, 2018	4	4	0.850±0.020	0.870±0.018					
42_2	Nemati, 2018	4	6	0.850±0.020	0.850±0.018					
42_3	Nemati, 2018	4	8	0.840±0.020	0.850±0.018					
42_4	Nemati, 2018	4	12	0.830±0.020	0.820±0.018					
43	Nesaragi, 2021	1	3	0.859±0.010						
44_1	Oei, 2021	2	3	0.860±0.000						
44_2	Oei, 2021	2	6	0.830±0.000						
44_3	Oei, 2021	2	12	0.800±0.000						
45_1	Persson, 2021	2	0	0.850±0.020	0.368±0.018	0.628±0.018	0.675±0.018	0.668±0.018	0.643±0.018	
45_2	Persson, 2021	2	1	0.820±0.020	0.149±0.018	0.529±0.018	0.584±0.018	0.529±0.018	0.595±0.018	
45_3	Persson, 2021	2	2	0.820±0.020	0.183±0.018	0.576±0.018	0.601±0.018	0.617±0.018	0.607±0.018	
45_4	Persson, 2021	2	3	0.840±0.020	0.199±0.018	0.587±0.018	0.613±0.0178	0.644±0.018	0.627±0.017	
46_1	Rafiei, 2021	2	4	0.890±0.010						
46_2	Rafiei, 2021	2	8	0.920±0.010						
46_3	Rafiei, 2021	2	12	0.870±0.010						
47_2	Rangan, 2022	1	3	0.910±0.010		0.660±0.020	0.790±0.020		0.690±0.020	
47_3	Rangan, 2022	1	4	0.860±0.020		0.660±0.020	0.800±0.020		0.680±0.020	
47_4	Rangan, 2022	1	5	0.910±0.020		0.640±0.020	0.790±0.020		0.680±0.020	
47_5	Rangan, 2022	1	6	0.940±0.010		0.660±0.020	0.790±0.020		0.680±0.020	
48_1	Rosnati, 2021	4	0	0.508±0.020						
48_2	Rosnati, 2021	4	1	0.543±0.020						
48_3	Rosnati, 2021	4	2	0.538±0.020						
48_4	Rosnati, 2021	4	3	0.532±0.020						
48_5	Rosnati, 2021	4	4	0.538±0.020						
48_6	Rosnati, 2021	4	5	0.566±0.020						
48_7	Rosnati, 2021	4	6	0.572±0.020						
48_8	Rosnati, 2021	6	0	0.559±0.020						

№	Study	Model type*	Prediction window, h.	ML AUC±SD	SOFA AUC±SD	qSOFA AUC±SD	NEWS/NEWS2 AUC±SD	MEWS AUC±SD	SIRS AUC±SD	SAPS II AUC±SD
48_9	Rosnati, 2021	6	1	0.573±0.020						
48_10	Rosnati, 2021	6	2	0.552±0.020						
48_11	Rosnati, 2021	6	3	0.531±0.020						
48_12	Rosnati, 2021	6	4	0.559±0.020						
48_13	Rosnati, 2021	6	5	0.490±0.020						
48_14	Rosnati, 2021	6	6	0.547±0.020						
48_15	Rosnati, 2021	2	0	0.647±0.020						
48_16	Rosnati, 2021	2	1	0.664±0.020						
48_17	Rosnati, 2021	2	2	0.670±0.020						
48_18	Rosnati, 2021	2	3	0.665±0.020						
48_19	Rosnati, 2021	2	4	0.675±0.020						
48_20	Rosnati, 2021	2	5	0.664±0.020						
48_21	Rosnati, 2021	2	6	0.641±0.020						
49_1	Sadasivuni, 2022	1	1	0.920±0.020						
49_2	Sadasivuni, 2022	1	2	0.930±0.020						
49_3	Sadasivuni, 2022	1	3	0.915±0.020						
49_4	Sadasivuni, 2022	1	4	0.910±0.020						
49_5	Sadasivuni, 2022	1	5	0.920±0.020						
49_6	Sadasivuni, 2022	1	6	0.840±0.020						
49_7	Sadasivuni, 2022	3	1	0.920±0.020						
49_8	Sadasivuni, 2022	3	2	0.950±0.020						
49_9	Sadasivuni, 2022	3	3	0.930±0.020						
49_10	Sadasivuni, 2022	3	4	0.920±0.020						
49_11	Sadasivuni, 2022	3	5	0.920±0.020						
49_12	Sadasivuni, 2022	3	6	0.880±0.020						
49_13	Sadasivuni, 2022	4	1	0.920±0.020						
49_14	Sadasivuni, 2022	4	2	0.940±0.020						
49_15	Sadasivuni, 2022	4	3	0.920±0.020						
49_16	Sadasivuni, 2022	4	4	0.920±0.020						
49_17	Sadasivuni, 2022	4	5	0.920±0.020						
49_18	Sadasivuni, 2022	4	6	0.880±0.020						
49_19	Sadasivuni, 2022	2	1	0.920±0.020						
49_20	Sadasivuni, 2022	2	2	0.960±0.020						
49_21	Sadasivuni, 2022	2	3	0.920±0.020						
49_22	Sadasivuni, 2022	2	4	0.910±0.020						
49_23	Sadasivuni, 2022	2	5	0.900±0.020						
49_24	Sadasivuni, 2022	2	6	0.820±0.020						
50_1	Scherpf, 2019	2	3	0.810±0.010						

№	Study	Model type*	Prediction window, h.	ML AUC±SD	SOFA AUC±SD	qSOFA AUC±SD	NEWS/NEWS2 AUC±SD	MEWS AUC±SD	SIRS AUC±SD	SAPS II AUC±SD
50_2	Scherpf, 2019	2	6	0.790±0.010						
50_3	Scherpf, 2019	2	12	0.760±0.020						
51_1	Shamoni, 2019	4	4	0.837±0.010						
51_2	Shamoni, 2019	4	8	0.813±0.010						
51_3	Shamoni, 2019	4	12	0.815±0.020						
51_4	Shamoni, 2019	4	24	0.670±0.030						
52	Sharma, 2021	2	3	0.864±0.020						
53	Shashikumar 1, 2017	6	4	0.780±0.020						
54	Shashikumar 2, 2017	3	4	0.890±0.020						
55_1	Shashikumar 3, 2021	2	2	0.910±0.020						
55_2	Shashikumar 3, 2021	2	4	0.900±0.020						
55_3	Shashikumar 3, 2021	2	6	0.890±0.020						
55_4	Shashikumar 3, 2021	2	8	0.890±0.020						
55_5	Shashikumar 3, 2021	2	12	0.880±0.020						
55_6	Shashikumar 3, 2021	4	2	0.870±0.020						
55_7	Shashikumar 3, 2021	4	4	0.870±0.020						
55_8	Shashikumar 3, 2021	4	6	0.860±0.020						
55_9	Shashikumar 3, 2021	4	8	0.860±0.020						
55_10	Shashikumar 3, 2021	4	12	0.850±0.020						
56	Shimabukuro, 2017	6	0	0.952±0.000	0.756±0.001	0.518±0.001		0.524±0.001	0.681±0.001	
57_1	Singh, 2022	1	0	0.950±0.020						
57_2	Singh, 2022	4	0	0.760±0.020						
57_3	Singh, 2022	3	0	0.930±0.020						
57_4	Singh, 2022	5	0	0.740±0.020						
58_1	Taneja, 2017	4	24	0.790±0.020	0.540±0.020	0.400±0.020				
58_2	Taneja, 2017	3	24	0.810±0.020						
58_3	Taneja, 2017	1	24	0.810±0.020						
58_4	Taneja, 2017	5	24	0.800±0.020						
59_1	Tang, 2021	1	0	0.948±0.020						
59_2	Tang, 2021	4	0	0.838±0.020						
60	Valik, 2023	1	48	0.963±0.000		0.905±0.001				
61	Van Wyk, 2018	1	3	0.970±0.020						
62	Wang 1, 2021	1	0	0.910±0.040						
63	Wang 2, 2021	2	3	0.892±0.020						
64_1	Wong, 2021	4	4	0.760±0.000						
64_2	Wong, 2021	4	8	0.740±0.000						
64_3	Wong, 2021	4	12	0.730±0.000						
64_4	Wong, 2021	4	24	0.720±0.000						

№	Study	Model type*	Prediction window, h.	ML AUC±SD	SOFA AUC±SD	qSOFA AUC±SD	NEWS/NEWS2 AUC±SD	MEWS AUC±SD	SIRS AUC±SD	SAPS II AUC±SD
65	Yang 1, 2020	1	3	0.847±0.020						
66_1	Yang 2, 2022	4	48	0.720±0.020						
66_2	Yang 2, 2022	1	48	0.750±0.020						
66_3	Yang 2, 2022	2	48	0.730±0.020						
67_1	Yu, 2022	1	6	0.862±0.010		0.705±0.013	0.699±0.012		0.679±0.010	
67_2	Yu, 2022	4	6	0.857±0.010						
68	Yuan, 2020	1	8	0.890±0.020	0.596±0.020					
69_1	Zargoush, 2021	2	13	0.910±0.020						
69_2	Zargoush, 2021	1	13	0.780±0.020						
69_3	Zargoush, 2021	4	13	0.760±0.020						
69_4	Zargoush, 2021	5	13	0.680±0.020						
70_1	Zhang, 2021	2	4	0.860±0.020		0.590±0.020	0.620±0.020	0.630±0.020	0.620±0.020	
70_2	Zhang, 2021	4	4	0.840±0.020						
70_3	Zhang, 2021	1	4	0.860±0.020						
71_1	Zhang 2, 2023	1	0	0.760±0.010						
71_2	Zhang 2, 2023	3	0	0.650±0.010						
72_1	Zhang 3, 2023	5	3	0.950±0.020						
72_2	Zhang 3, 2023	4	3	0.940±0.020						
72_3	Zhang 3, 2023	1	3	0.940±0.020						
72_4	Zhang 3, 2023	2	3	0.940±0.020						
72_5	Zhang 3, 2023	3	3	0.940±0.020						
72_6	Zhang 3, 2023	7	3	0.910±0.020						
72_7	Zhang 3, 2023	5	6	0.890±0.020						
72_8	Zhang 3, 2023	4	6	0.880±0.020						
72_9	Zhang 3, 2023	1	6	0.890±0.020						
72_10	Zhang 3, 2023	2	6	0.880±0.020						
72_11	Zhang 3, 2023	3	6	0.880±0.020						
72_12	Zhang 3, 2023	7	6	0.880±0.020						
73_1	Zhao, 2021	1	2	0.974±0.020						
73_2	Zhao, 2021	1	3	0.966±0.020						
73_3	Zhao, 2021	1	6	0.979±0.020						

***1 = DT, 2 = NNM, 3 = SVM, 4 = LR, 5 = NB, 6 = GLM, 7 = KNN**

Abbreviations: DT, Decision Tree; NNM, Neural Network Model; SVM, Support Vector Machine; LR, Logistic Regression; NB, Naïve Bayes; GLM, Generalized Linear Model; KNN, K-Nearest Neighbors; AUC, Area Under the Curve; SD, Standard Deviation; ML, Machine Learning.

Outcome data are presented in AUC±SD

Table S8. Summary of outcome data in included studies (CINeMA network meta-analysis).

Study	№	Model type	AUC	SD	N	RoB	Indirectness
Abromavicius 2020	1	DT	0.497	0.020	40336	1	1
Abromavicius 2020	1	NB	0.241	0.020	40336	1	1
Abromavicius 2020	1	SVM	0.392	0.020	40336	1	1
Aşurŏglu 2020	2	KNN	0.846	0.020	5154	3	1
Aşurŏglu 2020	2	DT	0.964	0.020	5154	3	1
Aşurŏglu 2020	2	LR	0.866	0.020	5154	3	1
Aşurŏglu 2020	2	NB	0.788	0.020	5154	3	1
Aşurŏglu 2020	2	SVM	0.502	0.020	5154	3	1
Aşurŏglu 2020	2	NNM	0.964	0.020	5154	3	1
Bao 2022	3	SVM	0.750	0.010	21680	2	1
Bao 2022	3	DT	0.850	0.100	21680	2	1
Bao 2022	3	NNM	0.820	0.110	21680	2	1
Barton 2019	4	DT	0.880	0.010	112952	1	1
Barton 2019	4	ScoringSystem	0.720	0.010	112952	1	1
Bedoya 2020	5	NNM	0.943	0.010	42979	2	1
Bedoya 2020	5	DT	0.905	0.010	42979	2	1
Bedoya 2020	5	LR	0.925	0.010	42979	2	1
Bedoya 2020	5	ScoringSystem	0.748	0.010	42979	2	1
Bloch 2019	6	NNM	0.857	0.020	600	2	1
Bloch 2019	6	SVM	0.884	0.020	600	2	1
Bloch 2019	6	LR	0.846	0.020	600	2	1
Burdick 2020	7	DT	0.948	0.010	245257	3	1
Burdick 2020	7	ScoringSystem	0.725	0.030	245257	3	1
Camacho-Cogollo 2022 (1h)	8	SVM	0.506	0.020	2377	2	1
Camacho-Cogollo 2022 (1h)	8	NNM	0.817	0.020	2377	2	1
Camacho-Cogollo 2022 (1h)	8	KNN	0.768	0.020	2377	2	1
Camacho-Cogollo 2022 (1h)	8	DT	0.919	0.020	2377	2	1
Camacho-Cogollo 2022 (1h)	8	ScoringSystem	0.599	0.020	2377	2	1
Camacho-Cogollo 2022 (2h)	9	SVM	0.506	0.020	2377	2	1
Camacho-Cogollo 2022 (2h)	9	NNM	0.817	0.020	2377	2	1
Camacho-Cogollo 2022 (2h)	9	KNN	0.768	0.020	2377	2	1
Camacho-Cogollo 2022 (2h)	9	DT	0.916	0.020	2377	2	1
Camacho-Cogollo 2022 (3h)	10	SVM	0.506	0.020	2377	2	1
Camacho-Cogollo 2022 (3h)	10	NNM	0.817	0.020	2377	2	1
Camacho-Cogollo 2022 (3h)	10	KNN	0.768	0.020	2377	2	1
Camacho-Cogollo 2022 (3h)	10	DT	0.911	0.020	2377	2	1
Chen 2022	11	DT	0.930	0.020	7411	1	1
Chen 2022	11	NNM	0.930	0.020	7411	1	1
Chen 2023	12	LR	0.726	0.040	677	1	1
Chen 2023	12	SVM	0.710	0.040	677	1	1
Chen 2023	12	DT	0.755	0.050	677	1	1
Chen 2023	12	NB	0.724	0.040	677	1	1
Chen 2023	12	NNM	0.718	0.040	677	1	1
Chen 2023	12	ScoringSystem	0.637	0.040	677	1	1
Choi 2020	13	LR	0.835	0.020	7743	3	1
Choi 2020	13	ScoringSystem	0.518	0.020	7743	3	1
Delahanty 2019 (1h)	14	DT	0.930	0.080	2759529	1	1
Delahanty 2019 (1h)	14	ScoringSystem	0.780	0.080	2759529	1	1
Delahanty 2019 (3h)	15	DT	0.950	0.080	2759529	1	1
Delahanty 2019 (3h)	15	ScoringSystem	0.810	0.080	2759529	1	1
Delahanty 2019 (6h)	16	DT	0.960	0.080	2759529	1	1
Delahanty 2019 (6h)	16	ScoringSystem	0.830	0.080	2759529	1	1
Delahanty 2019 (12h)	17	DT	0.970	0.080	2759529	1	1
Delahanty 2019 (12h)	17	ScoringSystem	0.870	0.080	2759529	1	1
Delahanty 2019 (24h)	18	DT	0.970	0.080	2759529	1	1
Delahanty 2019 (24h)	18	ScoringSystem	0.900	0.080	2759529	1	1
Desautels 2016	19	GLM	0.880	0.010	22583	1	1
Desautels 2016	19	ScoringSystem	0.800	0.010	22583	1	1
Duan. 2023 (6h)	20	NNM	0.920	0.020	282	3	1
Duan. 2023 (6h)	20	DT	0.870	0.020	282	3	1
Duan. 2023 (6h)	20	ScoringSystem	0.670	0.020	282	3	1
Duan. 2023 (12h)	21	NNM	0.890	0.020	282	3	1

Duan. 2023 (12h)	21	DT	0.850	0.020	282	3	1
Duan. 2023 (12h)	21	ScoringSystem	0.650	0.020	282	3	1
Duan. 2023 (24h)	22	NNM	0.890	0.020	282	3	1
Duan. 2023 (24h)	22	DT	0.850	0.020	282	3	1
Duan. 2023 (24h)	22	ScoringSystem	0.630	0.020	282	3	1
El-Rashidy 2022	23	NNM	0.906	0.020	4000	3	1
El-Rashidy 2022	23	DT	0.864	0.020	4000	3	1
El-Rashidy 2022	23	ScoringSystem	0.780	0.020	4000	3	1
Gholamzadeh 2023	24	LR	0.717	0.030	685110	2	1
Gholamzadeh 2023	24	NB	0.709	0.030	685110	2	1
Gholamzadeh 2023	24	KNN	0.770	0.030	685110	2	1
Gholamzadeh 2023	24	DT	0.918	0.030	685110	2	1
Goh 2021 (4h)	25	LR	0.920	0.020	41792	3	1
Goh 2021 (4h)	25	DT	0.940	0.020	41792	3	1
Goh 2021 (6h)	26	LR	0.850	0.020	41792	3	1
Goh 2021 (6h)	26	DT	0.920	0.020	41792	3	1
Goh 2021 (12h)	27	LR	0.890	0.020	41792	3	1
Goh 2021 (12h)	27	DT	0.790	0.020	41792	3	1
Horng 2017	28	SVM	0.860	0.010	230936	2	1
Horng 2017	28	LR	0.860	0.010	230936	2	1
Horng 2017	28	NB	0.830	0.010	230936	2	1
Horng 2017	28	DT	0.870	0.010	230936	2	1
Ibrahim 2019	29	DT	0.960	0.020	13728	3	1
Ibrahim 2019	29	SVM	0.940	0.020	13728	3	1
Kijpaisalratana 2022	30	DT	0.931	0.010	133707	3	1
Kijpaisalratana 2022	30	LR	0.930	0.010	133707	3	1
Kijpaisalratana 2022	30	NNM	0.926	0.010	133707	3	1
Kijpaisalratana 2022	30	ScoringSystem	0.814	0.010	133707	3	1
Lauritsen 2 2021	31	DT	0.906	0.010	19976	1	1
Lauritsen 2 2021	31	LR	0.752	0.010	19976	1	1
Lin 2021	32	DT	0.750	0.020	10040	2	1
Lin 2021	32	ScoringSystem	0.660	0.020	10040	2	1
Liu 2022	33	DT	0.850	0.010	7283	2	1
Liu 2022	33	NNM	0.850	0.010	7283	2	1
Liu 2023	34	LR	0.896	0.030	1338	1	1
Liu 2023	34	DT	0.985	0.010	1338	1	1
Liu 2023	34	SVM	0.924	0.020	1338	1	1
Liu 2023	34	NNM	0.916	0.020	1338	1	1
Liu 2023	34	KNN	0.924	0.020	1338	1	1
Liu 2023	34	ScoringSystem	0.780	0.050	1338	1	1
Mao 2017	35	GLM	0.920	0.010	111957	3	1
Mao 2017	35	ScoringSystem	0.760	0.010	111957	3	1
McCoy 2017	36	GLM	0.910	0.010	1665	3	1
McCoy 2017	36	ScoringSystem	0.770	0.010	1665	3	1
Moor 2023	37	NNM	0.751	0.010	136478	1	1
Moor 2023	37	DT	0.690	0.010	136478	1	1
Moor 2023	37	LR	0.656	0.010	136478	1	1
Moor 2023	37	ScoringSystem	0.761	0.010	136478	1	1
Nemati 2018 (4h)	38	LR	0.850	0.020	27527	1	1
Nemati 2018 (4h)	38	ScoringSystem	0.870	0.020	27527	1	1
Nemati 2018 (6h)	39	LR	0.850	0.020	27527	1	1
Nemati 2018 (6h)	39	ScoringSystem	0.850	0.020	27527	1	1
Nemati 2018 (8h)	40	LR	0.840	0.020	27527	1	1
Nemati 2018 (8h)	40	ScoringSystem	0.850	0.020	27527	1	1
Nemati 2018 (12h)	41	LR	0.830	0.020	27527	1	1
Nemati 2018 (12h)	41	ScoringSystem	0.820	0.020	27527	1	1
Persson 2021 (0h)	42	NNM	0.850	0.020	2893	1	1
Persson 2021 (0h)	42	ScoringSystem	0.675	0.020	2893	1	1
Persson 2021 (1h)	43	NNM	0.820	0.020	2893	1	1
Persson 2021 (1h)	43	ScoringSystem	0.595	0.020	2893	1	1
Persson 2021 (2h)	44	NNM	0.820	0.020	2893	1	1
Persson 2021 (2h)	44	ScoringSystem	0.617	0.020	2893	1	1
Persson 2021 (3h)	45	NNM	0.840	0.020	2893	1	1
Persson 2021 (3h)	45	ScoringSystem	0.644	0.020	2893	1	1
Rangan 2022 (3h)	46	DT	0.910	0.010	10500	1	1
Rangan 2022 (3h)	46	ScoringSystem	0.790	0.020	10500	1	1
Rangan 2022 (4h)	47	DT	0.860	0.020	10500	1	1
Rangan 2022 (4h)	47	ScoringSystem	0.800	0.020	10500	1	1

Rangan 2022 (5h)	48	DT	0.910	0.020	10500	1	1
Rangan 2022 (5h)	48	ScoringSystem	0.790	0.020	10500	1	1
Rangan 2022 (6h)	49	DT	0.940	0.010	10500	1	1
Rangan 2022 (6h)	49	ScoringSystem	0.790	0.020	10500	1	1
Rosnati 2021 (0h)	50	LR	0.508	0.020	22007	3	1
Rosnati 2021 (0h)	50	GLM	0.559	0.020	22007	3	1
Rosnati 2021 (0h)	50	NNM	0.647	0.020	22007	3	1
Rosnati 2021 (1h)	51	LR	0.543	0.020	22007	3	1
Rosnati 2021 (1h)	51	GLM	0.573	0.020	22007	3	1
Rosnati 2021 (1h)	51	NNM	0.664	0.020	22007	3	1
Rosnati 2021 (2h)	52	LR	0.538	0.020	22007	3	1
Rosnati 2021 (2h)	52	GLM	0.552	0.020	22007	3	1
Rosnati 2021 (2h)	52	NNM	0.670	0.020	22007	3	1
Rosnati 2021 (3h)	53	LR	0.532	0.020	22007	3	1
Rosnati 2021 (3h)	53	GLM	0.531	0.020	22007	3	1
Rosnati 2021 (3h)	53	NNM	0.665	0.020	22007	3	1
Rosnati 2021 (4h)	54	LR	0.538	0.020	22007	3	1
Rosnati 2021 (4h)	54	GLM	0.559	0.020	22007	3	1
Rosnati 2021 (4h)	54	NNM	0.675	0.020	22007	3	1
Rosnati 2021 (5h)	55	LR	0.566	0.020	22007	3	1
Rosnati 2021 (5h)	55	GLM	0.490	0.020	22007	3	1
Rosnati 2021 (5h)	55	NNM	0.664	0.020	22007	3	1
Rosnati 2021 (6h)	56	LR	0.572	0.020	22007	3	1
Rosnati 2021 (6h)	56	GLM	0.547	0.020	22007	3	1
Rosnati 2021 (6h)	56	NNM	0.641	0.020	22007	3	1
Sadasivuni 2022 (1h)	57	DT	0.920	0.020	965	3	1
Sadasivuni 2022 (1h)	57	SVM	0.920	0.020	965	3	1
Sadasivuni 2022 (1h)	57	NNM	0.920	0.020	965	3	1
Sadasivuni 2022 (1h)	57	LR	0.920	0.020	965	3	1
Sadasivuni 2022 (2h)	58	DT	0.930	0.020	965	3	1
Sadasivuni 2022 (2h)	58	SVM	0.950	0.020	965	3	1
Sadasivuni 2022 (2h)	58	NNM	0.960	0.020	965	3	1
Sadasivuni 2022 (2h)	58	LR	0.940	0.020	965	3	1
Sadasivuni 2022 (3h)	59	DT	0.915	0.020	965	3	1
Sadasivuni 2022 (3h)	59	SVM	0.930	0.020	965	3	1
Sadasivuni 2022 (3h)	59	NNM	0.920	0.020	965	3	1
Sadasivuni 2022 (3h)	59	LR	0.920	0.020	965	3	1
Sadasivuni 2022 (4h)	60	DT	0.910	0.020	965	3	1
Sadasivuni 2022 (4h)	60	SVM	0.920	0.020	965	3	1
Sadasivuni 2022 (4h)	60	NNM	0.910	0.020	965	3	1
Sadasivuni 2022 (4h)	60	LR	0.920	0.020	965	3	1
Sadasivuni 2022 (5h)	61	DT	0.920	0.020	965	3	1
Sadasivuni 2022 (5h)	61	SVM	0.920	0.020	965	3	1
Sadasivuni 2022 (5h)	61	NNM	0.900	0.020	965	3	1
Sadasivuni 2022 (5h)	61	LR	0.920	0.020	965	3	1
Sadasivuni 2022 (6h)	62	DT	0.840	0.020	965	3	1
Sadasivuni 2022 (6h)	62	SVM	0.880	0.020	965	3	1
Sadasivuni 2022 (6h)	62	NNM	0.820	0.020	965	3	1
Sadasivuni 2022 (6h)	62	LR	0.880	0.020	965	3	1
Shashikumar 3 2021 (2h)	63	NNM	0.910	0.020	25820	2	1
Shashikumar 3 2021 (2h)	63	LR	0.870	0.020	25820	2	1
Shashikumar 3 2021 (4h)	64	NNM	0.900	0.020	25820	2	1
Shashikumar 3 2021 (4h)	64	LR	0.870	0.020	25820	2	1
Shashikumar 3 2021 (6h)	65	NNM	0.890	0.020	25820	2	1
Shashikumar 3 2021 (6h)	65	LR	0.860	0.020	25820	2	1
Shashikumar 3 2021 (8h)	66	NNM	0.890	0.020	25820	2	1
Shashikumar 3 2021 (8h)	66	LR	0.860	0.020	25820	2	1
Shashikumar 3 2021 (12h)	67	NNM	0.880	0.020	25820	2	1
Shashikumar 3 2021 (12h)	67	LR	0.850	0.020	25820	2	1
Shimabukuro 2017	68	GLM	0.952	0.010	142	3	1
Shimabukuro 2017	68	ScoringSystem	0.756	0.010	142	3	1
Singh 2022	69	DT	0.950	0.020	1572	3	1
Singh 2022	69	LR	0.760	0.020	1572	3	1
Singh 2022	69	SVM	0.930	0.020	1572	3	1
Singh 2022	69	NB	0.740	0.020	1572	3	1
Taneja 2017	70	LR	0.790	0.020	444	2	1
Taneja 2017	70	SVM	0.810	0.020	444	2	1
Taneja 2017	70	DT	0.810	0.020	444	2	1
Taneja 2017	70	NB	0.800	0.020	444	2	1

Taneja 2017	70	ScoringSystem	0.540	0.020	444	2	1
Tang 2021	71	DT	0.948	0.020	2453	3	1
Tang 2021	71	LR	0.838	0.020	2453	3	1
Valik 2023	72	DT	0.963	0.010	55655	1	1
Valik 2023	72	ScoringSystem	0.905	0.010	55655	1	1
Yang 2 2022	73	LR	0.720	0.020	1383	1	1
Yang 2 2022	73	DT	0.750	0.020	1383	1	1
Yang 2 2022	73	NNM	0.730	0.020	1383	1	1
Yu 2022	74	DT	0.862	0.010	70034	1	1
Yu 2022	74	LR	0.857	0.010	70034	1	1
Yu 2022	74	ScoringSystem	0.705	0.010	70034	1	1
Yuan 2020	75	DT	0.890	0.020	1588	1	1
Yuan 2020	75	ScoringSystem	0.596	0.020	1588	1	1
Zargoush 2021	76	NNM	0.910	0.020	40336	1	1
Zargoush 2021	76	DT	0.780	0.020	40336	1	1
Zargoush 2021	76	LR	0.760	0.020	40336	1	1
Zargoush 2021	76	NB	0.680	0.020	40336	1	1
Zhang 2021	77	NNM	0.860	0.020	178843	2	1
Zhang 2021	77	LR	0.840	0.020	178843	2	1
Zhang 2021	77	DT	0.860	0.020	178843	2	1
Zhang 2021	77	ScoringSystem	0.630	0.020	178843	2	1
Zhang 2 2023	78	DT	0.760	0.010	1118	2	1
Zhang 2 2023	78	SVM	0.650	0.010	1118	2	1
Zhang 3 2023 (3h)	79	NB	0.950	0.020	4853	3	1
Zhang 3 2023 (3h)	79	LR	0.940	0.020	4853	3	1
Zhang 3 2023 (3h)	79	DT	0.940	0.020	4853	3	1
Zhang 3 2023 (3h)	79	NNM	0.940	0.020	4853	3	1
Zhang 3 2023 (3h)	79	SVM	0.940	0.020	4853	3	1
Zhang 3 2023 (3h)	79	KNN	0.910	0.020	4853	3	1
Zhang 3 2023 (6h)	80	NB	0.890	0.020	4853	3	1
Zhang 3 2023 (6h)	80	LR	0.880	0.020	4853	3	1
Zhang 3 2023 (6h)	80	DT	0.890	0.020	4853	3	1
Zhang 3 2023 (6h)	80	NNM	0.880	0.020	4853	3	1
Zhang 3 2023 (6h)	80	SVM	0.880	0.020	4853	3	1
Zhang 3 2023 (6h)	80	KNN	0.880	0.020	4853	3	1

Abbreviations: DT, Decision Tree; NNM, Neural Network Model; SVM, Support Vector Machine; LR, Logistic Regression; NB, Naïve Bayes; GLM, Generalized Linear Model; KNN, K-Nearest Neighbors; AUC, Area Under the Curve; SD, Standard Deviation; ML, Machine Learning; RoB, Risk of Bias (1-low, 2-moderate, 3-high).

Table S9. Traditional meta-analysis for ML models and traditional scoring systems for sepsis prediction.

Model	Studies, N	Pooled AUC	95% CI	p-value for overall effect	p-value for heterogeneity	I^2, %
**ML**	73	0.825	0.809-0.840	<0.001	<0.001	99.9
**SOFA**	17	0.667	0.586-0.748	<0.001	<0.001	99.9
**qSOFA**	16	0.612	0.574-0.650	<0.001	<0.001	99.8
**NEWS/NEWS2**	9	0.719	0.674-0.764	<0.001	<0.001	99.8
**MEWS**	12	0.651	0.612-0.690	<0.001	<0.001	99.7
**SIRS**	19	0.666	0.643-0.688	<0.001	<0.001	99.3
**SAPS II**	2	0.662	0.589-0.736	<0.001	<0.001	96.4

Abbreviations: ML, Machine Learning; SOFA, Sequential Organ Failure Assessment; qSOFA, quick SOFA; NEWS/NEWS2, National Early Warning Score/New National Early Warning Score; MEWS, Modified Early Warning Score; SIRS, Systemic Inflammatory Response Syndrome; SAPS II, Simplified Acute Physiology Score II; AUC, Area Under ROC Curve; CI, Confidence Interval.

13 Table S10. – Network, ML/traditional scoring systems and direct comparisons characteristics.

Network characteristics	
Number of Prediction methods	8
Number of Studies	80
Total Number of Patients in Network	36,510,863
Total Possible Pairwise Comparisons	28
Total Number of Pairwise Comparisons With Direct Data	24
Is the network connected?	TRUE
Number of Two-arm Studies	41
Number of Multi-Arms Studies	39
Average Outcome	0.873

ML/traditional scoring systems					
**Prediction method**	**Total no. of studies**	**Total no. of patients**	**Min outcome value**	**Max outcome value**	**Average outcome value**
DT	54	16060962	0.500	0.980	0.950
GLM	11	290396	0.490	0.950	0.720
KNN	8	708439	0.770	0.920	0.770
LR	45	2093892	0.510	0.940	0.780
NB	10	1014271	0.240	0.950	0.720
NNM	44	900063	0.640	0.960	0.830
ScoringSystem	40	15102630	0.520	0.910	0.830
SVM	22	340210	0.390	0.950	0.790

Direct comparisons characteristics		
**Comparison**	**Total no. of studies**	**Total no. of patients**
DT vs. KNN	8	1416878
DT vs. LR	27	3384584
DT vs. NB	10	2028542
DT vs. NNM	27	1209484
DT vs. ScoringSystem	27	29673720
DT vs. SVM	21	679220
GLM vs. LR	7	308098
GLM vs. NNM	7	308098
GLM vs. ScoringSystem	4	272694
KNN vs. LR	5	1402616
KNN vs. NB	4	1399940
KNN vs. NNM	7	46658
KNN vs. ScoringSystem	2	7430
KNN vs. SVM	7	46658
LR vs. NB	9	1947870
LR vs. NNM	30	1680280
LR vs. ScoringSystem	13	1364702
LR vs. SVM	15	512434
NB vs. NNM	5	111746
NB vs. ScoringSystem	2	2242
NB vs. SVM	8	577650
NNM vs. ScoringSystem	15	1025634
NNM vs. SVM	16	104152
ScoringSystem vs. SVM	4	9672

Abbreviations: DT, Decision Tree; NNM, Neural Network Model; SVM, Support Vector Machine; LR, Logistic Regression; NB, Naïve Bayes; GLM, Generalized Linear Model; KNN, K-Nearest Neighbors.

14 Table S11. League table.

**DT**	0.06 (0.00, 0.12)	0.030 (-0.03, 0.09)	**0.05 (0.02, 0.09)**	**0.09 (0.04, 0.15)**	-0.10 (-0.04, 0.03)	**0.16 (0.13, 0.19)**	**0.08 (0.04, 0.12)**
-0.06 (-0.12,0.00)	**GLM**	-0.030 (-0.11, 0.05)	-0.01 (-0.07, 0.05)	0.03 (-0.05, 0.11)	**-0.07 (-0.13, -0.01)**	**0.10 (0.04, 0.16)**	0.02 (-0.04, 0.09)
-0.03 (-0.09,0.03)	0.03 (-0.05, 0.11)	**KNN**	0.02 (-0.04, 0.08)	0.06 (-0.01, 0.13)	-0.04 (-0.10, 0.02)	**0.13 (0.07, 0.19)**	0.05 (-0.01, 0.12)
**-0.05 (-0.09, -0.02)**	0.01 (-0.05, 0.07)	-0.02 (-0.08, 0.04)	**LR**	0.04 (-0.02, 0.09)	**-0.06 (-0.09, -0.03)**	**0.11 (0.07, 0.14)**	0.03 (-0.01, 0.07)
**-0.09 (-0.12, -0.04)**	-0.03 (-0.11, 0.05)	-0.060 (-0.13, 0.01)	-0.04 (-0.09, 0.02)	**NB**	**-0.10 (-0.15, -0.04)**	**0.07 (0.01, 0.13)**	-0.01 (-0.07, 0.05)
0.01 (-0.03, 0.04)	**0.07 (0.01, 0.13)**	0.04 (-0.02, 0.10)	**0.06 (0.03, 0.09)**	**0.10 (0.04, 0.15)**	**NNM**	**0.17 (0.13, 0.20)**	**0.091 (0.05, 0.13)**
**-0.16 (-0.19, -0.13)**	**-0.10 (-0.16, -0.04)**	**-0.13 (-0.19, -0.07)**	**-0.11 (-0.14, -0.07)**	**-0.07 (-0.13, -0.01)**	**-0.17 (-0.20, -0.13)**	**Scoring System**	**-0.08 (-0.12, -0.03)**
**-0.083 (-0.12, -0.04)**	-0.02 (-0.09, 0.04)	-0.05 (-0.12, 0.01)	-0.03 (-0.07, 0.01)	0.01 (-0.05, 0.07)	**-0.09 (-0.13, -0.05)**	**0.08 (0.03, 0.12)**	**SVM**

Abbreviations: DT, Decision Tree; NNM, Neural Network Model; SVM, Support Vector Machine; LR, Logistic Regression; NB, Naïve Bayes; GLM, Generalized Linear Model; KNN, K-Nearest Neighbors.

15 Table S12. League table for studies with low-moderate bias.

**DT**	0.07 (-0.15, 0.29)	0.04 (-0.04, 0.12)	**0.07 (0.03, 0.12)**	**0.12 (0.05, 0.20)**	0.01 (-0.04, 0.06)	**0.15 (0.11, 0.20)**	**0.13 (0.07, 0.19)**
-0.07 (-0.29, 0.15)	**GLM**	-0.04 (-0.26, 0.20)	0.01 (-0.22, 0.22)	0.05 (-0.18, 0.28)	-0.06 (-0.28, 0.16)	0.08 (-0.13, 0.29)	0.06 (-0.16, 0.28)
-0.04 (-0.12, 0.04)	0.04 (-0.20, 0.26)	**KNN**	0.04 (-0.05, 0.12)	0.08 (-0.02, 0.18)	-0.03 (-0.11, 0.05)	**0.12 (0.03, 0.20)**	**0.09 (0.01, 0.18)**
**-0.07 (-0.12, -0.03)**	-0.01 (-0.22, 0.2)	-0.04 (-0.12, 0.05)	**LR**	0.05 (-0.03, 0.12)	**-0.07 (-0.12, -0.02)**	**0.08 (0.03, 0.13)**	0.06 (-0.01, 0.12)
**-0.12 (-0.20, -0.05)**	-0.05 (-0.28, 0.18)	-0.08 (-0.18, 0.02)	-0.05 (-0.12, 0.03)	**NB**	**-0.11 (-0.20, -0.03)**	0.03 (-0.05, 0.11)	0.01 (-0.07, 0.09)
-0.01 (-0.06, 0.04)	0.06 (-0.12, 0.28)	0.03 (-0.05, 0.11)	**0.07 (0.02, 0.12)**	**0.11 (0.03, 0.19)**	**NNM**	**0.14 (0.09, 0.19)**	**0.12 (0.06, 0.19)**
**-0.15 (-0.20, -0.11)**	-0.08 (-0.29, 0.13)	**-0.12 (-0.20, -0.03)**	**-0.08 (-0.13, -0.03)**	-0.03 (-0.11, 0.05)	**-0.14 (-0.20, -0.09)**	**Scoring System**	-0.02 (-0.09, 0.04)
**-0.13 (-0.19, -0.07)**	-0.06 (-0.28, 0.16)	**-0.09 (-0.18, -0.01)**	-0.06 (-0.12, 0.01)	-0.01 (-0.09, 0.07)	**-0.12 (-0.19, -0.06)**	0.02 (-0.04, 0.09)	**SVM**

Abbreviations: DT, Decision Tree; NNM, Neural Network Model; SVM, Support Vector Machine; LR, Logistic Regression; NB, Naïve Bayes; GLM, Generalized Linear Model; KNN, K-Nearest Neighbors.

16 **Table S13. Meta-regression.**

Variables	N, models	Univariate analysis			Multivariable analysis		
		Coeff	SE	p-value	Coeff	SE	p-value
Total sample size	256	$3.99*10^{-8}$	$2.28*10^{-8}$	0.081			
Sepsis prevalence	248	0.0002	0.0004	0.673			
Mean age	160	-0.0027	0.0015	0.079			
**Percentage of men**	**191**	**-0.0029**	**0.0010**	**0.004**	-0.0015	0.0014	0.910
Mortality	80	0.0005	0.0020	0.800			
**ML model type**	**256**	**-0.0244**	**0.0045**	**<0.001**	**-0.0123**	**0.0045**	**0.006**
**Dataset (Ref. open dataset vs. other)**	**256**	**0.0610**	**0.0156**	**<0.001**	**0.0334**	**0.0096**	**<0.001**
**Outcome definition (Ref. Sepsis-3)**	**256**	**0.0610**	**0.0156**	**<0.001**	0.0115	0.0145	0.427
Department (ED vs. ICU vs. in-hospital)	256	-0.0134	0.0136	0.323			
**Prediction window**[†]	**256**	**-0.0006**	**0.0003**	**0.049**	**-0.0010**	**0.0003**	**0.004**
External validation (Ref. none)	256	0.0265	0.0183	0.147			
**Imputation as a feature (Ref. none)**[#]	**256**	**-0.0433**	**0.0162**	**0.008**	0.0153	0.0140	0.275
Body temperature as a feature (Ref. none)	230	-0.0162	0.0376	0.666			
Lab. values as a feature (Ref. none)	254	-0.0400	0.0240	0.096			
Heart rate as a feature (Ref. none)	230	-0.0251	0.0344	0.465			
Respiratory rate as a feature (Ref. none)	230	-0.0221	0.0355	0.534			
Number of features	231	-0.0001	0.0001	0.441			

Abbreviations: Ref., reference; SE, Standard Error; ML, Machine Learning; ED, Emergence Department; ICU, Intensive Care Unit.,

Meta-regression allows to identify which characteristics of the study could affect the result of the meta-analysis (effect size). In the context of our study, meta-regression allows to identify which characteristics of the studies (study-level variations) could affect predictive efficacy of ML models in sepsis prediction. Covariance coefficients, standard error and p-values are presented.

For a correct meta-regression, The Cochrane handbook suggests a minimum of 10 studies for each study-level variable without providing justifications, and there should be at least 6 to 10 studies for a continuous study level variable. For a categorical subgroup variable, each subgroup should have a minimum of 4 studies.

[†]Prediction window. The period from which the dependent variable, also called the target, outcome, or event, is sampled. It is the period for which the model predicts the development of sepsis in the future.

[#]Imputation is a method used to fill in missing data in datasets. It's important because it helps maintain the completeness of the data, reduces bias caused by missing information, and increases the reliability and accuracy of statistical analyses. Proper imputation ensures that analyses are more representative of the whole dataset.

Table S14. Network meta-analysis test for small-study effects.

№	Comparison	bias	p-value	Interpretation
1	DT vs LR	47.34	0.810	Small studies favour 1st intervention
2	DT vs NB	297.97	0.510	Small studies favour 1st intervention
3	DT vs NNM	52.24	0.470	Small studies favour 1st intervention
4	DT vs ScoringSystem	616.63	0.120	Small studies favour 1st intervention
5	DT vs SVM	209.08	0.030	Small studies favour 1st intervention
6	LR vs NNM	-74.50	0.490	Small studies favour 2nd intervention
7	LR vs ScoringSystem	807.59	0.330	Small studies favour 1st intervention
8	LR vs SVM	40.21	0.590	Small studies favour 1st intervention
9	NNM vs ScoringSystem	439.01	0.270	Small studies favour 1st intervention
10	NNM vs SVM	-285.61	0.170	Small studies favour 2nd intervention

Table S15. Per-comparison contribution matrix.

Random MD	DT:KNN	DT:LR	DT:NB	DT:NNM	DT:ScoringSystem	DT:SVM	GLM:LR	GLM:NNM	GLM:ScoringSystem	KNN:LR	KNN:NB	KNN:NNM	KNN:ScoringSystem	KNN:SVM	LR:NB	LR:NNM	LR:ScoringSystem	LR:SVM	NB:NNM	NB:ScoringSystem	NB:SVM	NNM:ScoringSystem	NNM:SVM	ScoringSystem:SVM
**Mixed estimates**																								
DT:KNN	28.4	8.1	4	9	5	7.9	0	0.1	0.1	7.3	4.9	9.7	3.1	8.9	0.5	0.3	0.5	0.5	0.2	0.2	0.1	0.8	0.3	0.4
DT:LR	2.4	39.5	3.4	9.8	7.1	6.4	0.9	0.4	0.5	2.1	0.1	0.2	0	0	3.5	10.6	5.7	6.1	0	0.1	0	0.7	0.3	0
DT:NB	3.2	9.1	31.3	7.5	4.7	7.7	0.1	0	0.1	0.2	4.1	0.5	0.2	0.1	10.4	0.7	0.8	0.2	6.3	2.7	8.7	0.5	0.5	0.4
DT:NNM	2.7	9.8	2.7	39.3	7.5	6.6	0.4	0.9	0.5	0.1	0.1	2.7	0	0	0.4	10.6	0.5	0.3	2.1	0	0.1	6.4	6.5	0
DT:SVM	3	8.7	3.7	8.7	4.9	39.3	0	0.1	0.1	0.1	0	0.2	0.2	3.5	0.2	0	0.7	9	0.1	0.2	4.1	0.9	9.4	2.8
DT:ScoringSystem	1.7	7.9	1.9	8.1	49.9	3.9	0.5	0.4	0.9	0.2	0	0.2	1.4	0	0.3	0.1	8.1	0.4	0.2	1.4	0	8.9	0.7	2.8
GLM:LR	0	4.3	0.2	2.3	2.4	0.2	42.1	16	8.4	0.8	0.1	0.6	0.2	0	1.1	11.1	5	2.1	0.6	0.2	0.1	0.1	1.5	0.4
GLM:NNM	0.1	2.3	0	4.3	2.3	0.2	16	42	8.7	0.5	0.1	0.9	0.2	0	0.8	10.9	0	1.5	0.8	0.1	0.1	5.6	2.2	0.4
GLM:ScoringSystem	0.2	3.6	0.3	3.4	8.3	0.7	15.1	15.3	26.5	0.5	0	0.5	0.7	0	0.8	0.1	8.9	1.3	0.3	0.7	0	10.2	1	1.5
KNN:LR	11.5	11.3	0.3	0.4	0.7	0.5	0.7	0.5	0.2	18.8	5	10.4	3	8.5	4.3	11.2	4	7.1	0.3	0.1	0	0.1	0.7	0.3
KNN:NB	11	0.3	11.5	0.5	0.1	0.1	0.1	0.1	0	7	20.4	8.1	2.6	9.5	9	1.1	0.3	0.3	6	2.4	9.4	0.1	0.2	0
KNN:NNM	11.3	0.7	0.5	10.7	0.8	0.4	0.5	0.6	0.2	7.5	4.1	25.7	3	8.6	0.9	9.4	0	0.9	2.5	0.2	0.1	4.1	7.1	0.3
KNN:SVM	11.2	0.1	0.2	0.2	0.1	11.6	0	0	0	7	5.2	9.5	2.6	27.5	0.2	0.3	0.1	7.8	0.1	0	4.7	0	9.1	2.3
KNN:ScoringSystem	12.3	0.3	1.1	0.4	16.1	2	0.5	0.6	1.1	7.6	4.1	10.5	8.9	7.1	0.9	0.1	8.6	1	0.4	1.7	0.1	10.7	0.9	3.3
LR:NB	0.3	10.6	11.7	1.1	0.7	0.4	0.6	0.4	0.2	2.4	3.7	0.7	0.2	0.1	29.2	8.8	3.6	6.6	6.5	2.6	8.2	0	1	0.3
LR:NNM	0.1	9.9	0.2	9.9	0.1	0	2.6	2.6	0	1.9	0.1	2.2	0	0.1	2.6	44.1	4.9	5.6	2	0.1	0.2	5.1	5.7	0
LR:SVM	0.2	11.1	0.1	0.6	1	12.3	0.7	0.5	0.2	2.4	0	0.3	0.2	3.1	4	10.9	3.9	30.7	0.2	0.1	4.3	0.1	10.5	2.7
LR:ScoringSystem	0.4	11.9	0.7	1	15.2	1.3	2.7	0	2.7	1.7	0.1	0	1.4	0	2.4	11.1	27.9	4.4	0.1	1.5	0	10.4	0.2	2.9
NB:NNM	0.3	0.2	12.3	11.5	1.1	0.4	0.6	0.7	0.2	0.3	4.2	3.4	0.2	0	11.6	11.9	0.2	0.5	16.9	2.7	8.7	4.3	7.5	0.3
NB:SVM	0.1	0.2	12	0.3	0	12	0.1	0.1	0	0	4.5	0.2	0	4.1	9.9	0.8	0.1	8.8	6.1	2.4	28.3	0.2	7.7	2.3
NB:ScoringSystem	0.8	0.8	13.4	0	16.9	1.9	0.6	0.4	1	0.3	3.6	0.7	1.7	0	10.9	0.7	9.6	0.6	6.7	8.1	7.2	9.2	1.4	3.3
NNM:SVM	0.2	0.7	0.3	10.9	1.1	12.2	0.5	0.7	0.2	0.2	0	3.1	0.2	3.6	0.5	10.5	0.1	9.8	2.4	0.2	3.3	4.1	32.5	2.7
NNM:ScoringSystem	0.5	1.2	0.4	11.5	15	1.3	0.1	2.6	2.7	0.1	0	2	1.4	0	0	10.1	9	0.2	1.7	1.4	0.1	31.5	4.4	2.9
ScoringSystem:SVM	0.7	0.5	0.9	0.3	17.3	14.8	0.6	0.5	1.1	0.3	0	0.4	1.7	3	0.5	0.1	9.4	9.9	0.3	1.7	3.3	10.7	10.9	11.2
**Indirect estimates**																								
DT:GLM	1.6	13.2	1.9	13.2	11.6	4.1	17.3	17.2	11.3	0.6	0	0.9	0.1	0	1.1	0	0.4	1.9	0.6	0.1	0	0.4	2	0.3
GLM:KNN	7.8	3.2	0.5	2.8	3.1	0.8	15.6	16.8	7.9	8.2	3.4	11.4	3.3	6.1	1.6	0.2	0.1	2.5	0.9	0.4	0.1	0.3	2.2	0.7
GLM:NB	0.4	2.8	8.4	3.4	3.3	0.7	17.3	15.2	7.8	0.8	3	1.3	0.4	0.1	12.8	0.5	0.3	1.8	7.3	2.9	5.9	0.1	2.7	0.7
GLM:SVM	0.3	3	0.3	3	3.5	8.9	17	17.3	8.1	0.8	0	1.1	0.4	2.5	1.4	0	0.2	12	0.7	0.4	2.7	0.3	12.8	3.4

Abbreviations: DT, Decision Tree; NNM, Neural Network Model; SVM, Support Vector Machine; LR, Logistic Regression; NB, Naïve Bayes; GLM, Generalized Linear Model; KNN, K-Nearest Neighbors.

Columns refer to comparisons with direct data and rows refer to NMA relative comparisons.

Table S16. Percentage contribution matrix.

Study	Mixed estimates																								Indirect estimates			
	DT: KNN	DT: LR	DT: NB	DT: NNM	DT: SVM	DT: ScoringSystem	GLM: LR	GLM: NNM	GLM: ScoringSystem	KNN: LR	KNN: NB	KNN: NNM	KNN: SVM	KNN: ScoringSystem	LR: NB	LR: NNM	LR: SVM	LR: ScoringSystem	NB: NNM	NB: SVM	NB: ScoringSystem	NNM: SVM	NNM: ScoringSystem	ScoringSystem: SVM	DT: GLM	GLM: KNN	GLM: NB	GLM: SVM
Abromavicius, 2020	0.8	0.6	4.6	0.6	2.8	0.4	0	0	0.1	0.1	2.3	0.1	1.2	0.2	2.2	0	1.1	0.1	2.3	5.3	2.3	1	0.1	1.2	0.4	0.1	1.6	0.8
Aşurŏglu, 2020	10.5	4.4	9.2	4.4	5.6	1.4	1.4	1.4	0.8	11.1	15	10.7	11.7	7.1	9.5	4.4	6.1	2	10	10.5	6.2	6	1.9	3.3	2.3	7	6.2	3.6
Bao, 2022	0.7	0.7	0.7	2.2	2.8	0.5	0.2	0.3	0.2	0.1	0	0.9	1.1	0.2	0.1	0.7	1.3	0.1	0.9	1.1	0.2	3	0.8	1.4	0.8	0.3	0.3	1.3
Barton, 2019	0.2	0.3	0.2	0.3	0.2	1.8	0.1	0.1	0.3	0	0	0	0	0.6	0	0	0	0.6	0	0	0.6	0	0.6	0.6	0.4	0.1	0.1	0.1
Bedoya, 2020	0.9	2.9	0.9	2.9	0.9	3.7	1.1	1.1	1.9	1.1	0.1	1	0	2	1	2.9	1.1	4.3	1.2	0.1	2	1.1	4.2	2.1	1.5	0.4	0.4	0.4
Bloch, 2019	0.1	0.8	0.1	0.8	1.2	0.1	0.6	0.6	0.1	0.9	0.1	0.8	1.1	0.1	0.8	2.2	3.1	0.7	0.9	1.1	0.2	3	0.6	1.3	0.3	0.3	0.3	1.6
Burdick, 2020	0.2	0.3	0.2	0.3	0.2	1.8	0.1	0.1	0.3	0	0	0	0	0.6	0	0	0	0.6	0	0	0.6	0	0.6	0.6	0.4	0.1	0.1	0.1
Camacho-Cogollo, 2022 (1h)	8.8	1.3	1.6	3.6	4.7	4.6	0.6	1.1	2.1	5.8	5.2	9.1	9.7	10.8	0.5	1.4	2.6	2.9	1.9	2.3	3.3	5.1	5.2	7	1.7	5.7	1.1	3.1
Camacho-Cogollo, 2022 (2h)	6.9	1	1.2	2.9	3.7	0.8	0.3	0.5	0.3	4.2	3.9	7.2	7.8	4.2	0.3	1.1	1.8	0.2	1.4	1.7	0.4	4	1.1	2	1.1	3.8	0.6	1.9
Camacho-Cogollo, 2022 (3h)	6.9	1	1.2	2.9	3.7	0.8	0.3	0.5	0.3	4.2	3.9	7.2	7.8	4.2	0.3	1.1	1.8	0.2	1.4	1.7	0.4	4	1.1	2	1.1	3.8	0.6	1.9
Chen, 2022	0.3	0.4	0.3	1.5	0.3	0.3	0.1	0.2	0.1	0	0	0.4	0	0	0	0.4	0	0	0.4	0	0	0.4	0.4	0	0.5	0.1	0.1	0.1
Chen, 2023	2	4.4	9.4	4.4	5.7	5.6	1.8	1.7	3	2.4	5.8	2.4	2.9	4.2	9.6	4.4	6.2	6.4	10.1	10.5	11.9	6.1	6.3	8.4	2.5	1.5	6.8	4.1
Choi, 2020	0	0.4	0.1	0	0.1	0.6	0.4	0	0.7	0.3	0	0	0	0.7	0.3	0.4	0.3	2.1	0	0	0.7	0	0.7	0.7	0	0	0	0
Delahanty, 2019 (1h)	0.2	0.3	0.2	0.3	0.2	1.8	0.1	0.1	0.3	0	0	0	0	0.6	0	0	0	0.6	0	0	0.6	0	0.6	0.6	0.4	0.1	0.1	0.1
Delahanty, 2019 (3h)	0.2	0.3	0.2	0.3	0.2	1.8	0.1	0.1	0.3	0	0	0	0	0.6	0	0	0	0.6	0	0	0.6	0	0.6	0.6	0.4	0.1	0.1	0.1
Delahanty, 2019 (6h)	0.2	0.3	0.2	0.3	0.2	1.8	0.1	0.1	0.3	0	0	0	0	0.6	0	0	0	0.6	0	0	0.6	0	0.6	0.6	0.4	0.1	0.1	0.1
Delahanty, 2019 (12h)	0.2	0.3	0.2	0.3	0.2	1.8	0.1	0.1	0.3	0	0	0	0	0.6	0	0	0	0.6	0	0	0.6	0	0.6	0.6	0.4	0.1	0.1	0.1
Delahanty, 2019 (24h)	0.2	0.3	0.2	0.3	0.2	1.8	0.1	0.1	0.3	0	0	0	0	0.6	0	0	0	0.6	0	0	0.6	0	0.6	0.6	0.4	0.1	0.1	0.1
Desautels, 2016	0	0.1	0	0.1	0	0.2	2.1	2.2	6.6	0	0	0	0	0.3	0	0	0	0.7	0	0	0.3	0	0.7	0.3	2.8	2	1.9	2
Duan. 2023 (6h)	0.6	0.7	0.5	2.2	0.6	2.7	0.2	0.6	1.1	0	0	0.7	0	1.3	0.1	0.7	0.1	1.3	0.8	0	1.2	0.7	3.1	1.4	0.9	0.2	0.3	0.3
Duan. 2023 (12h)	0.6	0.7	0.5	2.2	0.6	2.7	0.2	0.6	1.1	0	0	0.7	0	1.3	0.1	0.7	0.1	1.3	0.8	0	1.2	0.7	3.1	1.4	0.9	0.2	0.3	0.3
Duan. 2023 (24h)	0.6	0.7	0.5	2.2	0.6	2.7	0.2	0.6	1.1	0	0	0.7	0	1.3	0.1	0.7	0.1	1.3	0.8	0	1.2	0.7	3.1	1.4	0.9	0.2	0.3	0.3
El-Rashidy, 2022	0.6	0.7	0.5	2.2	0.6	2.7	0.2	0.6	1.1	0	0	0.7	0	1.3	0.1	0.7	0.1	1.3	0.8	0	1.2	0.7	3.1	1.4	0.9	0.2	0.3	0.3
Gholamzadeh, 2023	7	2.9	6.1	1	1.1	0.8	0.5	0.3	0.4	7.4	10	4.1	4.2	4.3	6.3	1.1	1.4	1.2	3.7	3.4	3.6	0.2	0.2	0.3	1.1	3.8	3.3	0.5
Goh, 2021 (4h)	0.3	1.5	0.3	0.4	0.3	0.3	0.2	0.1	0.1	0.4	0	0	0	0	0.4	0.4	0.4	0.4	0	0	0	0	0	0	0.5	0.1	0.1	0.1
Goh, 2021 (6h)	0.3	1.5	0.3	0.4	0.3	0.3	0.2	0.1	0.1	0.4	0	0	0	0	0.4	0.4	0.4	0.4	0	0	0	0	0	0	0.5	0.1	0.1	0.1
Goh, 2021 (12h)	0.3	1.5	0.3	0.4	0.3	0.3	0.2	0.1	0.1	0.4	0	0	0	0	0.4	0.4	0.4	0.4	0	0	0	0	0	0	0.5	0.1	0.1	0.1
Horng, 2017	1.2	2.9	6.1	1	3.7	0.7	0.5	0.3	0.4	1.4	3.4	0.3	1.7	0.4	6.3	1.1	4	1.1	3.7	7	3.6	1.8	0.2	1.9	1.1	0.6	3.3	1.9
Ibrahim, 2019	0.4	0.3	0.4	0.3	1.9	0.2	0	0	0	0	0	0	0.6	0.1	0	0	0.6	0.1	0	0.6	0.1	0.6	0.1	0.7	0.2	0	0	0.4
Kijpaisalratana, 2022	0.9	2.9	0.9	2.9	0.9	3.7	1.1	1.1	1.9	1.1	0.1	1	0	2	1	2.9	1.1	4.3	1.2	0.1	2	1.1	4.2	2.1	1.5	0.4	0.4	0.4
Lauritsen 2, 2021	0.3	1.5	0.3	0.4	0.3	0.3	0.2	0.1	0.1	0.4	0	0	0	0	0.4	0.4	0.4	0.4	0	0	0	0	0	0	0.5	0.1	0.1	0.1
Lin, 2021	0.2	0.3	0.2	0.3	0.2	1.8	0.1	0.1	0.3	0	0	0	0	0.6	0	0	0	0.6	0	0	0.6	0	0.6	0.6	0.4	0.1	0.1	0.1
Liu, 2022	0.3	0.4	0.3	1.5	0.3	0.3	0.1	0.2	0.1	0	0	0.4	0	0	0	0.4	0	0	0.4	0	0	0.4	0.4	0	0.5	0.1	0.1	0.1
Liu, 2023	10.6	4.4	2	4.4	5.7	5.6	1.8	1.8	3.1	11.2	6.7	10.9	11.6	13	2.3	4.4	6.2	6.4	2.4	2.9	4.2	6.1	6.3	8.4	2.5	7.7	1.5	4.2
Mao, 2017	0	0.1	0	0.1	0	0.2	2.1	2.2	6.6	0	0	0	0	0.3	0	0	0	0.7	0	0	0.3	0	0.7	0.3	2.8	2	1.9	2
McCoy, 2017	0	0.1	0	0.1	0	0.2	2.1	2.2	6.6	0	0	0	0	0.3	0	0	0	0.7	0	0	0.3	0	0.7	0.3	2.8	2	1.9	2
Moor, 2023	0.9	2.9	0.9	2.9	0.9	3.7	1.1	1.1	1.9	1.1	0.1	1	0	2	1	2.9	1.1	4.3	1.2	0.1	2	1.1	4.2	2.1	1.5	0.4	0.4	0.4
Nemati, 2018 (4h)	0	0.4	0.1	0	0.1	0.6	0.4	0	0.7	0.3	0	0	0	0.7	0.3	0.4	0.3	2.1	0	0	0.7	0	0.7	0.7	0	0	0	0
Nemati, 2018 (6h)	0	0.4	0.1	0	0.1	0.6	0.4	0	0.7	0.3	0	0	0	0.7	0.3	0.4	0.3	2.1	0	0	0.7	0	0.7	0.7	0	0	0	0
Nemati, 2018 (8h)	0	0.4	0.1	0	0.1	0.6	0.4	0	0.7	0.3	0	0	0	0.7	0.3	0.4	0.3	2.1	0	0	0.7	0	0.7	0.7	0	0	0	0
Nemati, 2018 (12h)	0	0.4	0.1	0	0.1	0.6	0.4	0	0.7	0.3	0	0	0	0.7	0.3	0.4	0.3	2.1	0	0	0.7	0	0.7	0.7	0	0	0	0
Persson, 2021 (0h)	0.1	0	0	0.4	0.1	0.6	0	0.4	0.7	0	0	0.3	0	0.7	0	0.3	0	0.7	0.3	0	0.6	0.3	2.1	0.7	0	0	0	0
Persson, 2021 (1h)	0.1	0	0	0.4	0.1	0.6	0	0.4	0.7	0	0	0.3	0	0.7	0	0.3	0	0.7	0.3	0	0.6	0.3	2.1	0.7	0	0	0	0
Persson, 2021 (2h)	0.1	0	0	0.4	0.1	0.6	0	0.4	0.7	0	0	0.3	0	0.7	0	0.3	0	0.7	0.3	0	0.6	0.3	2.1	0.7	0	0	0	0
Persson, 2021 (3h)	0.1	0	0	0.4	0.1	0.6	0	0.4	0.7	0	0	0.3	0	0.7	0	0.3	0	0.7	0.3	0	0.6	0.3	2.1	0.7	0	0	0	0
Rangan, 2022 (3h)	0.2	0.3	0.2	0.3	0.2	1.8	0.1	0.1	0.3	0	0	0	0	0.6	0	0	0	0.6	0	0	0.6	0	0.6	0.6	0.4	0.1	0.1	0.1
Rangan, 2022 (4h)	0.2	0.3	0.2	0.3	0.2	1.8	0.1	0.1	0.3	0	0	0	0	0.6	0	0	0	0.6	0	0	0.6	0	0.6	0.6	0.4	0.1	0.1	0.1
Rangan, 2022 (5h)	0.2	0.3	0.2	0.3	0.2	1.8	0.1	0.1	0.3	0	0	0	0	0.6	0	0	0	0.6	0	0	0.6	0	0.6	0.6	0.4	0.1	0.1	0.1
Rangan, 2022 (6h)	0.2	0.3	0.2	0.3	0.2	1.8	0.1	0.1	0.3	0	0	0	0	0.6	0	0	0	0.6	0	0	0.6	0	0.6	0.6	0.4	0.1	0.1	0.1
Rosnati, 2021 (0h)	0	0.6	0	0.5	0	0.1	8.7	8.6	4.3	0.6	0.1	0.5	0	0.2	0.4	2.2	0.5	0.8	0.6	0	0.2	0.5	0.7	0.2	4.9	4.6	4.7	4.9
Rosnati, 2021 (1h)	0	0.6	0	0.5	0	0.1	8.7	8.6	4.3	0.6	0.1	0.5	0	0.2	0.4	2.2	0.5	0.8	0.6	0	0.2	0.5	0.7	0.2	4.9	4.6	4.7	4.9
Rosnati, 2021 (2h)	0	0.6	0	0.5	0	0.1	8.7	8.6	4.3	0.6	0.1	0.5	0	0.2	0.4	2.2	0.5	0.8	0.6	0	0.2	0.5	0.7	0.2	4.9	4.6	4.7	4.9
Rosnati, 2021 (3h)	0	0.6	0	0.5	0	0.1	8.7	8.6	4.3	0.6	0.1	0.5	0	0.2	0.4	2.2	0.5	0.8	0.6	0	0.2	0.5	0.7	0.2	4.9	4.6	4.7	4.9
Rosnati, 2021 (4h)	0	0.6	0	0.5	0	0.1	8.7	8.6	4.3	0.6	0.1	0.5	0	0.2	0.4	2.2	0.5	0.8	0.6	0	0.2	0.5	0.7	0.2	4.9	4.6	4.7	4.9
Rosnati, 2021 (5h)	0	0.6	0	0.5	0	0.1	8.7	8.6	4.3	0.6	0.1	0.5	0	0.2	0.4	2.2	0.5	0.8	0.6	0	0.2	0.5	0.7	0.2	4.9	4.6	4.7	4.9
Rosnati, 2021 (6h)	0	0.6	0	0.5	0	0.1	8.7	8.6	4.3	0.6	0.1	0.5	0	0.2	0.4	2.2	0.5	0.8	0.6	0	0.2	0.5	0.7	0.2	4.9	4.6	4.7	4.9
Sadasivuni, 2022 (1h)	1.1	2.9	1.1	2.9	3.7	0.8	0.9	0.9	0.4	1.3	0.1	1.3	1.7	0.2	1.3	2.9	4.1	1.2	1.4	1.7	0.3	4	1.2	2.1	1.4	0.6	0.6	2.3
Sadasivuni, 2022 (2h)	1.1	2.9	1.1	2.9	3.7	0.8	0.9	0.9	0.4	1.3	0.1	1.3	1.7	0.2	1.3	2.9	4.1	1.2	1.4	1.7	0.3	4	1.2	2.1	1.4	0.6	0.6	2.3
Sadasivuni, 2022 (3h)	1.1	2.9	1.1	2.9	3.7	0.8	0.9	0.9	0.4	1.3	0.1	1.3	1.7	0.2	1.3	2.9	4.1	1.2	1.4	1.7	0.3	4	1.2	2.1	1.4	0.6	0.6	2.3
Sadasivuni, 2022 (4h)	1.1	2.9	1.1	2.9	3.7	0.8	0.9	0.9	0.4	1.3	0.1	1.3	1.7	0.2	1.3	2.9	4.1	1.2	1.4	1.7	0.3	4	1.2	2.1	1.4	0.6	0.6	2.3
Sadasivuni, 2022 (5h)	1.1	2.9	1.1	2.9	3.7	0.8	0.9	0.9	0.4	1.3	0.1	1.3	1.7	0.2	1.3	2.9	4.1	1.2	1.4	1.7	0.3	4	1.2	2.1	1.4	0.6	0.6	2.3
Sadasivuni, 2022 (6h)	1.1	2.9	1.1	2.9	3.7	0.8	0.9	0.9	0.4	1.3	0.1	1.3	1.7	0.2	1.3	2.9	4.1	1.2	1.4	1.7	0.3	4	1.2	2.1	1.4	0.6	0.6	2.3
Shashikumar 3, 2021 (2h)	0	0.4	0	0.4	0	0	0.4	0.4	0	0.4	0	0.3	0	0	0.3	1.5	0.4	0.4	0.4	0	0	0.4	0.3	0	0	0	0	0
Shashikumar 3, 2021 (4h)	0	0.4	0	0.4	0	0	0.4	0.4	0	0.4	0	0.3	0	0	0.3	1.5	0.4	0.4	0.4	0	0	0.4	0.3	0	0	0	0	0
Shashikumar 3, 2021 (6h)	0	0.4	0	0.4	0	0	0.4	0.4	0	0.4	0	0.3	0	0	0.3	1.5	0.4	0.4	0.4	0	0	0.4	0.3	0	0	0	0	0
Shashikumar 3, 2021 (8h)	0	0.4	0	0.4	0	0	0.4	0.4	0	0.4	0	0.3	0	0	0.3	1.5	0.4	0.4	0.4	0	0	0.4	0.3	0	0	0	0	0
Shashikumar 3, 2021 (12h)	0	0.4	0	0.4	0	0	0.4	0.4	0	0.4	0	0.3	0	0	0.3	1.5	0.4	0.4	0.4	0	0	0.4	0.3	0	0	0	0	0

Shimabukuro, 2017	0	0.1	0	0.1	0	0.2	2.1	2.2	6.6	0	0	0	0	0.3	0	0	0	0.7	0	0	0.3	0	0.7	0.3	2.8	2	1.9	2
Singh, 2022	1.2	2.9	6.1	1	3.7	0.7	0.5	0.3	0.4	1.4	3.4	0.3	1.7	0.4	6.3	1.1	4	1.1	3.7	7	3.6	1.8	0.2	1.9	1.1	0.6	3.3	1.9
Taneja, 2017	1.6	3.7	7.8	1.3	4.7	4.6	1.2	0.6	2.1	1.9	4.6	0.5	2.3	3.3	8	1.5	5.1	5.3	5.1	8.7	9.9	2.6	2.8	6.9	1.7	1.1	5.1	3
Tang, 2021	0.3	1.5	0.3	0.4	0.3	0.3	0.2	0.1	0.1	0.4	0	0	0	0	0.4	0.4	0.4	0.4	0	0	0	0	0	0	0.5	0.1	0.1	0.1
Valik, 2023	0.2	0.3	0.2	0.3	0.2	1.8	0.1	0.1	0.3	0	0	0	0	0.6	0	0	0	0.6	0	0	0.6	0	0.6	0.6	0.4	0.1	0.1	0.1
Yang 2, 2022	0.6	2.2	0.6	2.2	0.6	0.6	0.6	0.6	0.3	0.8	0.1	0.7	0	0	0.7	2.2	0.8	0.8	0.8	0	0.1	0.8	0.8	0	1	0.2	0.2	0.2
Yu, 2022	0.5	2.2	0.6	0.7	0.6	2.8	0.6	0.2	1.1	0.7	0	0.1	0	1.3	0.7	0.8	0.7	3.2	0.1	0	1.4	0.1	1.3	1.4	1	0.2	0.3	0.3
Yuan, 2020	0.2	0.3	0.2	0.3	0.2	1.8	0.1	0.1	0.3	0	0	0	0	0.6	0	0	0	0.6	0	0	0.6	0	0.6	0.6	0.4	0.1	0.1	0.1
Zargoush, 2021	1.1	2.9	6.2	2.9	1.1	0.9	0.9	0.9	0.4	1.4	3.4	1.4	0.1	0.3	6.4	2.9	1.3	1.2	6.7	3.6	3.9	1.3	1.2	0.2	1.4	0.6	4	0.5
Zhang, 2021	0.9	2.9	0.9	2.9	0.9	3.7	1.1	1.1	1.9	1.1	0.1	1	0	2	1	2.9	1.1	4.3	1.2	0.1	2	1.1	4.2	2.1	1.5	0.4	0.4	0.4
Zhang 2, 2023	0.4	0.3	0.4	0.3	1.9	0.2	0	0	0	0	0	0	0.6	0.1	0	0	0.6	0.1	0	0.6	0.1	0.6	0.1	0.7	0.2	0	0	0.4
Zhang 3, 2023 (3h)	10.5	4.4	9.2	4.4	5.6	1.4	1.4	1.4	0.8	11.1	15	10.7	11.7	7.1	9.5	4.4	6.1	2	10	10.5	6.2	6	1.9	3.3	2.3	7	6.2	3.6
Zhang 3, 2023 (6h)	10.5	4.4	9.2	4.4	5.6	1.4	1.4	1.4	0.8	11.1	15	10.7	11.7	7.1	9.5	4.4	6.1	2	10	10.5	6.2	6	1.9	3.3	2.3	7	6.2	3.6

Abbreviations: DT, Decision Tree; NNM, Neural Network Model; SVM, Support Vector Machine; LR, Logistic Regression; NB, Naïve Bayes; GLM, Generalized Linear Model; KNN, K-Nearest Neighbors.

The rows refer to the studies, and columns refer to the comparisons (grouped into mixed and indirect estimates) from network meta-analysis. The entries show how much each study contributes (as percentage) to the estimation of the mean difference for comparisons. Mixed comparisons are estimated using both direct and indirect evidence.

Figure S1. Bubble plot depicting the univariate meta-regression of ML models AUCs with dataset used.

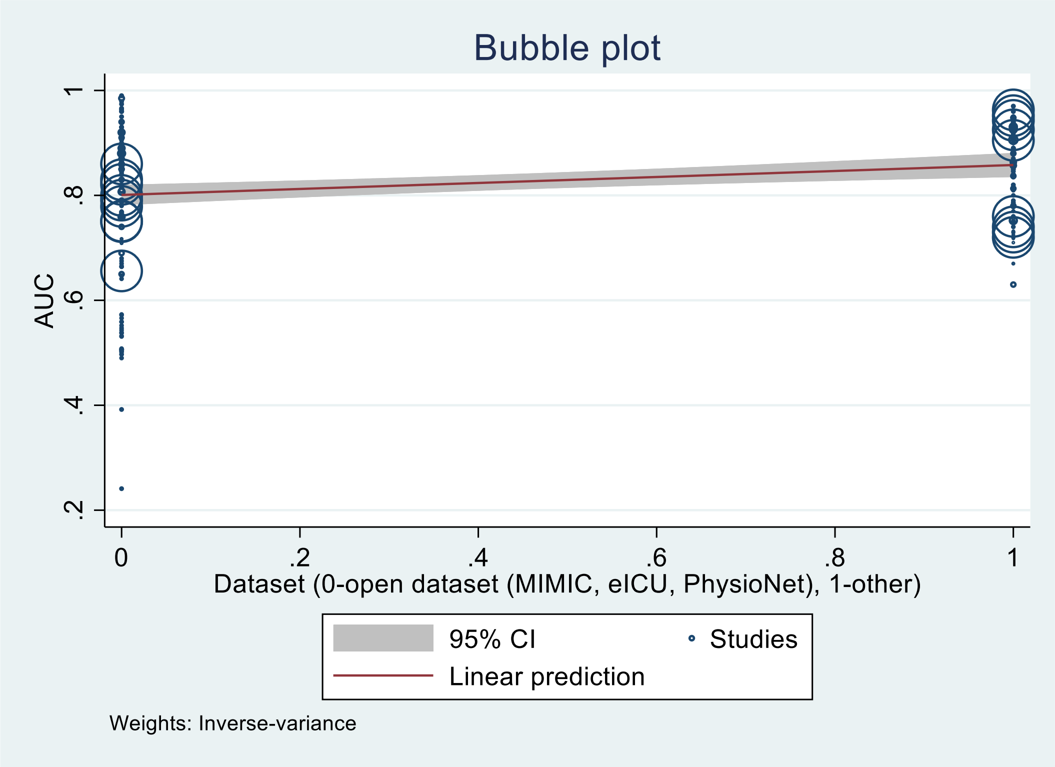

Figure S2. Bubble plot depicting the univariate meta-regression of ML models AUCs with prediction window.

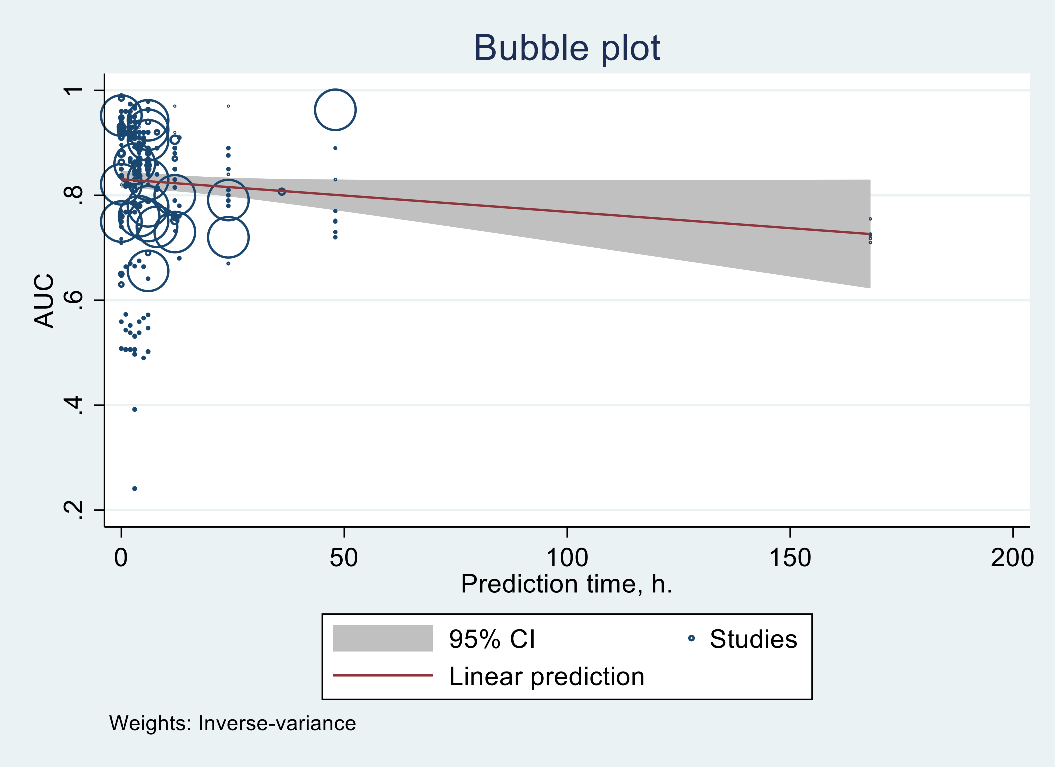

22 **Figure S3. Risk of bias evaluation of the included randomized controlled trials using QUADAS-2 tool.**

Study	PATIENT SELECTION	INDEX TEST	REFERENCE STANDARD	FLOW AND TIMING	DATA MANAGE MENT	OVERALL RISK OF BIAS
Abromavicius, 2020	☺	☺	☺	☺	☺	+
Amrollahi, 2020	☺	☺	☺	☺	☺	+
Aşurŏglu, 2020	☹	☺	☹	☺	☺	-
Bao, 2022	☹	☺	☺	☺	☺	!
Barton, 2019	☺	☺	☺	☺	☺	+
Bedoya, 2020	☺	☺	☹	☺	☺	!
Bloch, 2019	☺	☺	☹	☺	☺	!
Burdick, 2020	☺	☺	☹	☹	☺	-
Calvert, 2016	☹	☺	☹	☹	☺	-
Camacho-Cogollo, 2022	☹	☺	☺	☺	☺	!
Chen, 2021	☺	☺	☺	☺	☺	+
Chen, 2022	☺	☺	☺	☺	☺	+
Chen, 2023	☺	☺	☺	☺	☺	+
Choi, 2020	☹	☺	☹	☺	☹	-
Delahanty, 2019	☺	☺	☺	☺	☺	+
Desautels, 2016	☺	☺	☺	☺	☺	+
Duan. 2023	☹	☺	☹	☺	☺	-
El-Rashidy, 2022	☹	☺	☹	☹	☺	-
Fagerström, 2019	☺	☺	☹	☹	☺	-
Faisal, 2018	☺	☺	☹	☺	☺	!
Gholamzadeh, 2023	☹	☺	☺	☺	☺	!
Giannini, 2019	☺	☺	☹	☺	☺	!
Goh, 2021	☹	☺	☹	☺	☹	-
Horng, 2017	☺	☺	☹	☺	☺	!
Ibrahim, 2019	☹	☺	☹	☹	☹	-
Kaji, 2019	☹	☺	☹	☺	☺	-
Kam, 2017	☹	☺	☹	☹	☺	-
Khojandi, 2018	☺	☺	☹	☹	☺	-
Kijpaisalratana, 2022	☺	☺	☹	☹	☺	-
Kuo, 2021	☺	☺	☺	☺	☺	+
Kwon, 2021	☺	☺	☺	☺	☺	+
Lauritsen 1, 2020	☹	☺	☹	☺	☹	-
Lauritsen 2, 2021	☺	☺	☺	☺	☺	+
Li, 2020	☺	☺	☺	☺	☺	+
Lin, 2021	☹	☺	☺	☺	☺	!
Liu, 2022	☹	☺	☺	☺	☺	!
Liu, 2023	☺	☺	☺	☺	☺	+
Maharjan, 2022	☹	☺	☹	☹	☺	-
Mao, 2017	☺	☺	☹	☹	☺	-
McCoy, 2017	☹	☺	☺	☹	☺	-
Moor, 2023	☺	☺	☺	☺	☺	+
Nemati, 2018	☺	☺	☺	☺	☺	+
Nesaragi, 2021	☺	☺	☺	☺	☺	+
Oei, 2021	☺	☺	☹	☹	☺	-
Persson, 2021	☺	☺	☺	☺	☺	+
Rafiei, 2021	☺	☺	☺	☺	☺	+
Rangan, 2022	☺	☺	☺	☺	☺	+
Rosnati, 2021	☹	☺	☺	☺	☹	-
Sadasivuni, 2022	☹	☺	☺	☺	☹	-
Scherpf, 2019	☹	☺	☹	☹	☹	-
Shamoni, 2019	☺	☺	☹	☹	☹	-
Sharma, 2021	☺	☺	☺	☺	☺	+
Shashikumar 1, 2017	☹	☺	☺	☺	☹	-
Shashikumar 2, 2017	☹	☺	☺	☺	☹	-
Shashikumar 3, 2021	☹	☺	☺	☺	☺	!
Shimabukuro, 2017	☺	☺	☹	☹	☺	-
Singh, 2022	☹	☺	☹	☹	☹	-
Taneja, 2017	☺	☺	☺	☺	☹	!
Tang, 2021	☺	☺	☹	☹	☺	-
Valik, 2023	☺	☺	☺	☺	☺	+
Van Wyk, 2018	☹	☺	☹	☺	☺	-
Wang 1, 2021	☺	☺	☺	☺	☺	+
Wang 2, 2021	☺	☺	☺	☺	☺	+
Wong, 2021	☺	☺	☹	☹	☺	-
Yang 1, 2020	☺	☺	☺	☺	☺	+
Yang 2, 2022	☺	☺	☺	☺	☺	+
Yu, 2022	☺	☺	☺	☺	☺	+
Yuan, 2020	☺	☺	☺	☺	☺	+
Zargoush, 2021	☺	☺	☺	☺	☺	+
Zhang, 2021	☹	☺	☺	☺	☺	!
Zhang 2, 2023	☹	☺	☺	☺	☺	!
Zhang 3, 2023	☹	☺	☹	☺	☺	-
Zhao, 2021	☺	☺	☺	☺	☺	+

23 **Figure S4. Risk of bias bar chart for studied comparisons.**

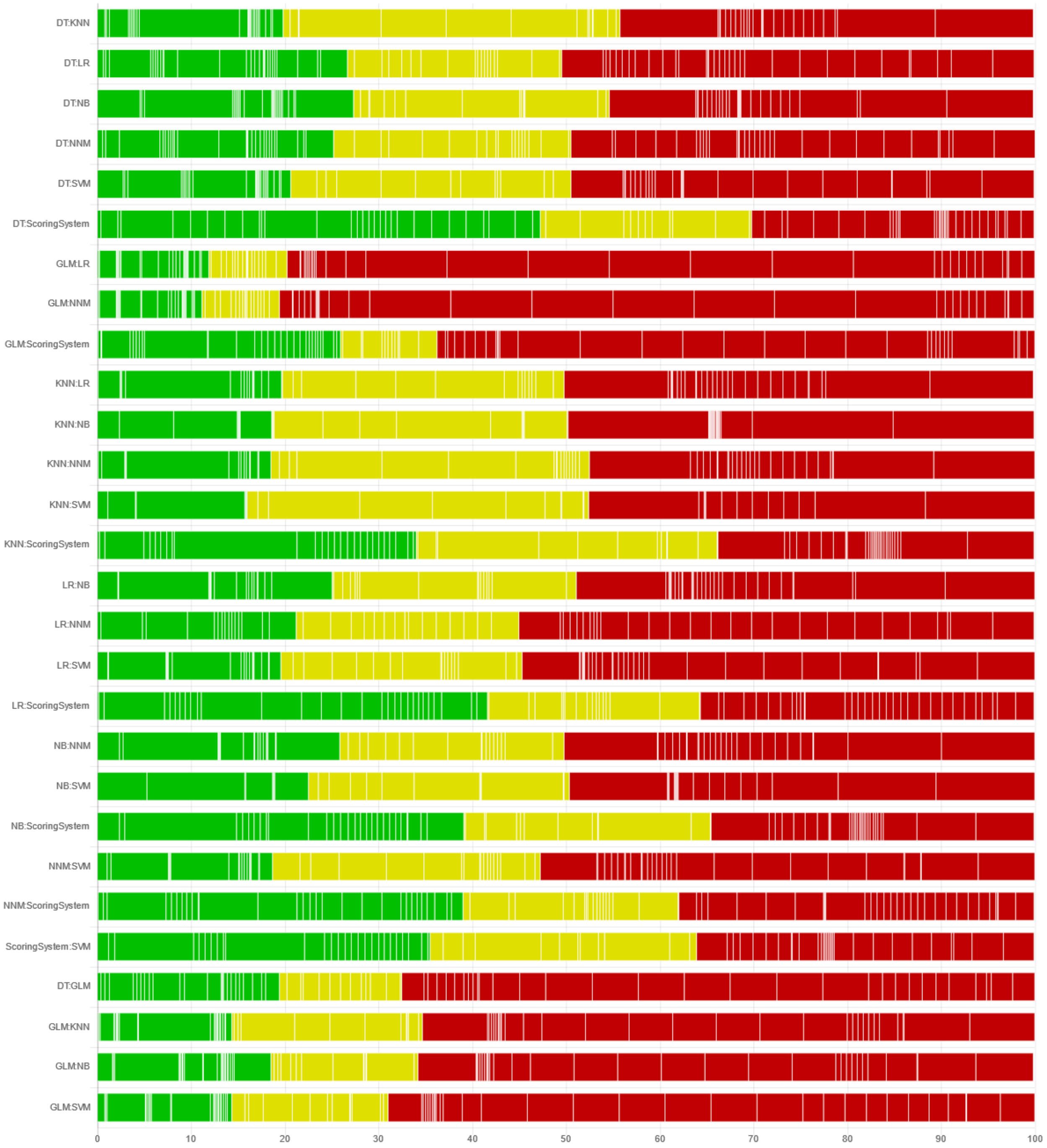

Abbreviations: DT, Decision Tree; NNM, Neural Network Model; SVM, Support Vector Machine; LR, Logistic Regression; NB, Naïve Bayes; GLM, Generalized Linear Model; KNN, K-Nearest Neighbors.

White vertical lines separate the percentage contribution of different studies. Each bar shows the percentage contribution from studies judged to be at low (green), moderate (yellow) and high (red) risk of bias.

24 **Figure S5. Network meta-analysis funnel plots for the assessment of publication bias of the included studies.**

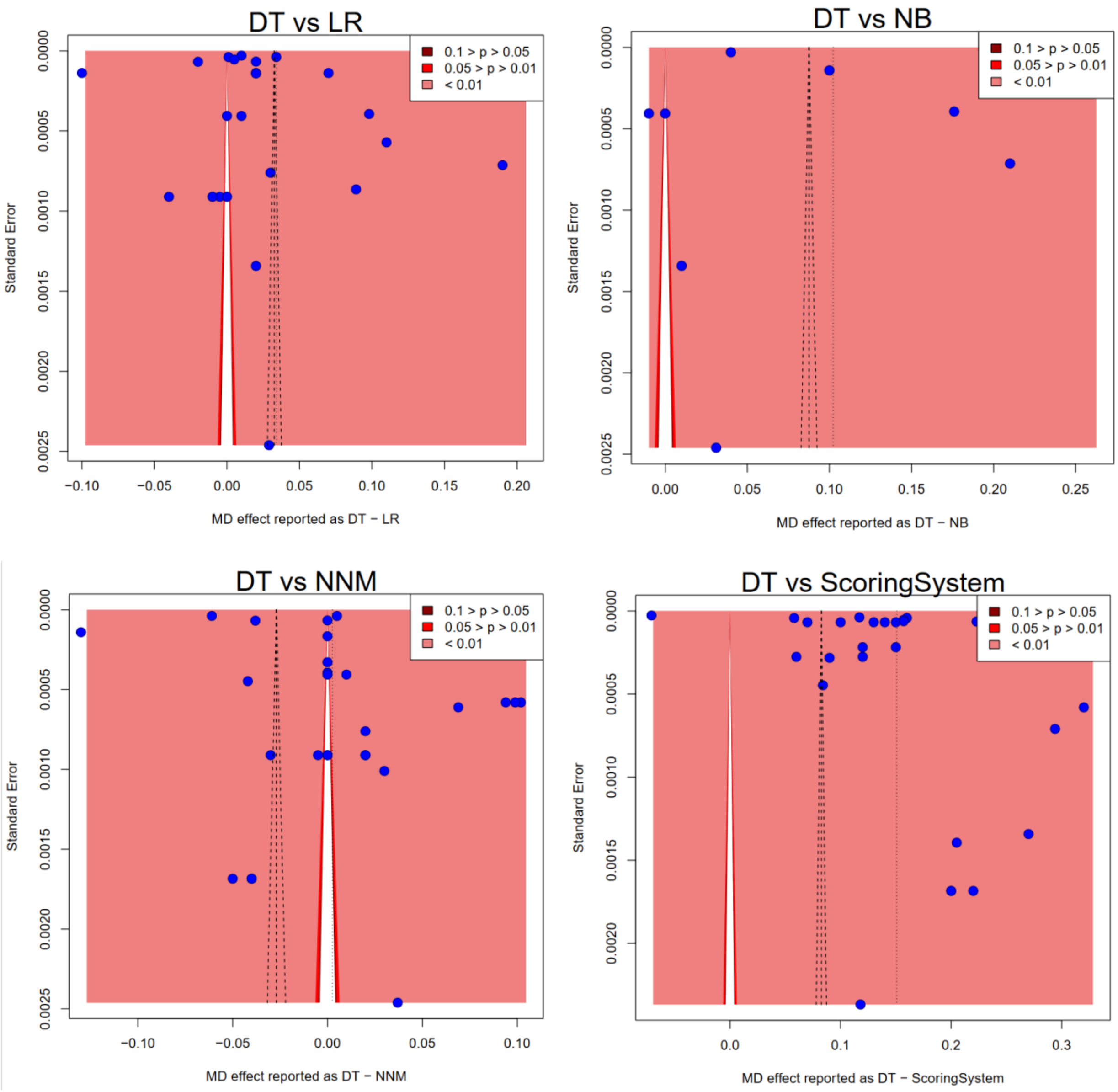

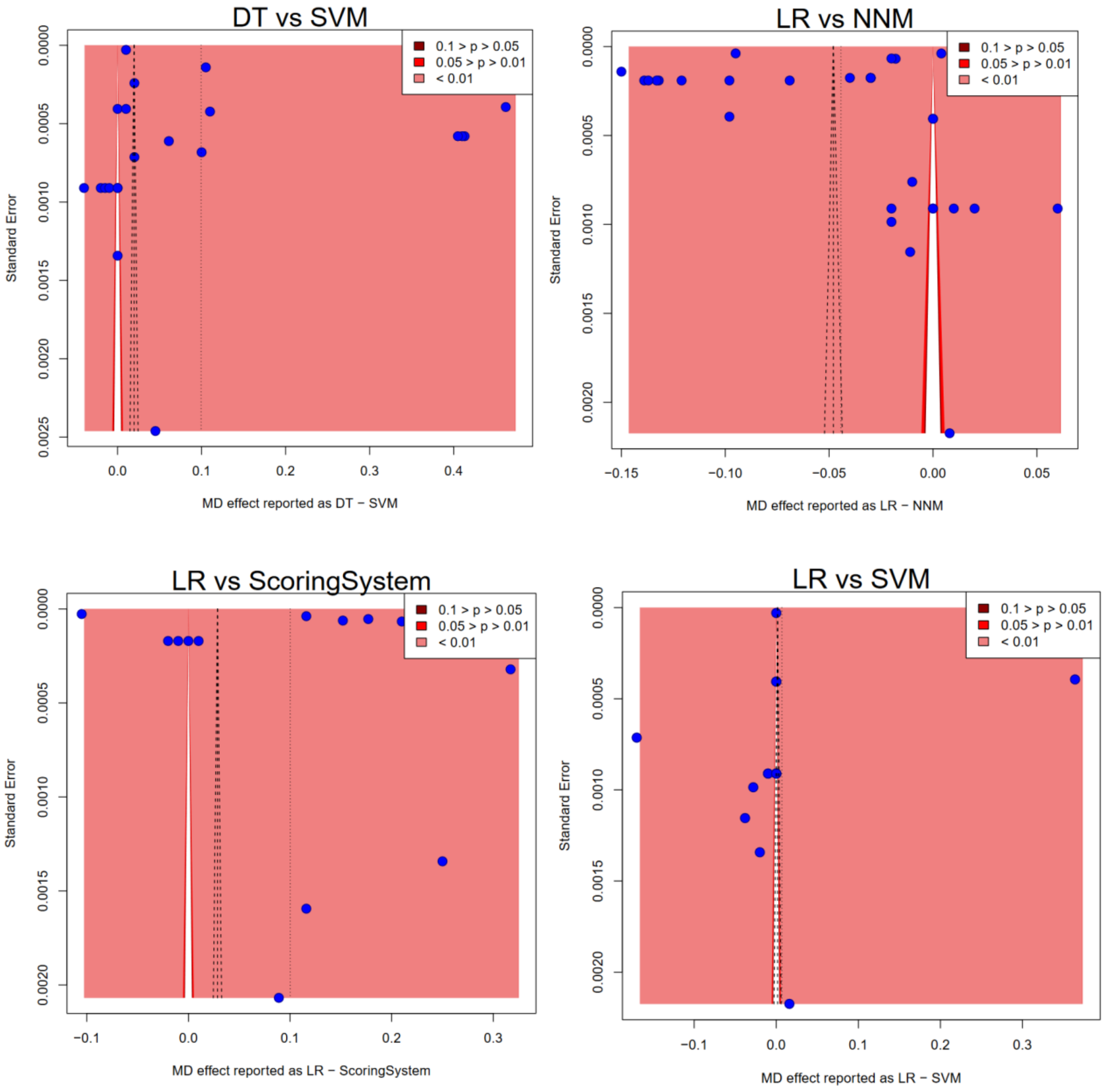

DT vs SVM
Standard Error
MD effect reported as DT − SVM
0.1 > p > 0.05
0.05 > p > 0.01
< 0.01
LR vs NNM
Standard Error
MD effect reported as LR − NNM
0.1 > p > 0.05
0.05 > p > 0.01
< 0.01
LR vs ScoringSystem
Standard Error
MD effect reported as LR − ScoringSystem
0.1 > p > 0.05
0.05 > p > 0.01
< 0.01
LR vs SVM
Standard Error
MD effect reported as LR − SVM
0.1 > p > 0.05
0.05 > p > 0.01
< 0.01

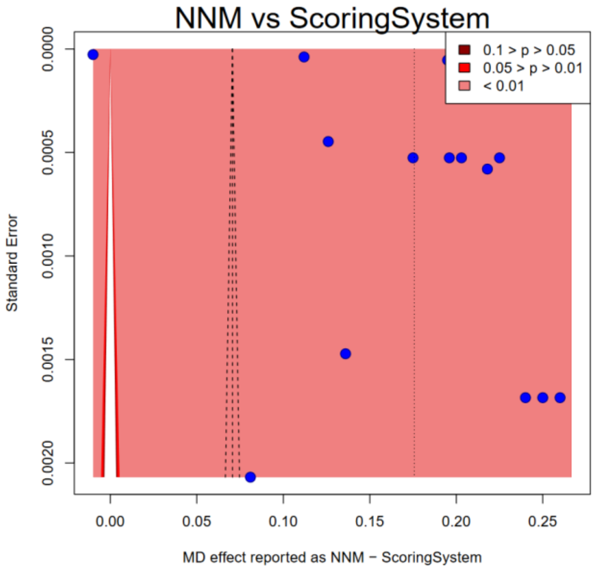

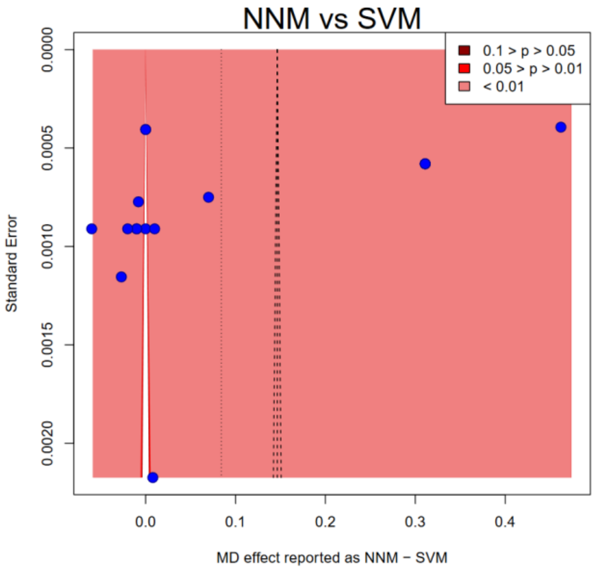

Abbreviations: DT, Decision Tree; NNM, Neural Network Model; SVM, Support Vector Machine; LR, Logistic Regression; NB, Naïve Bayes; GLM, Generalized Linear Model; KNN, K-Nearest Neighbors.

Networks are shown only for comparisons with 10 or more studies.

Figure S6. Certainty of evidence assessment (CINeMA approach).

Comparison	Number of Studies	Within-study bias	Reporting bias	Indirectness	Imprecision	Heterogeneity	Incoherence	Confidence rating	Reason(s) for downgrading
Mixed evidence									
DT vs KNN	8	Some concerns	Low risk	No concerns	No concerns	Major concerns	Major concerns	Very low	| Within-study bias | Heterogeneity | Incoherence
DT vs LR	27	Some concerns	Low risk	No concerns	No concerns	Major concerns	Some concerns	Very low	| Within-study bias | Heterogeneity | Incoherence
DT vs NB	10	Some concerns	Low risk	No concerns	No concerns	Some concerns	No concerns	Low	| Within-study bias | Heterogeneity |
DT vs NNM	27	Some concerns	Low risk	No concerns	No concerns	Major concerns	No concerns	Very low	| Within-study bias | Heterogeneity |
DT vs SVM	21	Some concerns	Some concerns	No concerns	No concerns	Some concerns	No concerns	Very low	| Within-study bias | Reporting bias | Heterogeneity
DT vs ScoringSystem	27	Some concerns	Low risk	No concerns	No concerns	Some concerns	No concerns	Low	| Within-study bias | Heterogeneity |
GLM vs LR	7	Major concerns	Low risk	No concerns	No concerns	Major concerns	No concerns	Very low	| Within-study bias | Heterogeneity |
GLM vs NNM	7	Major concerns	Low risk	No concerns	No concerns	Some concerns	Some concerns	Very low	| Within-study bias | Heterogeneity | Incoherence
GLM vs ScoringSystem	4	Some concerns	Low risk	No concerns	No concerns	Some concerns	No concerns	Low	| Within-study bias | Heterogeneity |
KNN vs LR	5	Some concerns	Low risk	No concerns	No concerns	Major concerns	No concerns	Very low	| Within-study bias | Heterogeneity |
KNN vs NB	4	Some concerns	Low risk	No concerns	No concerns	Major concerns	No concerns	Very low	| Within-study bias | Heterogeneity |
KNN vs NNM	7	Some concerns	Low risk	No concerns	No concerns	Major concerns	No concerns	Very low	| Within-study bias | Heterogeneity |
KNN vs SVM	7	Some concerns	Low risk	No concerns	No concerns	Major concerns	Major concerns	Very low	| Within-study bias | Heterogeneity | Incoherence
KNN vs ScoringSystem	2	Some concerns	Low risk	No concerns	No concerns	Some concerns	No concerns	Low	| Within-study bias | Heterogeneity |
LR vs NB	9	Some concerns	Low risk	No concerns	No concerns	Major concerns	No concerns	Very low	| Within-study bias | Heterogeneity |
LR vs NNM	30	Some concerns	Low risk	No concerns	No concerns	Some concerns	Some concerns	Very low	| Within-study bias | Heterogeneity | Incoherence
LR vs SVM	15	Some concerns	Low risk	No concerns	No concerns	Major concerns	No concerns	Very low	| Within-study bias | Heterogeneity |
LR vs ScoringSystem	13	Some concerns	Low risk	No concerns	No concerns	Some concerns	No concerns	Low	| Within-study bias | Heterogeneity |
NB vs NNM	5	Some concerns	Low risk	No concerns	No concerns	Some concerns	No concerns	Low	| Within-study bias | Heterogeneity |
NB vs SVM	8	Some concerns	Low risk	No concerns	No concerns	Major concerns	No concerns	Very low	| Within-study bias | Heterogeneity |
NB vs ScoringSystem	2	Some concerns	Low risk	No concerns	No concerns	Some concerns	No concerns	Low	| Within-study bias | Heterogeneity |
NNM vs SVM	16	Some concerns	Low risk	No concerns	No concerns	Some concerns	No concerns	Low	| Within-study bias | Heterogeneity |
NNM vs ScoringSystem	15	Some concerns	Low risk	No concerns	No concerns	Some concerns	No concerns	Low	| Within-study bias | Heterogeneity |
SVM vs ScoringSystem	4	Some concerns	Low risk	No concerns	No concerns	Some concerns	No concerns	Low	| Within-study bias | Heterogeneity |
Indirect evidence									
DT vs GLM	--	Some concerns	Low risk	No concerns	No concerns	Major concerns	Some concerns	Very low	| Within-study bias | Heterogeneity | Incoherence
GLM vs KNN	--	Major concerns	Low risk	No concerns	No concerns	Major concerns	Some concerns	Very low	| Within-study bias | Heterogeneity | Incoherence
GLM vs NB	--	Some concerns	Low risk	No concerns	No concerns	Major concerns	Some concerns	Very low	| Within-study bias | Heterogeneity | Incoherence
GLM vs SVM	--	Major concerns	Low risk	No concerns	No concerns	Major concerns	Some concerns	Very low	| Within-study bias | Heterogeneity | Incoherence

Abbreviations: DT, Decision Tree; NNM, Neural Network Model; SVM, Support Vector Machine; LR, Logistic Regression; NB, Naïve Bayes; GLM, Generalized Linear Model; KNN, K-Nearest Neighbors.

26 Supplemental references

1. Abromavičius V, Plonis D, Tarasevičius D, Serackis A. Two-stage monitoring of patients in intensive care unit for sepsis prediction using non-overfitted machine learning models. *Electron*. 2020;9(7):1-14. doi:10.3390/electronics9071133

2. Amrollahi F, Shashikumar SP, Razmi F, Nemati S. Contextual Embeddings from Clinical Notes Improves Prediction of Sepsis. *AMIA . Annu Symp proceedings AMIA Symp*. 2020;2020:197-202. Accessed November 24, 2023. /pmc/articles/PMC8075484/

3. Aşuroğlu T, Oğul H. A deep learning approach for sepsis monitoring via severity score estimation. *Comput Methods Programs Biomed*. 2021;198. doi:10.1016/j.cmpb.2020.105816

4. Bao C, Deng F, Zhao S. Machine-learning models for prediction of sepsis patients mortality. *Med Intensiva (English Ed*. 2023;47(6):315-325. doi:10.1016/j.medine.2022.06.024

5. Barton C, Chettipally U, Zhou Y, et al. Evaluation of a machine learning algorithm for up to 48-hour advance prediction of sepsis using six vital signs. *Comput Biol Med*. 2019;109:79-84. doi:10.1016/j.compbiomed.2019.04.027

6. Bedoya AD, Futoma J, Clement ME, et al. Machine learning for early detection of sepsis: An internal and temporal validation study. *JAMIA Open*. 2020;3(2):252-260. doi:10.1093/jamiaopen/ooaa006

7. Bloch E, Rotem T, Cohen J, Singer P, Aperstein Y. Machine Learning Models for Analysis of Vital Signs Dynamics: A Case for Sepsis Onset Prediction. *J Healthc Eng*. 2019;2019. doi:10.1155/2019/5930379

8. Burdick H, Pino E, Gabel-Comeau D, et al. Validation of a machine learning algorithm for early severe sepsis prediction: a retrospective study predicting severe sepsis up to 48 h in advance using a diverse dataset from 461 US hospitals. *BMC Med Inform Decis Mak*. 2020;20(1):1-10. doi:10.1186/s12911-020-01284-x

9. Calvert JS, Price DA, Chettipally UK, et al. A computational approach to early sepsis detection. *Comput Biol Med*. 2016;74:69-73. doi:10.1016/j.compbiomed.2016.05.003

10. Camacho-Cogollo JE, Bonet I, Gil B, Iadanza E. Machine Learning Models for Early Prediction of Sepsis on Large Healthcare Datasets. *Electron*. 2022;11(9):1507. doi:10.3390/electronics11091507

11. Chen M, Hernández A. Towards an Explainable Model for Sepsis Detection Based on Sensitivity Analysis. *Irbm*. 2022;43(1):75-86. doi:10.1016/j.irbm.2021.05.006

12. Chen Q, Li R, Lin CC, et al. Transferability and interpretability of the sepsis prediction models in the intensive care unit. *BMC Med Inform Decis Mak*. 2022;22(1):1-10. doi:10.1186/s12911-022-02090-3

13. Chen C, Chen B, Yang J, et al. Development and validation of a practical machine learning model to predict sepsis after liver transplantation. *Ann Med*. 2023;55(1):624-633.

doi:10.1080/07853890.2023.2179104

14. Choi JS, Trinh TX, Ha J, et al. Implementation of Complementary Model using Optimal Combination of Hematological Parameters for Sepsis Screening in Patients with Fever. *Sci Rep*. 2020;10(1):1-10. doi:10.1038/s41598-019-57107-1

15. Delahanty RJ, Alvarez JA, Flynn LM, Sherwin RL, Jones SS. Development and Evaluation of a Machine Learning Model for the Early Identification of Patients at Risk for Sepsis. *Ann Emerg Med*. 2019;73(4):334-344. doi:10.1016/j.annemergmed.2018.11.036

16. Desautels T, Calvert J, Hoffman J, et al. Prediction of sepsis in the intensive care unit with minimal electronic health record data: A machine learning approach. *JMIR Med Informatics*. 2016;4(3). doi:10.2196/medinform.5909

17. Duan Y, Huo J, Chen M, et al. Early prediction of sepsis using double fusion of deep features and handcrafted features. *Appl Intell*. 2023;53(14):17903-17919. doi:10.1007/s10489-022-04425-z

18. El-Rashidy N, Abuhmed T, Alarabi L, et al. Sepsis prediction in intensive care unit based on genetic feature optimization and stacked deep ensemble learning. *Neural Comput Appl*. 2022;34(5):3603-3632. doi:10.1007/s00521-021-06631-1

19. Fagerström J, Bång M, Wilhelms D, Chew MS. LiSep LSTM: A Machine Learning Algorithm for Early Detection of Septic Shock. *Sci Rep*. 2019;9(1):1-8. doi:10.1038/s41598-019-51219-4

20. Faisal M, Scally A, Richardson D, et al. Development and external validation of an automated computer-aided risk score for predicting sepsis in emergency medical admissions using the patient’s first electronically recorded vital signs and blood test results. *Crit Care Med*. 2018;46(4):612-618. doi:10.1097/CCM.0000000000002967

21. Gholamzadeh M, Abtahi H, Safdari R. Comparison of different machine learning algorithms to classify patients suspected of having sepsis infection in the intensive care unit. *Informatics Med Unlocked*. 2023;38:101236. doi:10.1016/j.imu.2023.101236

22. Giannini HM, Ginestra JC, Chivers C, et al. A Machine Learning Algorithm to Predict Severe Sepsis and Septic Shock: Development, Implementation, and Impact on Clinical Practice. *Crit Care Med*. 2019;47(11):1485-1492. doi:10.1097/CCM.0000000000003891

23. Goh KH, Wang L, Yeow AYK, et al. Artificial intelligence in sepsis early prediction and diagnosis using unstructured data in healthcare. *Nat Commun*. 2021;12(1):1-10. doi:10.1038/s41467-021-20910-4

24. Horng S, Sontag DA, Halpern Y, Jernite Y, Shapiro NI, Nathanson LA. Creating an automated trigger for sepsis clinical decision support at emergency department triage using machine learning. *PLoS One*. 2017;12(4):e0174708. doi:10.1371/journal.pone.0174708

25. Ibrahim ZM, Wu H, Hamoud A, Stappen L, Dobson RJB, Agarossi A. On classifying sepsis heterogeneity in the ICU: Insight using machine learning. *J Am Med Informatics Assoc*. 2020;27(3):437-443. doi:10.1093/jamia/ocz211

26. Kaji DA, Zech JR, Kim JS, et al. An attention based deep learning model of clinical events in the intensive care unit. *PLoS One*. 2019;14(2). doi:10.1371/journal.pone.0211057

27. Kam HJ, Kim HY. Learning representations for the early detection of sepsis with deep neural networks. *Comput Biol Med*. 2017;89:248-255. doi:10.1016/j.compbiomed.2017.08.015

28. Khojandi A, Tansakul V, Li X, Koszalinski RS, Paiva W. Prediction of Sepsis and In-Hospital Mortality Using Electronic Health Records. *Methods Inf Med*. 2018;57(4):185-193. doi:10.3414/ME18-01-0014

29. Kijpaisalratana N, Sanglertsinlapachai D, Techaratsami S, Musikatavorn K, Saoraya J. Machine learning algorithms for early sepsis detection in the emergency department: A retrospective study. *Int J Med Inform*. 2022;160:104689. doi:10.1016/j.ijmedinf.2022.104689

30. Kuo YY, Huang ST, Chiu HW. Applying artificial neural network for early detection of sepsis with intentionally preserved highly missing real-world data for simulating clinical situation. *BMC Med Inform Decis Mak*. 2021;21(1):1-11. doi:10.1186/s12911-021-01653-0

31. Kwon J myoung, Lee YR, Jung MS, et al. Deep-learning model for screening sepsis using electrocardiography. *Scand J Trauma Resusc Emerg Med*. 2021;29(1):1-12. doi:10.1186/s13049-021-00953-8

32. Lauritsen SM, Kalør ME, Kongsgaard EL, et al. Early detection of sepsis utilizing deep learning on electronic health record event sequences. *Artif Intell Med*. 2020;104:101820. doi:10.1016/j.artmed.2020.101820

33. Lauritsen SM, Thiesson B, Jørgensen MJ, et al. The Framing of machine learning risk prediction models illustrated by evaluation of sepsis in general wards. *npj Digit Med*. 2021;4(1):1-12. doi:10.1038/s41746-021-00529-x

34. Li X, Xu X, Xie F, et al. A Time-Phased Machine Learning Model for Real-Time Prediction of Sepsis in Critical Care. *Crit Care Med*. 2020;48(10):E884-E888. doi:10.1097/CCM.0000000000004494

35. Lin PC, Chen KT, Chen HC, Islam MM, Lin MC. Machine learning model to identify sepsis patients in the emergency department: Algorithm development and validation. *J Pers Med*. 2021;11(11):1055. doi:10.3390/jpm11111055

36. Liu Z, Khojandi A, Li X, Mohammed A, Davis RL, Kamaleswaran R. A Machine Learning–Enabled Partially Observable Markov Decision Process Framework for Early Sepsis Prediction. *INFORMS J Comput*. 2022;34(4):2039-2057. doi:10.1287/ijoc.2022.1176

37. Liu F, Yao J, Liu C, Shou S. Construction and validation of machine learning models for sepsis prediction in patients with acute pancreatitis. *BMC Surg*. 2023;23(1):1-13. doi:10.1186/s12893-023-02151-y

38. Maharjan J, Thapa R, Calvert J, et al. A New Standard for Sepsis Prediction Algorithms: Using Time-Dependent Analysis for Earlier Clinically Relevant Alerts. *SSRN Electron J*. Published online June 7, 2022. doi:10.2139/ssrn.4130480

39. Mao Q, Jay M, Hoffman JL, et al. Multicentre validation of a sepsis prediction algorithm using only vital sign data in the emergency department, general ward and ICU. *BMJ Open*. 2018;8(1):17833. doi:10.1136/bmjopen-2017-017833

40. McCoy A, Das R. Reducing patient mortality, length of stay and readmissions through machine learning-based sepsis prediction in the emergency department, intensive care unit and hospital floor units. *BMJ Open Qual*. 2017;6(2):e000158. doi:10.1136/bmjoq-2017-000158

41. Moor M, Bennett N, Plečko D, et al. Predicting sepsis using deep learning across international sites: a retrospective development and validation study. *eClinicalMedicine*. 2023;62:102124. doi:10.1016/j.eclinm.2023.102124

42. Nemati S, Holder A, Razmi F, Stanley MD, Clifford GD, Buchman TG. An Interpretable Machine Learning Model for Accurate Prediction of Sepsis in the ICU. *Crit Care Med*. 2018;46(4):547-553. doi:10.1097/CCM.0000000000002936

43. Nesaragi N, Patidar S. An Explainable Machine Learning Model for Early Prediction of Sepsis Using ICU Data. *Infect Sepsis Dev*. Published online October 27, 2021. doi:10.5772/intechopen.98957

44. Oei SP, van Sloun RJ, van der Ven M, Korsten HH, Mischi M. Towards early sepsis detection from measurements at the general ward through deep learning. *Intell Med*. 2021;5:100042. doi:10.1016/j.ibmed.2021.100042

45. Persson I, Östling A, Arlbrandt M, Söderberg J, Becedas D. A Machine Learning Sepsis Prediction Algorithm for Intended Intensive Care Unit Use (NAVOY Sepsis): Proof-of-Concept Study. *JMIR Form Res*. 2021;5(9). doi:10.2196/28000

46. Rafiei A, Rezaee A, Hajati F, Gheisari S, Golzan M. SSP: Early prediction of sepsis using fully connected LSTM-CNN model. *Comput Biol Med*. 2021;128:104110. doi:10.1016/j.compbiomed.2020.104110

47. Rangan ES, Pathinarupothi RK, Anand KJS, Snyder MP. Performance effectiveness of vital parameter combinations for early warning of sepsis - an exhaustive study using machine learning. *JAMIA Open*. 2022;5(4):1-11. doi:10.1093/jamiaopen/ooac080

48. Rosnati M, Fortuin V. MGP-AttTCN: An interpretable machine learning model for the prediction of sepsis. *PLoS One*. 2021;16(5 May):e0251248. doi:10.1371/journal.pone.0251248

49. Sadasivuni S, Saha M, Bhatia N, Banerjee I, Sanyal A. Fusion of fully integrated analog machine learning classifier with electronic medical records for real-time prediction of sepsis onset. *Sci Rep*. 2022;12(1):1-11. doi:10.1038/s41598-022-09712-w

50. Scherpf M, Gräßer F, Malberg H, Zaunseder S. Predicting sepsis with a recurrent neural network using the MIMIC III database. *Comput Biol Med*. 2019;113. doi:10.1016/j.compbiomed.2019.103395

51. Schamoni S, Lindner HA, Schneider-Lindner V, Thiel M, Riezler S. Leveraging implicit expert knowledge for non-circular machine learning in sepsis prediction. *Artif Intell Med*. 2019;100. doi:10.1016/j.artmed.2019.101725

52. Sharma DK, Lakhotia P, Sain P, Brahmachari S. Early prediction and monitoring of sepsis using sequential long short term memory model. *Expert Syst*. 2022;39(3):e12798. doi:10.1111/exsy.12798

53. Shashikumar SP, Stanley MD, Sadiq I, et al. Early sepsis detection in critical care patients using multiscale blood pressure and heart rate dynamics. *J Electrocardiol*. 2017;50(6):739-743. doi:10.1016/j.jelectrocard.2017.08.013

54. Shashikumar SP, Li Q, Clifford GD, Nemati S. Multiscale network representation of physiological time series for early prediction of sepsis. *Physiol Meas*. 2017;38(12):2235-2248. doi:10.1088/1361-6579/aa9772

55. Shashikumar SP, Josef CS, Sharma A, Nemati S. DeepAISE – An interpretable and recurrent neural survival model for early prediction of sepsis. *Artif Intell Med*. 2021;113:102036. doi:10.1016/j.artmed.2021.102036

56. Shimabukuro DW, Barton CW, Feldman MD, Mataraso SJ, Das R. Effect of a machine learning-based severe sepsis prediction algorithm on patient survival and hospital length of stay: A randomised clinical trial. *BMJ Open Respir Res*. 2017;4(1). doi:10.1136/bmjresp-2017-000234

57. Singh YV, Singh P, Khan S, Singh RS. A Machine Learning Model for Early Prediction and Detection of Sepsis in Intensive Care Unit Patients. *J Healthc Eng*. 2022;2022. doi:10.1155/2022/9263391

58. Taneja I, Reddy B, Damhorst G, et al. Combining Biomarkers with EMR Data to Identify Patients in Different Phases of Sepsis. *Sci Rep*. 2017;7(1):1-12. doi:10.1038/s41598-017-09766-1

59. Tang G, Luo Y, Lu F, et al. Prediction of Sepsis in COVID-19 Using Laboratory Indicators. *Front Cell Infect Microbiol*. 2021;10:586054. doi:10.3389/fcimb.2020.586054

60. Valik JK, Ward L, Tanushi H, et al. Predicting sepsis onset using a machine learned causal probabilistic network algorithm based on electronic health records data. *Sci Rep*. 2023;13(1):1-12. doi:10.1038/s41598-023-38858-4

61. van Wyk F, Khojandi A, Mohammed A, Begoli E, Davis RL, Kamaleswaran R. A minimal set of physiomarkers in continuous high frequency data streams predict adult sepsis onset earlier. *Int J Med Inform*. 2019;122:55-62. doi:10.1016/j.ijmedinf.2018.12.002

62. Wang D, Li J, Sun Y, et al. A Machine Learning Model for Accurate Prediction of Sepsis in ICU Patients. *Front Public Heal*. 2021;9:754348. doi:10.3389/fpubh.2021.754348

63. Wang Z, Yao B. Multi-Branching Temporal Convolutional Network for Sepsis Prediction. *IEEE J Biomed Heal Informatics*. 2022;26(2):876-887. doi:10.1109/JBHI.2021.3092835

64. Wong A, Otles E, Donnelly JP, et al. External validation of a widely implemented proprietary sepsis prediction model in hospitalized patients. *JAMA Intern Med*. 2021;181(8):1065-1070. doi:10.1001/jamainternmed.2021.2626

65. Yang M, Liu C, Wang X, et al. An Explainable Artificial Intelligence Predictor for Early Detection of Sepsis. *Crit Care Med*. 2020;48(11):E1091-E1096. doi:10.1097/CCM.0000000000004550

66. Yang D, Kim J, Yoo J, Cha WC, Paik H. Identifying the Risk of Sepsis in Patients with Cancer Using Digital Health Care Records: Machine Learning–Based Approach. *JMIR Med Informatics*. 2022;10(6). doi:10.2196/37689

67. Yu SC, Gupta A, Betthauser KD, et al. Sepsis Prediction for the General Ward Setting. *Front Digit Heal*. 2022;4:848599. doi:10.3389/fdgth.2022.848599

68. Yuan KC, Tsai LW, Lee KH, et al. The development an artificial intelligence algorithm for early sepsis diagnosis in the intensive care unit. *Int J Med Inform*. 2020;141. doi:10.1016/j.ijmedinf.2020.104176

69. Zargoush M, Sameh A, Javadi M, Shabani S, Ghazalbash S, Perri D. The impact of recency and adequacy of historical information on sepsis predictions using machine learning. *Sci Rep*. 2021;11(1). doi:10.1038/s41598-021-00220-x

70. Zhang D, Yin C, Hunold KM, Jiang X, Caterino JM, Zhang P. An interpretable deep-learning model for early prediction of sepsis in the emergency department. *Patterns*. 2021;2(2):100196. doi:10.1016/j.patter.2020.100196

71. Zhang S, Duan Y, Hou F, et al. Early prediction of sepsis using a high-order Markov dynamic Bayesian network (HMDBN) classifier. *Appl Intell*. 2023;53(22):26384-26399. doi:10.1007/s10489-023-04920-x

72. Zhang TY, Zhong M, Cheng YZ, Zhang MW. An interpretable machine learning model for real-time sepsis prediction based on basic physiological indicators. *Eur Rev Med Pharmacol Sci*. 2023;27(10):4348-4356. doi:10.26355/eurrev_202305_32439

73. Zhao X, Shen W, Wang G. Early Prediction of Sepsis Based on Machine Learning Algorithm. *Comput Intell Neurosci*. 2021;2021. doi:10.1155/2021/6522633
